# Well‐defined nanostructures of high entropy alloys for electrocatalysis

**DOI:** 10.1002/EXP.20230036

**Published:** 2024-07-02

**Authors:** Jie Chen, Liping Ren, Xin Chen, Qi Wang, Chunying Chen, Jinpeng Fan, Shuai Wang, Vasileios Binas, Shaohua Shen

**Affiliations:** ^1^ School of Chemical Engineering and Technology Xi'an Jiaotong University Xi'an China; ^2^ China Coal Energy Research Institute Co., Ltd. Xi'an Shaanxi China; ^3^ Institute of Electronic Structure and Laser (IESL) Heraklion Greece; ^4^ Physical Chemistry Laboratory Chemistry Department Aristotle University of Thessaloniki Thessaloniki Greece; ^5^ International Research Center for Renewable Energy State Key Laboratory of Multiphase Flow in Power Engineering Xi'an Jiaotong University Xi'an China

**Keywords:** electrocatalytic reactions, high‐entropy alloys, well‐defined nanostructures

## Abstract

High‐entropy alloys (HEAs) have attracted significant attention for electrocatalytic energy conversion by virtue of their promisingly high efficiency, stability, and low cost. Recently, encouraging progress has been made in tuning the structure and composition of HEAs used in electrolyzers and fuel cells. However, the understanding on the synthetic methods and the structure‐property‐performance relationship of well‐defined HEAs nanostructures is still inadequate. To gain insight into the future research directions on HEAs for electrocatalysis, in this paper, the synthetic methods commonly used to obtain well‐defined HEAs nanostructures (0D nanoparticles, 1D nanowires, 2D nanosheets/nanoplates, 3D nanoporous structures, and other three‐dimensional morphologies) are first summarized. Then, the authors discuss the application of well‐defined HEAs nanostructures in several typical electrocatalytic reactions, including hydrogen evolution reaction, oxygen evolution reaction, oxygen reduction reaction, alcohol oxidation reaction, carbon dioxide reduction reaction, nitrogen reduction reaction, and formic acid oxidation reaction. Finally, a practical perspective on the future research directions on well‐defined HEAs nanostructured electrocatalysts is provided.

## Graphical abstract

This paper summarizes and discusses the synthetic methods used to obtain well‐defined HEAs nanostructures (0D nanoparticles, 1D nanowires, 2D nanosheets/nanoplates, 3D nanoporous structures, and other 3D morphologies) for the applications in several typical electrocatalytic reactions, including hydrogen evolution reaction, oxygen evolution reaction, oxygen reduction reaction, alcohol oxidation reaction, carbon dioxide reduction reaction, nitrogen reduction reaction, and formic acid oxidation reaction.

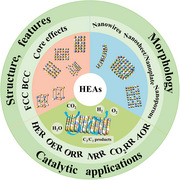

## 1 INTRODUCTION

The development of modern society is limited by the depletion of fossil fuels.^[^ ^1^ ^]^ Tremendous efforts have thus been made to discover approaches to providing renewable fuels and electricity.^[^ ^2^, ^3^ ^]^ In this case, electrocatalytic energy conversion technology (EECT) has been developed to supply fuels as well as electricity in a low‐carbon emission manner.^[^ ^4^ ^]^ Through EECT, small molecules (such as H_2_O, CO_2_, N_2_, alcohols, etc.) can be converted to value‐added chemicals via an electrolyzer stack, and vice versa, chemicals (such as H_2_, HCOOH, etc.) can be converted to electricity via fuel cells. The reduction reactions involved in EECT include hydrogen evolution reaction (HER) that generates hydrogen from water,^[^ ^5^ ^]^ oxygen reduction reaction (ORR) that happens at the cathodes in fuel cells,^[^ ^6^, ^7^ ^]^ carbon dioxide reduction reaction (CO_2_RR) that enables carbon capture and utilization^[^ ^8^ ^]^ and nitrogen reduction reaction (NRR) that produces NH_3_ from atmospheric N_2_.^[^ ^9^ ^]^ The oxidation reactions in EECT include hydrogen oxidation reaction (HOR) for fuel cells, oxygen evolution reaction (OER),^[^ ^10^, ^11^ ^]^ and methanol/ethanol/glycerol/formic acid oxidation reaction (MOR/EOR/GOR/FAOR) that selectively produces value‐added chemicals and electricity.^[^ ^12^ ^]^ Despite the versatility of EECT in producing renewable fuels and electricity, the practical applications of EECT are still limited, due to the lack of high‐performance electrocatalysts that can stably operate in wide oxidative/reductive potential windows with fast reaction kinetics.^[^ ^4^ ^]^

**FIGURE 1:**
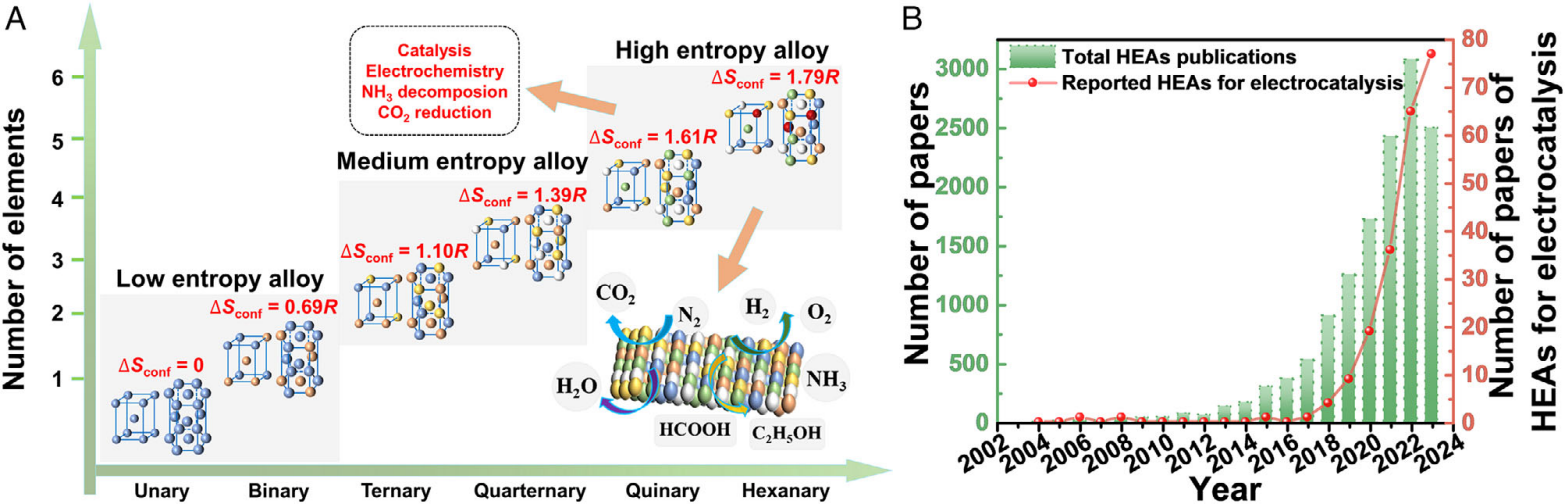
The configurational entropy of alloys, the applications of HEAs, and the number of publications. (A) The configurational entropy of alloys with different element numbers and applications of HEAs. (B) Number of papers containing “High‐Entropy Alloys (HEAs)” (bar chart) and number of papers of HEAs for electrocatalysis (line graph) from the database of Web of Science since 2004 (Data obtained on December 2023).

In recent years, significant efforts have been made to the development of electrocatalysts with high performance (i.e., high efficiency, long‐term stability, and good selectivity). However, the high performance of many electrocatalytic materials relies on a high content of precious metals acting as the active sites, which limits their up‐scaling applications.^[^ ^7^, ^11^ ^]^ Low‐cost and earth‐abundant transition metal‐containing materials have thus been extensively studied as promising alternatives to noble metal‐based catalysts.^[^ ^13^ ^]^ Among the as‐developed electrocatalysts, alloys formed by precious metals mixing with non‐precious transition metals with cheap prices have shown high activity, stability, and selectivity by virtue of their specific electronic properties and synergistic interactions between the alloyed components and/or structures.^[^ ^14^, ^15^ ^]^ Nevertheless, the efficiency and stability of the binary or ternary alloy electrocatalysts are still not satisfactory, largely due to the finite and simple component combinations of the alloys.^[^ ^2^, ^16^ ^]^ To break this limitation, a unique alloy system—high entropy alloys (HEAs)—has emerged as a kind of possible high‐performance electrocatalysts (Figure 1A) due to their theoretically high stability and the cocktail effect that brings up intriguing new properties and possibilities for multifunctional electrocatalysis. It should be noted that HEAs have literally received a rocket‐like research attention since 2004 in the field of electrocatalysis (Figure 1B).^[^ ^17^ ^]^

Well‐defined nanostructured materials have raised significant attention because of their intriguing properties that are beneficial to electrocatalysis. For instance, 0D catalysts can offer precise active sites thus maximizing the atomic utilization of the material.^[^ ^18^ ^]^ 1D catalysts with a high aspect ratio can provide a directional electron transfer path, thereby improving conductivity and accelerating reaction kinetics.^[^ ^19^ ^]^ 2D catalysts, such as nanoplates, have a high anisotropy, large active surface area, high electrical conductivity, and structural stability.^[^ ^20^ ^]^ 3D catalysts, taking nanoporous materials as an example, have a variety of unique properties (i.e., open pore structure and adjustable porosity), which are favorable for mass and energy transfer.^[^ ^21^ ^]^ Interestingly, confined spaces in 3D nanoporous structures can tune the diffusion flux of reactants and/or products, thereby improving the selectivity of target products.

Benefiting from the joint merits of well‐defined nanostructures and high‐entropy, well‐defined HEAs nanostructures will provide fascinating opportunities for EECT. In view of the complex component library of HEAs, the configuration entropy increases exponentially as the number of components increases (described in detail in the concept of HEAs in Section 2.1). The single solid solution phase with uniform element distribution in HEAs tends to be obtained at high temperatures. The strategy of high temperature as a driven force facilitates the synthesis of HEAs nanoparticles in a wide compositional range, but the accurate construction of multi‐dimensional morphologies is still limited.^[^ ^22^ ^]^ Due to the lack of comprehensive theoretical simulation to guide the selection of elements and ratios, and the absence of universal methods for synthesizing multi‐dimensional HEAs catalysts, the design and application of well‐defined HEAs nanostructures are still in their infancy. Currently, seldom of the reviews have aimed at the state‐of‐the‐art well‐defined HEAs nanostructures regarding the synthetic methodology and the structure‐property‐performance relationship in electrocatalysis.

Herein, we summarize the current research achievements and discuss the challenges of well‐defined HEAs nanostructures in the field of EECT. The concept, structures, and key effects of HEAs are introduced in Section 2. Then, the synthetic methods of different types of well‐defined HEAs nanostructures (including 0D nanoparticles in Section 3, 1D nanowires in Section 4, 2D nanosheets/nanoplates in Section 5, 3D nanopores and other nanostructures in Section 6) and their structure‐property‐performance relationships in various types of electrocatalytic reactions (including reduction reactions: HER, ORR, CO_2_RR, NRR, and oxidation reactions: OER, MOR/EOR/GOR/FAOR) are summarized and discussed in detail. Finally, the opportunities and challenges of HEAs for electrocatalysis are proposed in Section 7.

## 2 DEFINITION, STRUCTURE, AND FEATURES OF HEAS

### 2.1 Definition of HEAs

The concept of HEAs was first reported in 2004 by two independent studies.^[^ ^23^, ^24^ ^]^ Yeh and co‐workers reported that equimolar alloying of five metals will result in a unique and disordered microstructure.^[^ ^23^ ^]^ The author proposed that the formation of a stable solid solution phase is driven by the dominant contribution of atomic disorder to the overall Gibbs free energy, surpassing that of enthalpy. In the same year, Cantor et al. reported HEAs independently in another study, but described this 5‐component single‐phase alloy as a “multi‐component alloy” instead of a “high‐entropy alloy.”^[^ ^24^ ^]^

For now, HEAs can be defined either by the configurational entropy criteria or by the elemental composition criteria. According to the configuration entropy criteria, HEAs can be defined by the value of configuration entropy change (Δ*S_conf_*) of a certain alloy system. Entropy is usually used to depict the degree of system disorder in thermodynamics.^[^ ^25^ ^]^ According to the relationship between entropy and thermodynamics, entropy can be described by Equation (1).

(1) S=klnw

where *k* is the Boltzmann constant, and *w* represents the number of micro‐states of the system.

The Δ*S_conf_* of *n* elements with equimolar during the formation of solid solution can be calculated according to Equation (2).^[^ ^23^ ^]^

(2) $$\begin{array}{ccc} \Delta S_{conf} & = & -R[x_{1}\ln x_{1}+x_{2}\ln x_{2}+\cdot \cdot \cdot +x_{n}\ln x_{n}]\\ & = & -R\sum_{i=1}^{n}x_{i}\ln x_{i} \end{array}$$

In Equation (2), *R* represents the molar gas constant, of which the value is 8.314 J K^−1^ mol^−1^, $x_{i}$ represents the molar percentage of the *i* ^th^ component, and *n* represents the number of elements. Therefore, the configurational entropy of the system can be maximized when the mole percent of each element is equal. Hence, in a single‐phase solid solution structure with equimolar ratio, the Δ*S_conf_* formula of HEAs can be simplified to Equation (3).^[^ ^26^ ^]^

(3) $$\Delta S_{conf}=R\ln n$$

When *n* is 2, 3, 5, 6, and 13, the Δ*S_conf_* of the alloy system is calculated to be 0.69R, 1.10R, 1.61R, 1.79R, and 2.57R, respectively. As classified by the value of configurational entropy, a single‐phase solid solution can be named as low entropy (Δ*S_conf_* < 0.69R) alloy, medium entropy (0.69R < Δ*S_conf_* < 1.61R) alloy, and high entropy (Δ*S_conf_* > 1.61R) alloy, respectively.^[^ ^27^, ^28^ ^]^

According to the elemental composition criteria, alloy solid solution systems composed of more than five major elements with each of the elements having an atomic percentage of between 5% and 35% can be defined as HEAs.^[^ ^23^ ^]^ The detailed description is shown in Equations (4) and (5).

(4) $$n_{major}\geq 5,5\mathrm{at}\%\leq c_{i}\leq 35\mathrm{at}\%$$

(5) $$n_{minor}\geq 0,c_{j}\leq 5\mathrm{at}\%$$

where *n_major_* and *n_minor_* are the number of main and secondary elements, respectively. *c_i_* and *c_j_* represent the corresponding atomic percentages of the elements in the system.^[^ ^29^ ^]^

HEAs do not contain any elements with atomic percentage of higher than 50 at%, which is different from the conventional alloys. Moreover, when the number of elements exceeds 13, the entropy increment is relatively slow, hence, HEAs are preferentially consisting of 5 to 13 metal elements.^[^ ^28^ ^]^

### 2.2 Crystal structure and properties of HEAs

**FIGURE 2:**
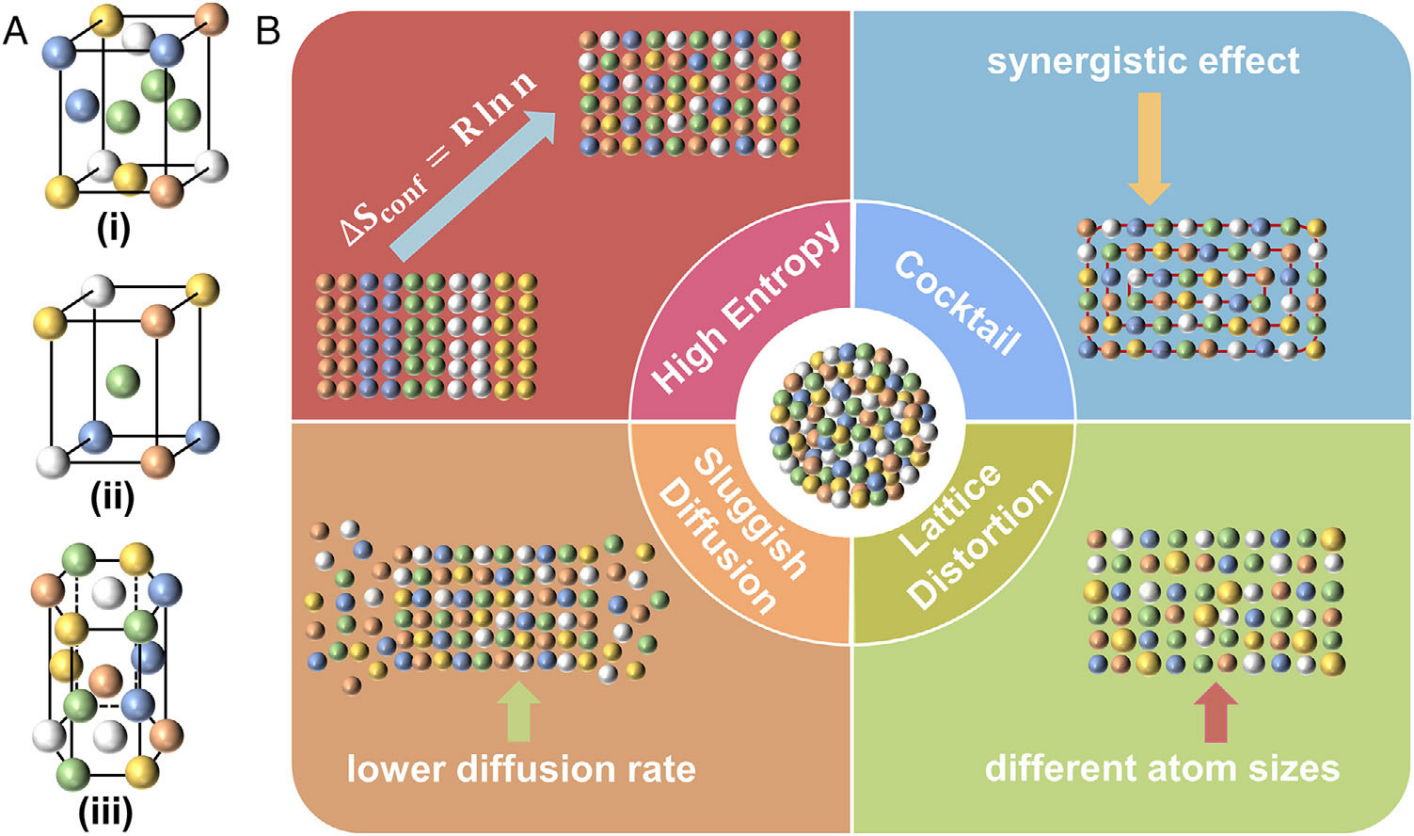
The crystal structures and core effects of HEAs. (A) Typical structure types of HEAs: (i) face‐centered cubic (FCC) crystal structure, (ii) body‐centered cubic (BCC) crystal structure, (iii) hexagonal close‐packed (HCP) crystal structure. (B) Schematic illustration summarizing the properties of HEAs.

Typical HEA crystal structure types include face‐centered cubic (FCC), body‐centered cubic (BCC), and hexagonal close‐packed (HCP), as illustrated in Figure 2A. Different from traditional alloys, each element in HEAs is randomly and uniformly distributed in the lattice. Compared to binary and ternary alloys, HEAs exhibit unique properties in terms of thermodynamics (high entropy effect), kinetics (sluggish diffusion effect), crystal structure (lattice distortion effect), and the cocktail effect.^[^ ^14^, ^30^ ^]^ Their unique properties bring many advantages for electrocatalysis (Figure 2B), which will be discussed in detail as follows.

#### 2.2.1 High‐entropy effect of HEAs

HEAs have a high mixed configurational entropy, which ensures that mixed polymetallic components can co‐exist in one alloy. The Gibbs free energy (Δ*G_mix_*) of HEAs is calculated according to Δ*G_mix_* = Δ*H_mix_* − *T*Δ*S_mix_*. It was found that a large configuration entropy is favorable for reducing the Δ*G_mix_* of the HEAs system. Meanwhile, a large configuration entropy favors the formation of a stable single‐phase solid solution structure rather than the brittle intermetallic compounds, thus contributing to the stability of HEAs.^[^ ^25^, ^31^ ^]^ According to the law of thermodynamics, the high entropy effect can prevent the formation of intermetallic compounds and phase separation, because a high Δ*S_mix_* means that the distribution of the components in the lattice is arbitrary.^[^ ^32^ ^]^

#### 2.2.2 Lattice distortion effect of HEAs

The dislocation of the lattice atoms from their ideal position, which is caused by the disparity in atomic size, leads to lattice distortion. The lattice distortion effect of HEAs increases the hardness and thermal stability and tunes the electronic structure of the material. Moreover, the lattice distortion causes lattice strain in HEAs, which alters the *d*‐band center and thus impacts the adsorption energy of reactants and intermediates,^[^ ^33^ ^]^ thereby tuning the catalytic performance.

#### 2.2.3 Sluggish diffusion effect of HEAs

The interaction between the atoms in a crystal will affect the activation and migration energy barriers of each atom. The lattice potential energy at diverse positions in the HEAs crystal is different, resulting in higher atom diffusion activation energy, thus inhibiting the diffusion and diminishing the effective rate of diffusion.^[^ ^34^ ^]^ The key difference between the atom diffusion in HEAs and the atom diffusion in traditional alloys is that each site within the HEAs lattice is encompassed by dissimilar atoms,^[^ ^35^ ^]^ making the chemical environment around each atom in the HEAs much more complex than that in the traditional alloys. The sluggish diffusion effect plays a critical role because it leads to high‐temperature structural stability of HEAs.^[^ ^36^ ^]^

#### 2.2.4 Cocktail effect of HEAs

The cocktail effect refers to the synergistic effect due to the interaction of various elements. This effect may render HEAs to have some new characteristics with respect to the mechanical properties, corrosion resistance, oxidation resistance, and electrocatalytic properties that are not possessed by any individual component.^[^ ^37^ ^]^

For electrocatalysis, the high entropy and sluggish diffusion effect will work together to enhance the thermodynamic stability of HEAs electrocatalysts. The lattice distortion effect is likely to tune the coordination environment of the catalyst surface atoms or optimize the adsorption energy of key reaction intermediates.

## 3 SYNTHESIS AND APPLICATIONS OF 0D HEAS NANOPARTICLES FOR ELECTROCATALYSIS

The success of HEAs in electrocatalysis benefits from the development of synthetic methodology of HEAs.^[^ ^38^ ^]^ Herein, the synthetic methodology and structure‐property‐performance relationships of 0D HEAs nanoparticles (NPs) in electrocatalytic reactions (including HER, OER, ORR, CO_2_RR, NRR, as well as MOR/EOR/GOR/FAOR) are summarized and discussed in detail.

### 3.1 The synthetic methods of HEAs nanoparticles

For an electrocatalytic reaction, reducing the size of the HEA‐based catalysts to the nanometer level to increase the specific surface area is helpful for enhancing its catalytic activity. In nanoscale materials, the spherical morphology of HEAs nanoparticles has lower formation energy compared to other morphologies and is therefore more easily accessed. The synthetic methods of HEAs NPs will be discussed as follows.

#### 3.1.1 Mechanical alloying method

The mechanical alloying methods include ball milling, arc melting, and laser cladding, etc. In general, these methods involve processes where metal salts or bulk metals form micro‐nano powders directly from their solid phases.

**FIGURE 3:**
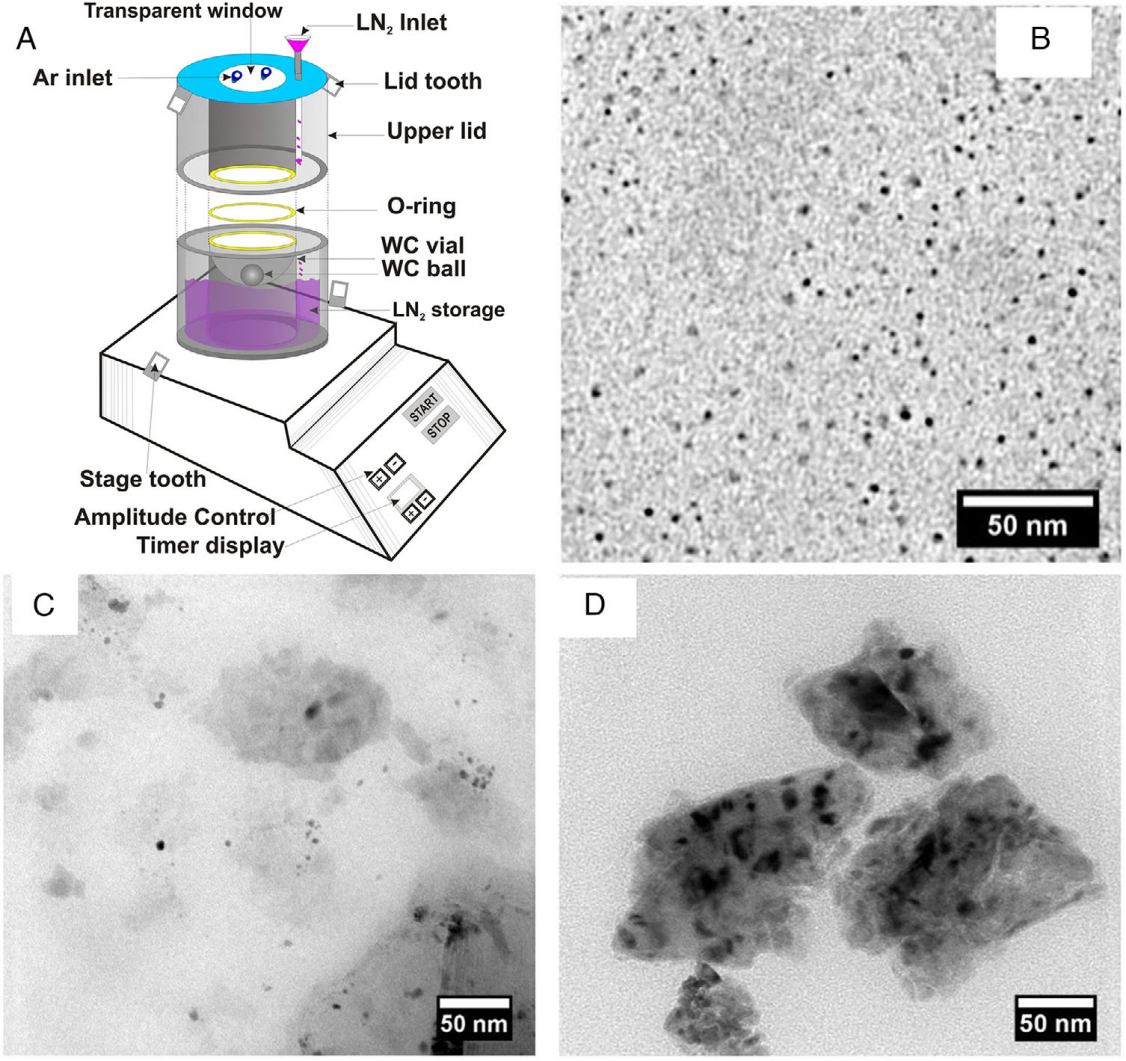
Synthesis and TEM images of HEAs nanoparticles by cryo‐milling method. (A) Schematic of custom‐designed cryo‐mill with a single ball made of Tungsten carbide (WC). (B) TEM image of Fe_0.2_Cr_0.2_Mn_0.2_Ni_0.2_Co_0.2_ stabilized using oleylamine as the capping agent. (C) TEM image of Cu_0.2_Ag_0.2_Au_0.2_Pt_0.2_Pd_0.2_. (D) TEM image of Fe_0.2_Cr_0.2_Mn_0.2_V_0.2_Al_0.2_. Reproduced with permission.^[^ ^39^ ^]^ Copyright 2018, Springer Nature.

Ball milling is to grind large bulks of the materials in a ball mill until small particles are formed. The synthesis of HEAs NPs, however, necessitates an extended duration of ball milling, which inadvertently introduces impurities from the surrounding atmosphere. To alleviate the contamination, the ball cryo‐milling method was applied to grind the materials at a low temperature (Figure 3A). Cryo‐milling could reduce the pollution, oxidation, and recrystallization of the product. Kumar et al. synthesized HEA with different components via the cryo‐milling method (Figure 3B–D).^[^ ^39^ ^]^ The HEA was found to be at the nanometer scale with high yield and high purity. The discharge/laser cladding method has also been reported for the synthesis of HEAs. For example, Wu et al. synthesized CoCrFeNiPt and CoFeNiCr_0.5_Pd_0.8_ HEA with uniform element distribution by the arc melting and discharge method.^[^ ^40^ ^]^ In specific, under the condition of spark discharge, the atoms of the CoCrFeNiPt and CoFeNiCr_0.5_Pd_0.8_ HEAs electrodes were evaporated and condensed into HEAs NPs upon cooling down.

#### 3.1.2 Carbon thermal shock

**FIGURE 4:**
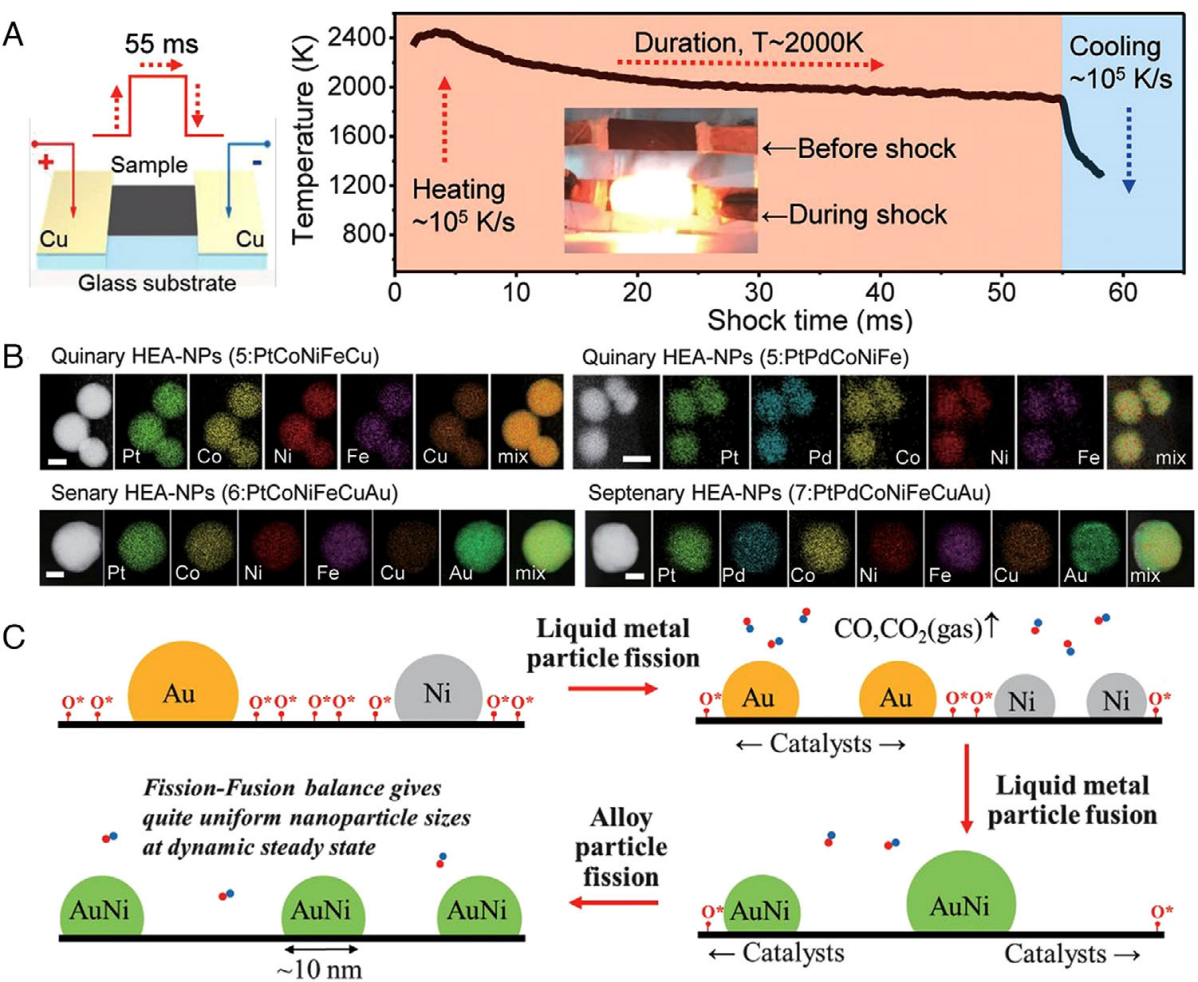
Synthesis and TEM images of HEAs nanoparticles by thermal shock method. (A) Time variation of sample preparation and temperature during thermal shock. (B) HAADF images and elemental maps of PtCoNiFeCu, PtPdCoNiFe, PtCoNiFeCuAu, and PtPdCoNiFeCuAu. Scale bar, 10 nm. (C) An illustration of the catalysis‐driven particle fission/fusion mechanism. Reproduced with permission.^[^ ^41^ ^]^ Copyright 2018, American Association for the Advancement of Science (HEA‐NPs: high entropy alloy nanoparticles).

It is a grand challenge to controllably combine multiple immiscible elements into one single nanoparticle using traditional synthetic techniques. The development of a synthetic method capable of precisely controlling the element composition, particle size, and phase could lead to a new array of alloys and nanostructures with unparalleled capabilities. Yao et al. developed a carbon thermal shock (CTS) method (Figure 4A), and used a two‐step CTS method to rapidly heat and cool the metal precursor (temperature ≈ 2000 K, shock duration ≈ 55 ms, slope rate 10^5^ K s^−1^) on the oxygenated carbon support.^[^ ^41^ ^]^ HEAs NPs containing up to seven metal elements were obtained (Figure 4B). The HEAs NPs synthesized via CTS with a narrow and uniform profile dispersed on the carbon support. It was found that the homogeneous mixture of various elements is formed by high temperatures combined with the catalytic activity of liquid metals to drive fast “fission” and “fusion” of particles. As demonstrated in Figure 4C, a larger surface‐bound residual oxygen (O*) concentration, coupled with the usage of catalytically active metals, can facilitate vigorous metabolic activity through frequent catalyst movement and fission events. During high temperature shocks with an operating time of 55 ms, the fusion and fission events of particles occurred for more than 10^6^ times, yielding a dynamic steady state, which enabled uniform dispersion of particles at the nanoscale and homogeneous mixing with high‐entropy. This work was also verified by the synthesis of quinary HEA‐NPs (PtFeCoNiCu, particle size of 5.30 ± 1.31 nm) on CO_2_‐activated carbon nanofibers. The CTS method opens a broad space for the synthesis of HEAs NPs. However, CTS method is limited to the synthesis of HEAs NPs with metal precursors that can be loaded on the carbon support.

#### 3.1.3 Fast‐moving bed pyrolysis

**FIGURE 5:**
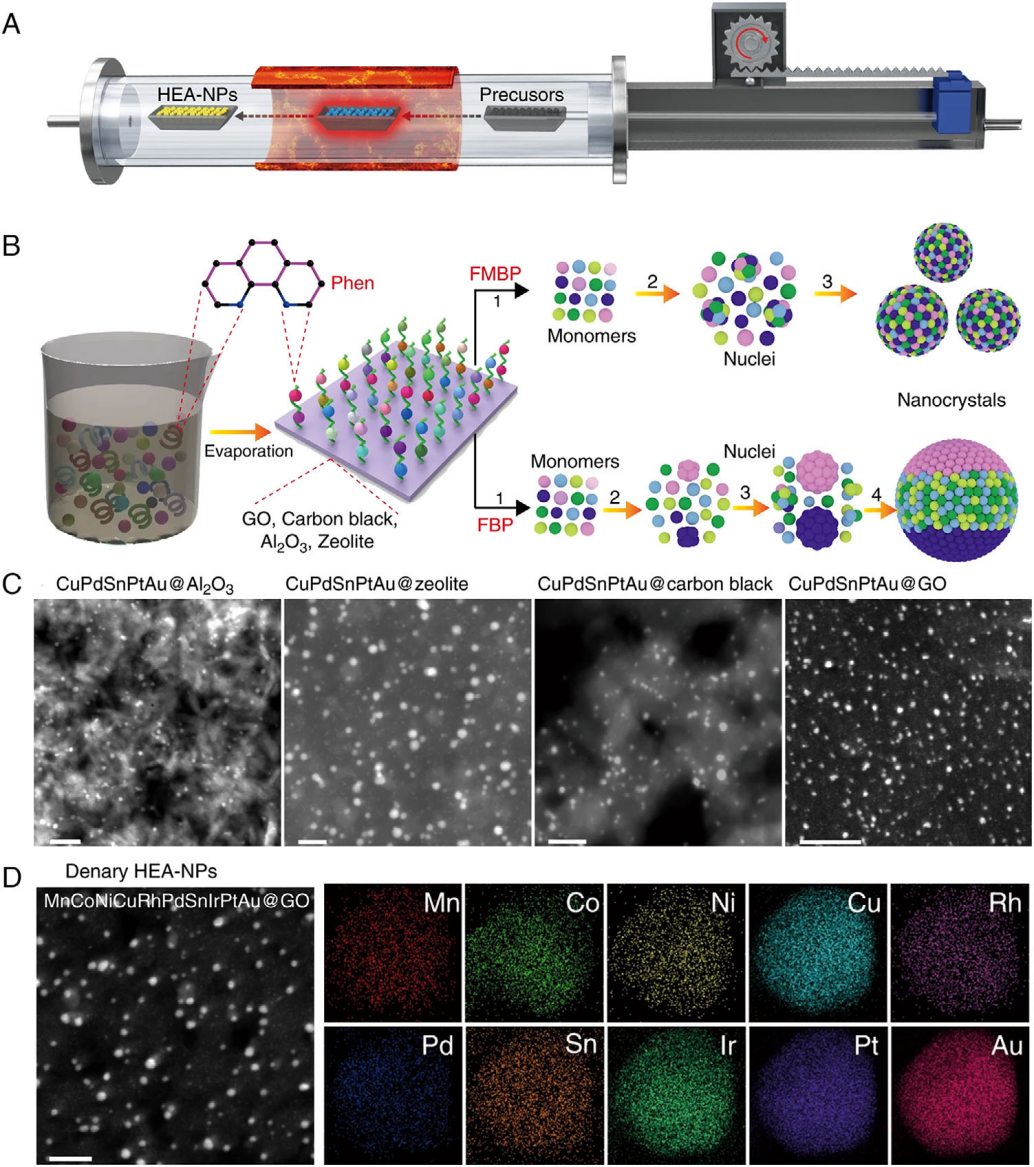
Synthesis and TEM images of HEAs nanoparticles by fast‐moving bed pyrolysis method. (A) Schematic diagram of the FMBP configuration. (B) Schematic diagrams illustrating the synthesis of homogeneous and phase‐separated HEAs NPs via FMBP and FBP strategies, respectively. (C) STEM images of HEAs NPs dispersed on various supports. Scale bar: 20 nm. (D) HAADF‐STEM images of MnCoNiCuRhPdSnIrPtAu@GO and the corresponding elemental maps. Scale bar: 10 nm. Reproduced with permission.^[^ ^42^ ^]^ Copyright 2020, Nature Publishing Group.

In view of the high specific surface area and interfacial effect, the supported nanoparticle catalysts have shown decent catalytic performance. HEAs NPs can be uniformly immobilized on conductive activated carbon fibers by the CTS method. However, the CTS method is difficult to immobilize HEAs NPs on granular supports such as Al_2_O_3_ and zeolite. Alternatively, the fast‐moving bed pyrolysis (FMBP) method has shown the capability of making supported HEAs NPs on versatile substrates (Figure 5A,B).^[^ ^42^ ^]^ The FMBP method realized the simultaneous pyrolysis of metal precursors and the formation of small nucleations by virtue of the fast heating speed. As a proof of concept, quinary (CuPdSnPtAu) HEAs NPs were synthesized by FMBP method on various granular supports such as γ‐Al_2_O_3_, zeolite, carbon black and graphene oxide (GO) (Figure 5C), and denary (MnCoNiCuRhPdSnIrPtAu) HEAs NPs could be synthesized on GO (Figure 5D).

#### 3.1.4 Wet chemical method

**FIGURE 6:**
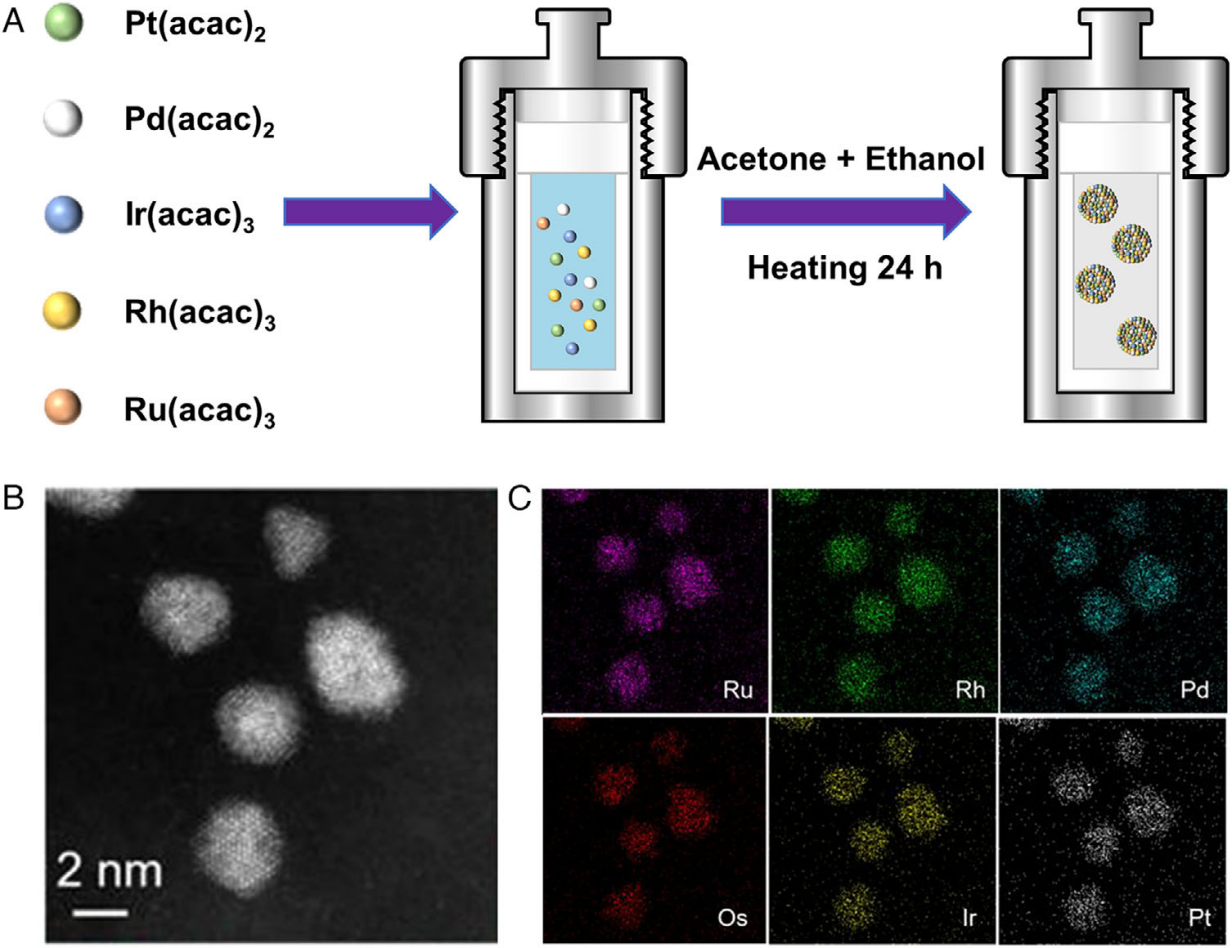
Synthesis and TEM images of HEAs nanoparticles by wet chemical method. (A) Schematic illustration of solvothermal synthesis of HEAs. (B) HAADF‐STEM image of the as‐synthesized RuRhPdOsIrPt and (C) the elemental maps. (B, C) Reproduced with permission.^[^ ^44^ ^]^ Copyright 2020, American Chemical Society.

At present, the predominant wet chemical method to synthesize HEAs is the solvothermal method. The solvothermal reaction occurs in a non‐aqueous solvent at a temperature exceeding the boiling point of the solvent. The high temperature and the high pressure contribute to increasing the multiple metal miscibility. For instance, Bondesgaard et al. obtained quaternary and quinary HEA NPs (Pt_0.25_Pd_0.25_Rh_0.25_Ru_0.25_ and Pt_0.20_Pd_0.20_Ir_0.20_Rh_0.20_Ru_0.20_) with FCC crystal structure via employing noble metal acetylacetone as precursor, and a solvent of acetone‐ethanol mixture in a solvothermal autoclave at 200°C for 24 h (Figure 6A).^[^ ^43^ ^]^ It was found that the utilization of acetylacetone salt plays a pivotal role in the synthesis of HEA NPs, because the robust interaction between metal and acetylacetone reduces the coprecipitation rate. Also by solvothermal method, Wu et al. synthesized HEAs NPs containing six Pt group metals (RuRhPdOsIrPt).^[^ ^44^ ^]^ As depicted in Figure 6B,C, the as‐synthesized Pt group HEA NPs show a narrow size distribution (3.1 ± 0.6 nm).

#### 3.1.5 Ultrasonication‐assisted wet chemical method

**FIGURE 7:**
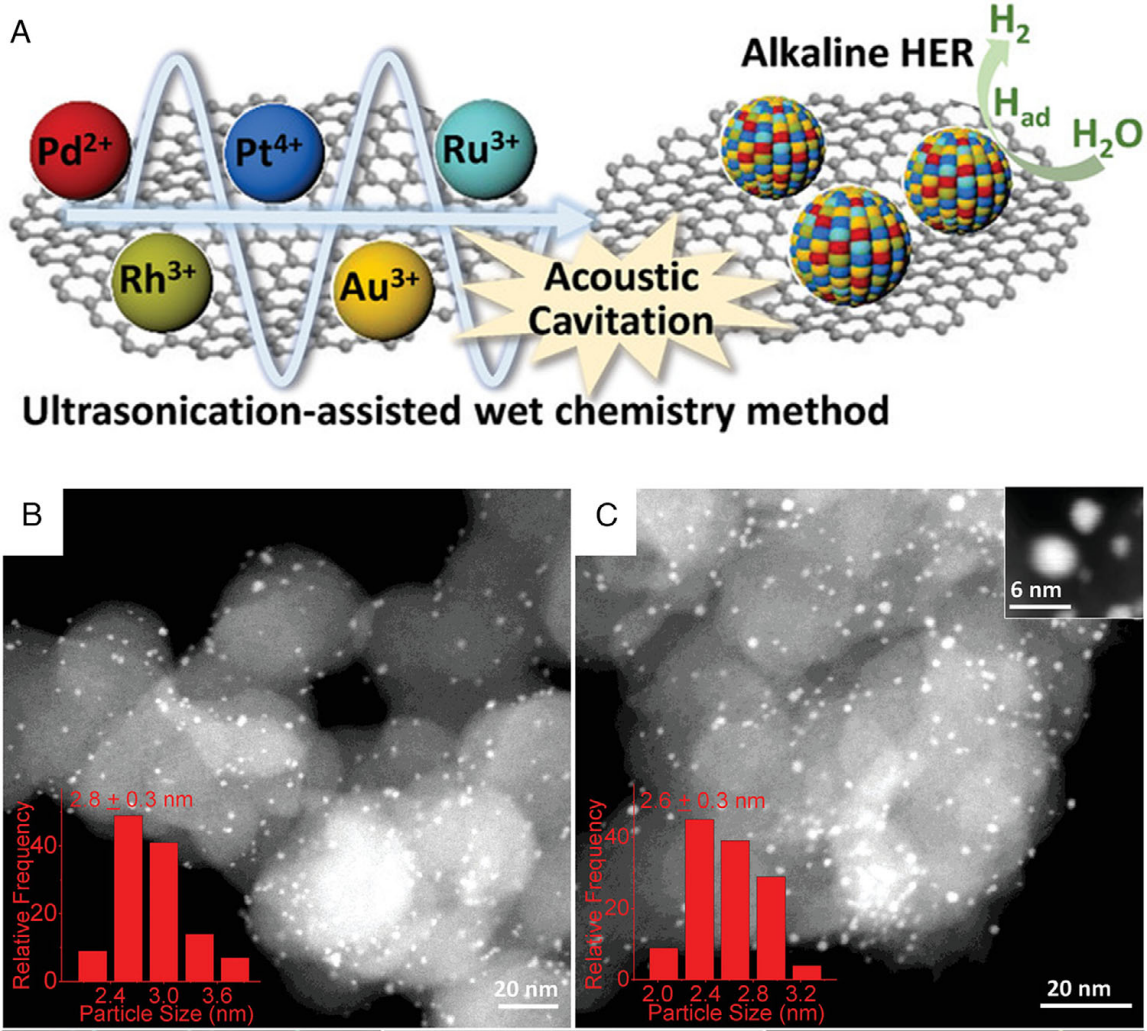
Synthesis and TEM images of HEAs nanoparticles by ultrasonication‐assisted wet chemical method. (A) Schematic representation of the synthesis of PtAuPdRhRu supported on XC‐72 carbon. STEM image of (B) HEA‐NPs/carbon and (C) HEA‐NPs/carbon‐700°C. Reproduced with permission.^[^ ^45^ ^]^ Copyright 2019, Wiley‐VCH.

The formation of alloy NPs is primarily governed by the enthalpy interaction between metals. It has been confirmed that the determining reaction condition for the formation of HEAs NPs is a sharp rate of temperature change, which maximizes the entropy factor governing the ultimate product.^[^ ^41^ ^]^ The phenomenon of acoustic cavitation in the ultrasound process will lead to a rapid increase in temperatures within the local microscopic region, occurring instantaneously and resulting in a sharp temperature ramping rate. This phenomenon can be described as the sequential occurrence of bubble nucleation, progressive expansion, and subsequent collapse at a microscopic scale. The collapse of these bubbles generated a pressure of ≈2000 atm and a temperature of ≈5000°C within a localized microscopic region, occurring within a time frame of ≤10^−9^ s. The instantaneous release of energy can expedite the concurrent reduction of metal ions and promote the growth of the product to the state of entropy maximization. Hence, an ultrasound‐assisted wet chemical method was applied to the synthesis of HEAs NPs (Figure 7A).^[^ ^45^ ^]^ For instance, Liu et al. synthesized PtAuPdRhRu NPs with a size of ≈3 nm by ultrasonic process at room temperature (Figure 7B). Calcination at 700°C under an N_2_ atmosphere further improved the stability and crystal purity of the formed PtAuPdRhRu NPs without changing the size (Figure 7C). In this work, the as‐synthesized HEAs are of two‐phase, which can convert into single‐phase via heat treatment. Liu et al. further conducted an ultrasound‐assisted wet chemical synthesis in alcohol ionic liquid (AIL) as the solvent to enable the formation of single‐phase HEAs.^[^ ^46^ ^]^ Under high‐intensity ultrasound sonication, the co‐reduced metal ions to single‐phase HEA (AuPdPtRhRu) NPs via AIL. The inherent reducing ability of the hydroxyl group in the AIL plays a dominant role in the formation of AuPdPtRhRu single‐phase HEAs.

There are various synthetic methods for HEAs nanoparticles. This review introduces the mechanical alloying method, CTS, FMBP, solvothermal method, and ultrasonic‐assisted wet chemistry method. These methods have their own advantages and disadvantages. The process of the mechanical alloying method is simple, but the purity of the product is difficult to control. The CTS and FMBP methods share the same feature that the metals can be loaded onto the support to ensure uniform distribution of precursors thus increasing the surface area of the catalysts. The support for the FMBP method is diversified but is limited to the carbon matrix for the CTS method, however, the FMBP method is not as fast and convenient as the CTS method. The solvothermal method is the most used method for synthesizing alloys, it can control the particle size and regulate the multidimensional morphology of the alloy through tuning the input amount of metal precursors and reaction conditions. But, compared with other synthetic methods, the solvothermal method is prone to introduce toxic substances, and it is time‐consuming. Ultrasonic‐assisted wet chemistry method utilizes acoustic cavitation, which results in a rapid increase in temperature in local microscopic regions, accelerating the synchronous reduction of the metal ions. However, this method is suitable for HEAs only composed of easily reducible precious metals.

In general, to synthesize HEAs NPs, the reaction conditions involving high energy, high heat, and co‐reduction of metal ions are usually required. Many other methods that can provide such conditions were also reported for synthesizing HEAs NPs, such as the electrospinning method,^[^ ^47^ ^]^ and hydrogen cold plasma.^[^ ^48^ ^]^

### 3.2 The application of HEAs nanoparticles for electrocatalytic reduction reactions

#### 3.2.1 HER

HER is a half‐reaction of electrochemical water splitting and contains multiple catalytic steps.^[^ ^49^ ^]^ The reaction processes for HER in acidic and alkaline environments are different, although they both involve three steps, namely, Volmer step, Heyrovsky step, and Tafel step. In acidic conditions, the Volmer step refers to the catalyst interacting with H^+^ to produce adsorbed hydrogen atoms (H*, * represents adsorption). In the Heyrovsky step, a proton reacts with an electron and a H* to form H_2_. In the Tafel step, two hydrogen atoms adsorbed on the surface of the catalyst combine and evolve into one hydrogen molecule. In alkaline conditions, the Volmer step is that the catalyst interacts with H_2_O and electrons to produce H* and OH^−^.^[^ ^50^ ^]^ In the Heyrovsky step, H* reacts with H_2_O and electrons to form H_2_ and OH^−^. The Tafel step in alkaline media is the same as in acidic media. Based on the additional energy barrier generated by the dissociation of H_2_O, the Volmer reaction is usually considered to be the rate‐determining step for HER. In general, Pt‐based and Pd‐based catalysts exhibit exceptional catalytic activity for the Volmer reaction.^[^ ^51^ ^]^ To reduce the cost, catalysts containing small amounts of active precious metals or solely composed of non‐precious metals are highly pursued.

**FIGURE 8:**
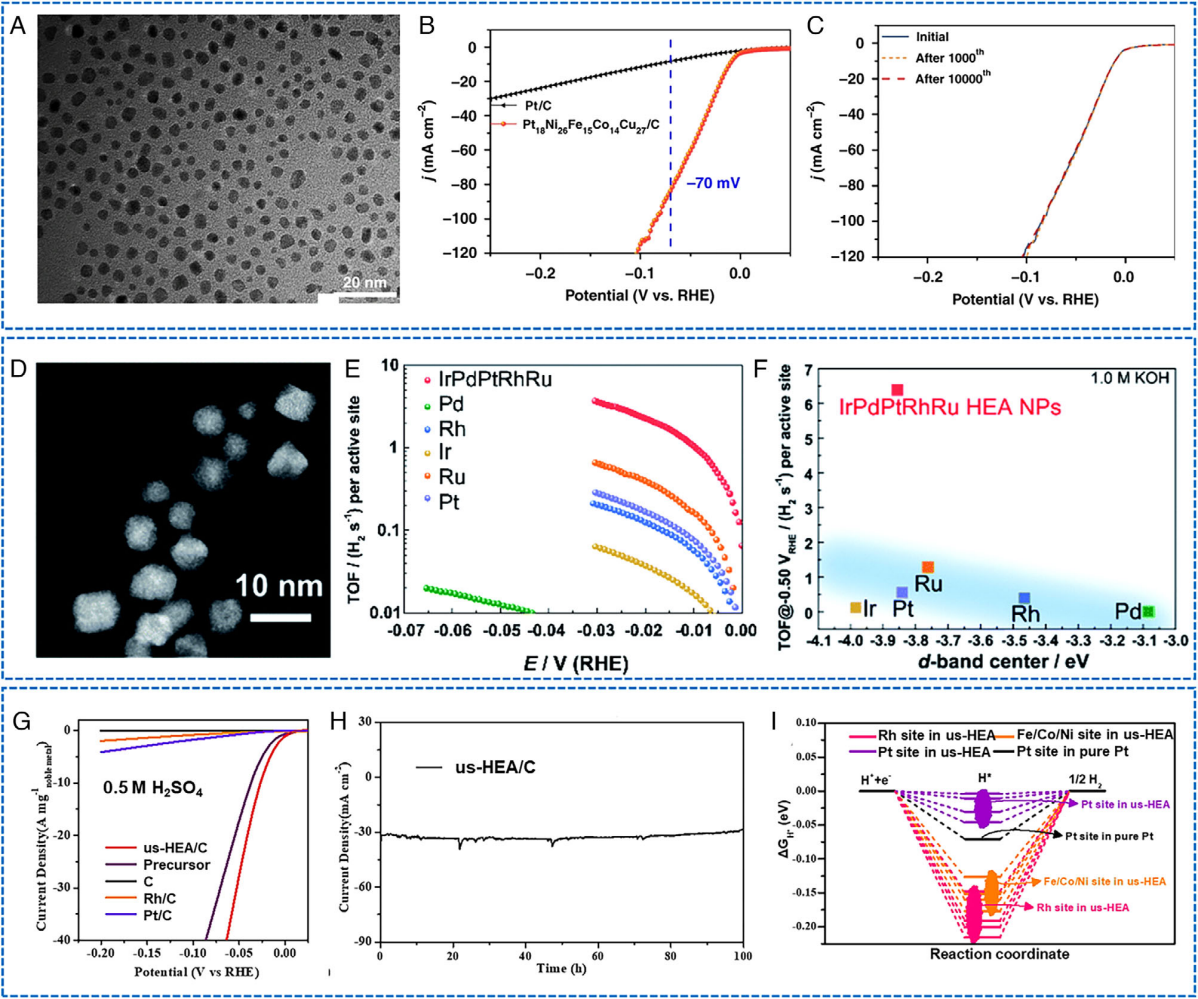
TEM images and electrochemical HER performances of HEAs nanoparticles. (A) TEM images of Pt_18_Ni_26_Fe_15_Co_14_Cu_27_ NPs. (B) HER polarization curves in 1 m KOH. (C) HER LSV for Pt_18_Ni_26_Fe_15_Co_14_Cu_27_/C with different CV cycles. (A–C) Reproduced with permission.^[^ ^52^ ^]^ Copyright 2020, Nature Publishing Group. (D) HAADF‐STEM image of IrPdPtRhRu NPs. (E) TOF in 1.0 m KOH. (F) TOF value at −0.05 V_RHE_ changes with the experimental *d*‐band center. (D–F) Reproduced with permission.^[^ ^54^ ^]^ Copyright 2020, The Royal Society of Chemistry. (G) Mass loading normalized HER polarization curves in 0.5 m H_2_SO_4_. (H) Stability tests of NiCoFePtRh/C. (I) Calculated Δ*G*_H*_ profiles for us‐HEA (111) surface and Pt site on pure Pt (111) surface at monolayer hydrogen coverage (*θ*_H*_ = 1). (G–I) Reproduced with permission.^[^ ^55^ ^]^ Copyright 2021, American Chemical Society (TOF: turnover frequency).

As reported by Li and coworkers, uniform and ultra‐small HEAs Pt_18_Ni_26_Fe_15_Co_14_Cu_27_ NPs were synthesized via a low‐temperature oil‐phase wet chemical method under normal atmospheric pressure.^[^ ^52^ ^]^ The transition metal Ni, Fe, Co, and Cu were chosen because of their high earth‐abundance as well as their comparable atomic radius and low formation heat. Additionally, these elements can readily facilitates the formation of solid solutions with Pt.^[^ ^14^, ^53^ ^]^ As shown in Figure 8A, the size of Pt_18_Ni_26_Fe_15_Co_14_Cu_27_ is consistently measured at about 3.4 nm. The Pt_18_Ni_26_Fe_15_Co_14_Cu_27_/C acting as HER electrocatalyst exhibited a low overpotential of 11 mV at 10 mA cm^−2^ and a specific mass activity of 10.96 A mg^−1^_Pt_ at ‐0.07 V versus RHE in 1 m KOH (Figure 8B). The chronoamperometry and 10,000th CV test results showed that the HEA catalyst has high stability (Figure 8C). Through DFT calculations, the significant electrocatalytic activity and stability of HEA in HER were found to be ascribed to the synergistic effect of effective electron transfer among various constituent elements. The appropriate electronic environment realized the proper adsorption of key intermediates by multiple active sites, as well as enabled effective electron transfer in electrocatalytic process, thereby maximizing the surface electrochemical activity. Wu and coworkers employed hard X‐ray photoelectron spectroscopy (HAXPES) to investigate the electronic structure of HEA NPs consisting of Ru, Rh, Pd, Ir, and Pt (Figure 8D).^[^ ^54^ ^]^ Through HAXPES, the valence band spectrum of HEA NPs was found to be broadened, indicating a random atomic configuration, and consequently leading to diverse local electronic structures. The obtained IrPdPtRhRu NPs have shown excellent HER activity in acidic and alkaline media (33.0/17.0 mV at 10 mA cm_geo_^−2^), and had a higher turnover frequency (TOF) at an overpotential of 25 mV (1.4/3.0 H_2_ per second) (Figure 8E). During the cycle test, IrPdPtRhRu NPs exhibited a higher catalytic stability compared to commercial Pt/C. A correlation analysis between HER activity and *d*‐band center revealed that IrPdPtRhRu NPs with excellent HER activity deviate from the traditional *d*‐band center theory (Figure 8F). This may be contributed to the complexity of the atomic arrangement on the surface of HEA NP, as well as the diversity of local state densities at surface positions.

The uniform dispersion of HEA‐NPs on various supports is crucial for their practical applications. Feng and coworkers synthesized a chain of carbon‐loaded ultra‐small HEA NPs by a co‐reduction method.^[^ ^55^ ^]^ The as‐synthesized NiCoFePtRh NPs were uniformly distributed on the carbon support, exhibiting an average particle size of 1.68 nm, which represents the smallest reported among the HEAs to data. As an electrocatalyst for HER, NiCoFePtRh/C achieved ultra‐high mass activity of 28.3 A ${\mathrm{mg}}_{\mathrm{noble}\;\mathrm{metal}}^{-1}$ at −0.05 V (vs RHE) in acidic solution (Figure 8G). Additionally, NiCoFePtRh/C exhibited an ultra‐high TOF of 30.1 s^−1^ at an overpotential of 50 mV, which is 41.8 times higher compared to Pt/C catalyst. Furthermore, it exhibited excellent stability and did not degrade even after a duration of 100 h (Figure 8H). Theoretical calculations showed that the enhanced HER activity of NiCoFePtRh/C was mainly attributed to the active sites provided by Rh and Pt, the tunable electronic structure of Pt/Rh, and the synergistic effects between the five elements (Figure 8I). Similarly, Gao and coworkers used an FMBP method to ensure the fast simultaneous pyrolysis of mixed metal precursors at high temperatures.^[^ ^42^ ^]^ The as‐synthesized quinary FeCoPdIrPt NPs had a high HER stability (150 h) and a high mass activity (9.1 mA µg 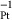) at an overpotential of 100 mV. Feng and coworkers used an anchoring and alloying strategy to uniformly incorporate CoNiCuMgZn onto an ultra‐thin 2D porous graphene conductive support.^[^ ^56^ ^]^ During the growth of HEA, GO served as both template and reducing agent. The adjustment of the original input amount of the metal source promoted the precise control of CoNiCuMgZn particle size within the range of 40–200 nm. Thanks to the excellent electronic conductivity of graphene and the synergistic effect combine with the abundant catalytic sites of CoNiCuMgZn NPs, the obtained materials exhibited a significant catalytic activity and an excellent stability for more than 100 h for HER. Moreover, the homogeneous distribution of CoNiCuMgZn NPs provided a plethora of exposed electrochemical active sites for HER. DFT calculations showed that the distinctive embedding structure of CoNiCuMgZn on graphene facilitates H* adsorption and enhances reaction kinetics.

#### 3.2.2 ORR

ORR is a key reaction that affects the performance of diverse electrochemical energy conversion and storage devices, including fuel cells and metal‐air batteries. However, ORR is a complicated and sluggish process that involves the transfer of four electrons and four protons.^[^ ^57^ ^]^ There are two types of ORR reaction pathways (4e^−^ ORR to H_2_O/OH^−^ or 2e^−^ ORR to H_2_O_2_). The ORR involves three possible reaction mechanisms, which are dissociation mechanism, binding mechanism, and peroxide mechanism. For the dissociation mechanism, the O─O bond of O_2_ is broken at the adsorption site to form two *O, which are subsequently reduced to 2*OH, and the 2*OH are then reduced to form H_2_O. For the binding mechanism, the O_2_ fixed at the adsorption site is activated to form *OOH, which is decomposed into *O and *OH, and then reacts with protons and electrons to form H_2_O. For the peroxide mechanism, H^+^/e^−^ pair combines with *OOH to generate HOOH*, producing H_2_O_2_. Moreover, HOOH* can also be decomposed into *OH, which then reacts to generate H_2_O according to the dissociation mechanism. In conclusion, there are abundant intermediates (*O, *OH, *OOH, HOOH*) during the ORR reaction process, making the reaction system relatively complicated.^[^ ^58^, ^59^ ^]^ The reasonable design of ORR electrocatalysts will play an important role in optimizing the adsorption/desorption of intermediates.^[^ ^60^ ^]^

**FIGURE 9:**
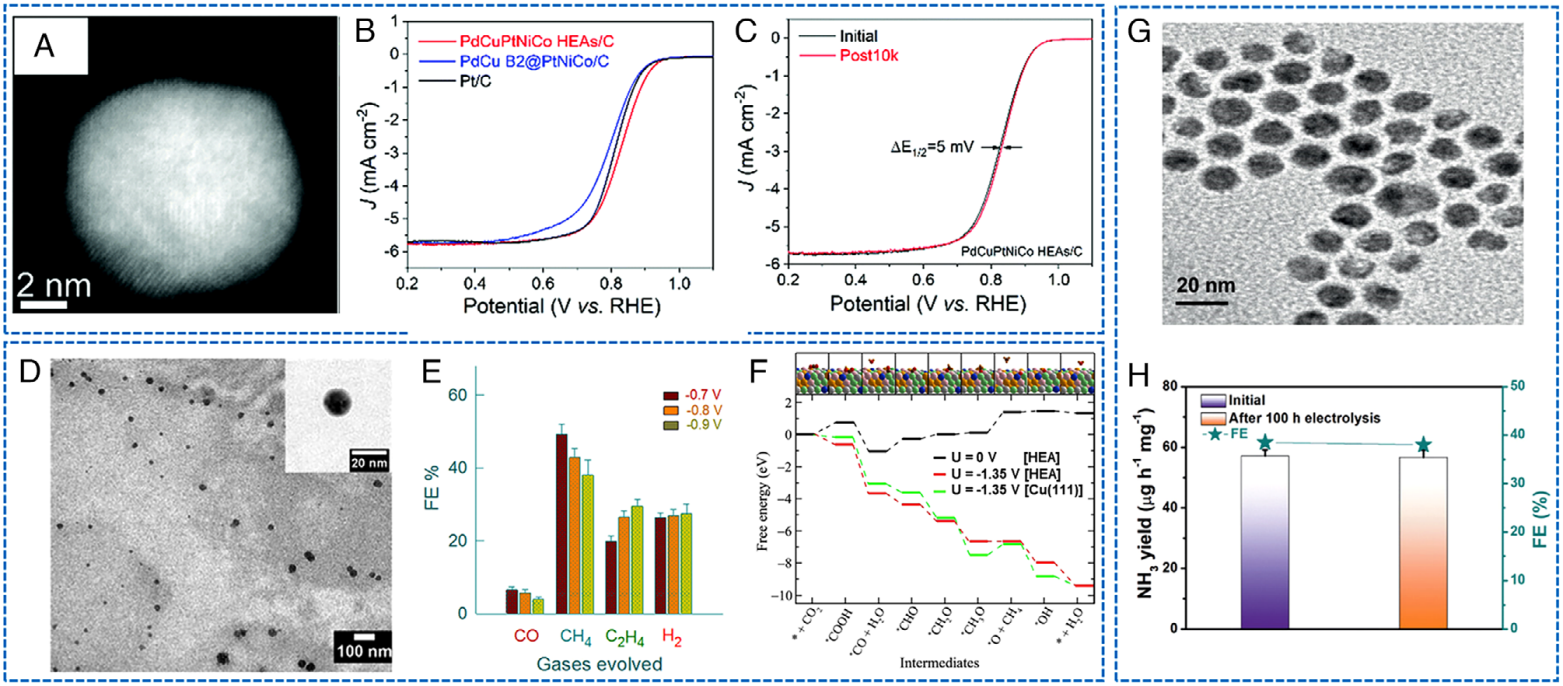
TEM images and electrochemical ORR, CO_2_RR, and NRR performances of HEAs nanoparticles. (A) The STEM image of PdCuPtNiCo/C. (B) ORR LSV curves of PdCuPtNiCo/C and (C) polarization curves before and after 10,000 cycles. (A–C) Reproduced with permission.^[^ ^61^ ^]^ Copyright 2021, The Royal Society of Chemistry. (D) TEM image of AuAgPtPdCu NPs (inset image: a single NP). (E) Bar diagram for the FE of their respective products. (F) Free‐energy diagram of CO_2_RR and optimized structures of the intermediates on the AuAgPtPdCu surface. (D–F) Reproduced with permission.^[^ ^62^ ^]^ Copyright 2020, American Chemical Society. (G) TEM image of RuFeCoNiCu NPs. (H) NH_3_ yields and FEs after conducting at 0.05 V versus RHE for 1 and 100 h. (G, H) Reproduced with permission.^[^ ^63^ ^]^ Copyright 2020, Wiley‐VCH (FE: Faradaic efficiency).

Chen et al. reported a seed‐mediated co‐reduction strategy (SMCR) to synthesize HEAs NPs.^[^ ^61^ ^]^ Specifically, Pt, Ni, and Co were co‐deposition on the PdCu NP as seeds through SMCR, and core@shell PdCuB2@PtNiCo was converted into PdCuPtNiCo NPs with a particle size of 10.6 ± 0.2 nm (Figure 9A). PdCuPtNiCo/C has shown a positive onset potential (*E_onset_*) of 1.06 V and a half‐wave potential (*E*_1/2_) of 0.83 V (Figure 9B). The PdCuPtNiCo/C had an outstanding mass activity of 176.1 mA ${\mathrm{mg}}_{\mathrm{Pt}}^{-1}$ by normalizing the mass of Pt. The PdCuPtNiCo/C also had the largest kinetic current density in the potential range of 0.75–0.95 V. The stability test of PdCuPtNiCo/C showed that *E*_1/2_ increased by 5 mV after 10,000 cycles (Figure 9C). The superior ORR activity observed in PdCuPtNiCo/C can be related to the high dispersion of Pt/Pd content and the low elemental diffusivity of HEAs.

Short‐range order has been observed in bulk HEAs.^[^ ^64^ ^]^ Zhu et al. employed ligand‐assisted interface assembly in combination with NH_3_ annealing to synthesize ordered HEA (OHEA, FeCoNiCuPd) NPs on a novel 2D nitrogen‐rich mesoporous carbon sandwich framework (OHEA‐MNC).^[^ ^65^ ^]^ OHEA‐mNC had an ultra‐thin 2D nanosheet structure with a large mesoporous diameter of about 10 nm, and FeCoNiCuPd NPs were evenly distributed without obvious aggregation. The OHEA‐mNC catalyst exhibited much higher ORR activity (a large *E*_1/2_ of 0.90 V) and higher stability (only 0.01 V attenuation after 10,000 cycles) than both the disordered catalyst and Pt/C catalyst. The experimental results and theoretical calculations demonstrated that the spatial structural design, coupled with the existence of ordered HEA phase, effectively facilitated mass transfer, and modulate active sites for the adsorption and electron transfer of reactants in the ORR process.

#### 3.2.3 CO_2_RR

Electrocatalytic CO_2_RR can alleviate the greenhouse effect by converting CO_2_ into high‐value‐added chemicals.^[^ ^66^ ^]^ CO_2_RR involves multiple pathways and is a complex reaction with up to 12 electron transfers. Electrocatalytic CO_2_ reduction mainly includes CO_2_ activation, surface reaction, and product desorption processes. The initial step of CO_2_RR is that CO_2_ is adsorbed on the active site of the catalyst, and single‐electron reduction occurs to form CO_2_*^−^ intermediates. Due to that CO_2_ is a thermodynamically stable molecule, the formation of CO_2_*^−^ requires overcoming a large potential energy barrier, thus activation is usually considered to be the rate‐determining step for CO_2_RR.

The surface reactions and product desorption processes of CO_2_RR can be analyzed through the reaction pathways of 2‐electron and multi‐electron mechanisms. In the case of the 2‐electron reaction, one of the products is CO. CO_2_ undergoes a proton‐coupled electron transfer step to form COOH*. Alternatively, CO_2_ can combine with the catalyst involving an electron transfer step, followed by protonation to form COOH*. The COOH* species then undergoes a proton‐coupled electron transfer process to generate CO*, which is subsequently released from the catalyst surface. Another product that can be formed is formic acid or formate. CO_2_ combines with a proton to form HCOO*, which then acquires protons and electrons to form HCOO^−^. For multi‐electron reactions, there are two main pathways. Firstly, the CO* intermediates undergo multi‐step proton‐electron transfers to yield C_1_ products such as methane, methanol, and formaldehyde. Secondly, CO*, being the most crucial intermediate, participates in C─C coupling reactions on the catalyst surface. The coupling of C─C bonds represents a key step in the formation of C_2+_ products.

The CO_2_RR involves the cleavage of C─O bonds, the coupling of C─C bonds, and the formation of C─H bonds, leading to the formation of different products.^[^ ^67^ ^]^ However, achieving a high selectivity of chemical products of CO_2_RR is challenging, especially when competing with HER in a water environment.^[^ ^68^ ^]^ This requires the optimization of CO_2_RR catalysts to achieve high efficiency, selectivity, and stability.

HEAs have become a unique platform for exploring the synergistic mechanism between different atoms for CO_2_RR due to their single solid solution phase and tunable stoichiometric number. The CO and H adsorption energies for all surface sites on disordered CoCuGaNiZn and AgAuCuPdPt HEAs (111) surfaces were calculated by Pedersen et al., who combined DFT with machine learning.^[^ ^30^ ^]^ By doing so, a method that is probabilistically unbiased for the discovery of highly active and selective HEAs catalysts for CO_2_RR was proposed. This method allows the optimization of HEAs compositions, increasing the possibility of weak hydrogen adsorption sites to restrain molecular hydrogen formation and strong CO adsorption sites to promote CO reduction. Subsequent experiments reported by Nellaiappan et al. verified the feasibility of using machine learning to find the optimal combination of HEAs.^[^ ^62^ ^]^ In this work, HEAs: AuAgPtPdCu synthesized by melting and cryogrinding have been developed as “single‐atom catalysts”, where Cu atoms are fixed by other metals within the “FCC face” crystal structure. These NPs exhibited an average size of 16 ± 10 nm (Figure 9D). The synergistic effect of Cu in combination with all other metals played a pivotal role in enhancing stability. By using the multi‐metal HEA method, a Faraday efficiency (FE) of gas products reached about 100% at a lower potential (−0.3 V vs RHE) (Figure 9E). DFT method was used to compare HEAs with the original Cu (111) surface, the reasons behind the catalytic activity and selectivity of HEAs for CO_2_RR were attributed to the fact that the adsorption trend of ─*OCH_3_ and *O among the eight intermediates on both the surface of Cu (111) and HEA has been reversed, which is conducive to CO_2_RR (Figure 9F).

#### 3.2.4 NRR

Ammonia (NH_3_) is one of the highly valuable chemical feedstock and an essential component in industrial applications.^[^ ^69^ ^]^ It plays a significant role in industrial and agricultural production because it is widely used in the synthesis of drugs and fertilizers.^[^ ^70^ ^]^ Electrocatalytic NRR (N_2_(g) + 6H^+^ +6e^−^ → 2NH_3_(g)) is a carbon‐free method for nitrogen (N_2_) fixation into NH_3_, which is helpful for mitigating energy‐intensive and environmentally polluting challenges associated with the industrial Haber–Bosch method. In general, electrochemical N_2_ to NH_3_ conversion occurs via two main mechanisms, which are dissociative mechanism and associative mechanism. According to the dissociative mechanism, the N≡N triple bond of adsorbed N_2_ would first split to form two N atoms adsorbed on the surface of catalysts. Then the adsorbed N atoms undergo reduction reactions to produce the NH_3_. According to the associative mechanism, the adsorbed N_2_ molecule is first reduced to form N_2_H*_x_* *, and then the N─N bond breaks and the reduction reactions occur to form NH_3_.^[^ ^59^ ^]^ However, due to the extremely high bond‐breaking energy of N_2_, poor water solubility, and competitive HER in the electrocatalytic process, the catalytic effect still does not meet the industrial requirements.^[^ ^71^ ^]^ Therefore, the design and preparation of advanced electrocatalysts are crucial for achieving satisfactory catalytic performance.

Zhang et al. reported RuFeCoNiCu NPs with small size (about 16 nm) synthesized by oil phase synthesis under normal pressure and low‐temperature conditions (≤250°C) (Figure 9G).^[^ ^63^ ^]^ In 0.1 m KOH, the NH_3_ yield was 57.1 µg h^−1^ ${\mathrm{mg}}_{\mathrm{cat}}^{-1}$ at 0.05 V versus RHE, and the corresponding FE reached 38.5%. In addition, it also exhibited attractive electrochemical stability. After conducting 100 h of testing, a marginal decline in activity was observed (Figure 9H). Theoretical calculations showed that Fe surrounded by alloy metals represents the optimal position for N_2_ adsorption and activation. Co─Cu and Ni─Ru showed remarkable surface hydrogenation ability at a low overpotential, forming *H on their surfaces. This H source is prone to triggering the activation of N_2_ adsorbed at neighboring Fe sites, resulting in the production of NH_3_. Moreover, Yu et al. studied the NRR catalytic performance of FeCoNiCuPd HEAs via DFT calculations.^[^ ^72^ ^]^ The calculation showed the correlation between structural stability and composition ratio. By evaluating the initial N_2_ adsorption energy of Ni_0.3_(FeCoCuPd)_0.175_ and assessing the free energy of intermediates in various pathways, the exposed crystal plane (111) of Ni_0.3_(FeCoCuPd)_0.175_ displayed excellent NRR activity with a minimum overpotential of 0.34 eV. The bridge site of Fe─Co (b‐Fe─Co) on the surface represented an optimal position for the adsorption and activation of N_2_. It was found that variations in metal ratios within HEAs affect the *d*‐band center, leading to differences in catalytic activity.

### 3.3 The application of HEAs nanoparticles for electrocatalytic oxidation reactions

#### 3.3.1 OER

OER is a highly demanding reaction process involving the transfer of four‐electron and four‐proton.^[^ ^3^ ^]^ For acidic OER, an H_2_O molecule dissociates into OH* and H*. The formed OH* subsequently undergoes a deprotonation reaction to form O*. Then, the O* binds with a second H_2_O molecule to form the OOH* intermediate. Finally, OOH* undergoes a deprotonation reaction to form O_2_*, which desorbs from the catalytic sites to form O_2_. For alkaline OER, the OH^−^ adsorbs on the catalyst surface and forms OH*. The formed OH* reacts with OH^−^ to form O*. Then, the O* binds with OH^−^ to form the OOH*. The OOH* binds with OH^−^ to form O_2_*, which desorbs from the catalytic sites to form O_2_.^[^ ^59^, ^73^ ^]^ The OER is kinetically sluggish, and it is essential to develop OER catalysts.

**FIGURE 10:**
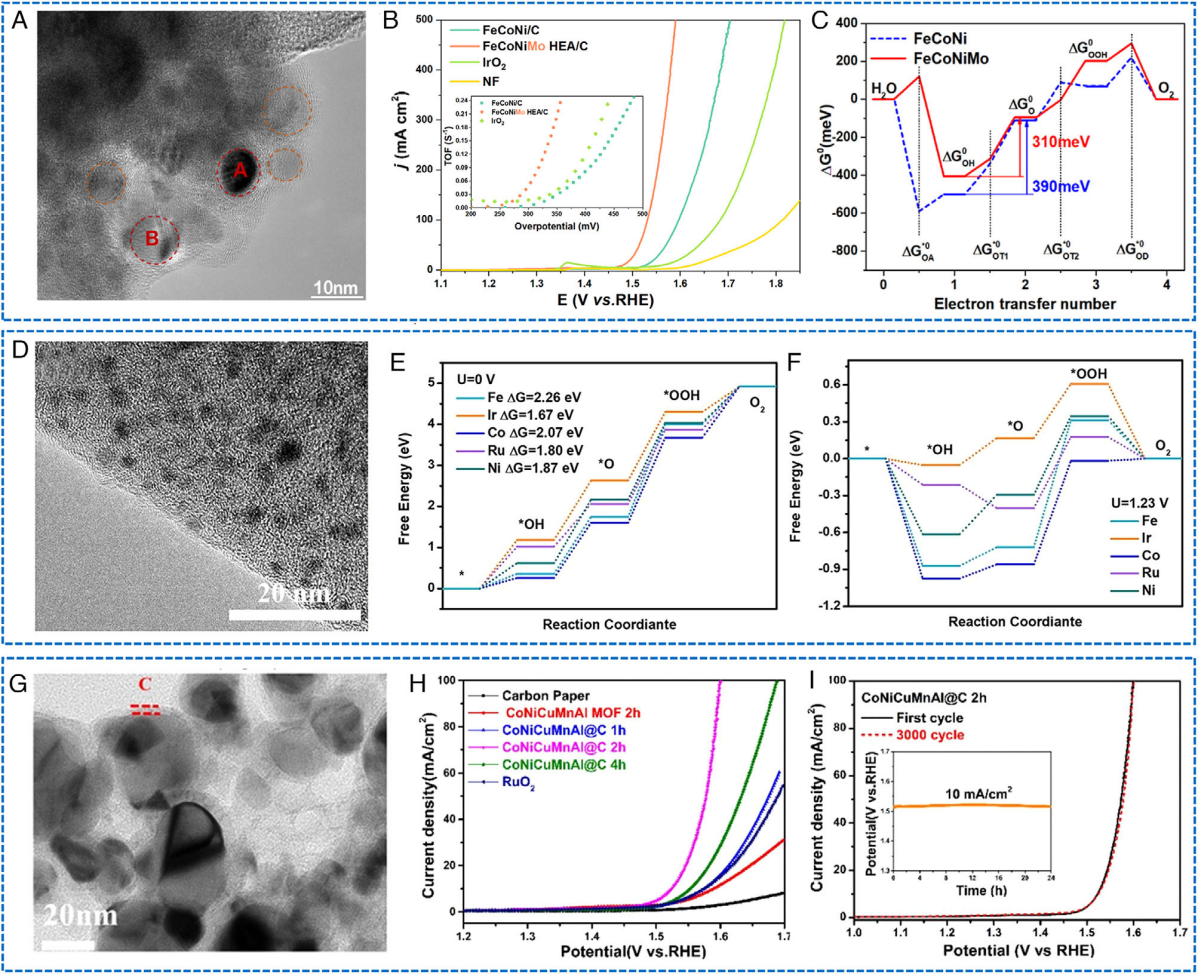
TEM images and electrochemical OER performances of HEAs nanoparticles. (A) HRTEM image of FeCoNiMo/C. (B) IR‐corrected OER polarization curves (inset: TOF plots) and (C) free energy diagram of the FeCoNi/C and FeCoNiMo/C catalysts. (A–C) Reproduced with permission.^[^ ^74^ ^]^ Copyright 2022, American Chemical Society. (D) TEM image of FeCoNiIrRu/CNFs. (E, F) The corresponding free energy diagrams for OER of FeCoNiIrRu surfaces under *U* = 0 and 1.23 V. (D–F) Reproduced with permission.^[^ ^47^ ^]^ Copyright 2021, Elsevier. (G) TEM image of CoNiCuMnAl@C 2 h. (H) LSV curves. (I) Stability test of CoNiCuMnAl@C 2 h. (G–I) Reproduced with permission.^[^ ^75^ ^]^ Copyright 2021, Elsevier.

Some HEAs NP electrocatalysts have shown decent OER activity and stability. For instance, Mei et al. reported a solvothermal method to synthesize HEA catalysts with Mo coordination (the fourth transition metal Mo was incorporated into the FeCoNi‐based alloys, Figure 10A).^[^ ^74^ ^]^ The FeCoNiMo catalyst exhibited a current density of 10 mA cm^−2^ at an impressively low overpotential of only 250 mV in alkaline media (Figure 10B). Importantly, FeCoNiMo exhibited a superior OER stability, maintaining a high current density of 100 mA cm^−2^ for over 60 h. Through physical characterization and computational simulation, it was found that the transfer of Mo electrons to Fe, Co, and Ni species in the FeCoNiMo catalyst. This electron transfer weakened the OH* bond, thereby enhancing the OER activity (Figure 10C). Meanwhile, OER kinetic simulation verified that the presence of Mo coordination in FeCoNi can accelerate the rate‐determining deprotonation step of OH* during OER. Zhu et al. utilized Fe, Co, Ni, and Ru as structurally stable elements while employing nano‐scale HEA as a substrate to stabilize Ir site.^[^ ^47^ ^]^ Ultrasmall FeCoNiIrRu NPs were synthesized in situ on carbon nanofibers (CNFs), showing thermodynamic‐induced phase evolution (Figure 10D). FeCoNiIrRu/CNFs exhibited an overpotential of 241 mV at 10 mA cm^−2^, and demonstrated remarkable durability, with a mass activity of 205 mA ${\mathrm{mg}}_{\mathrm{Ir}+\mathrm{Ru}}^{-1}$ in acidic media. The high stability was attributed to the fact that the sluggish diffusion effect of HEA, which effectively restrains the leaching and dissolution of Ir metals. Theoretical calculations indicated that the electron density in FeCoNiIrRu NPs undergoes redistribution, with a transfer from the low electronegative elements (Fe, Co, Ni) to high electronegative elements (Ir, Ru) (Figure 10E,F). This redistribution enhanced the reactivity of Ir and facilitated the transformation of *OOH as well as the formation of O_2_. In addition, Wang et al. presented a scalable method for synthesizing CoNiCuMnAl/C NPs derived from the polymetallic metal–organic framework (MOF).^[^ ^75^ ^]^ FCC HEA was coated with an ultra‐thin carbon shell (Figure 10G), which may be the reason for the long‐term stability for OER (almost no decrease at 200 mA cm^−2^ for 30 h). The optimized catalyst promoted alkaline OER with an overpotential of 215 mV at 10 mA cm^−2^ and demonstrated excellent stability (Figure 10H,I). In conjunction with DFT calculations and comparative experiments, it has been elucidated that Ni/Co‐OOH accelerates the rate‐determining step (O* → OOH*) during the OER process.

#### 3.3.2 AOR

Direct alcohol fuel cells are favored in various renewable energy technologies due to their high energy conversion efficiency, convenient transportation and storage, as well as environmental friendliness.^[^ ^76^ ^]^ In general, AOR is a process involving multiple steps of adsorption and dissociation. Taking the methanol oxidation reaction as an example. During the reaction, methanol is adsorbed on the catalyst surface to form CH_3_OH*. Then, after the activation of the C─H bond, the CH_3_OH* is oxidized to form CO* on the surface of the catalyst. CO* interacts with the adjacent OH* to form CO_2_, and OH* is formed by the dissociation of H_2_O molecules.

Promoting the oxidation of ─OH, accelerating the C─C bond cleavage (complete oxidation pathway), and enhancing the resistance to CO poisoning are the primary considerations in the AOR process.^[^ ^77^ ^]^ However, commercialization is significantly limited by the scarcity of active and stable anode electrocatalysts.^[^ ^78^ ^]^ Pt, which has excellent performance in MOR and EOR, still has many shortcomings for AOR, including limited tolerance towards CO intermediate, high cost, low utilization rate, and poor stability.^[^ ^79^ ^]^

**FIGURE 11:**
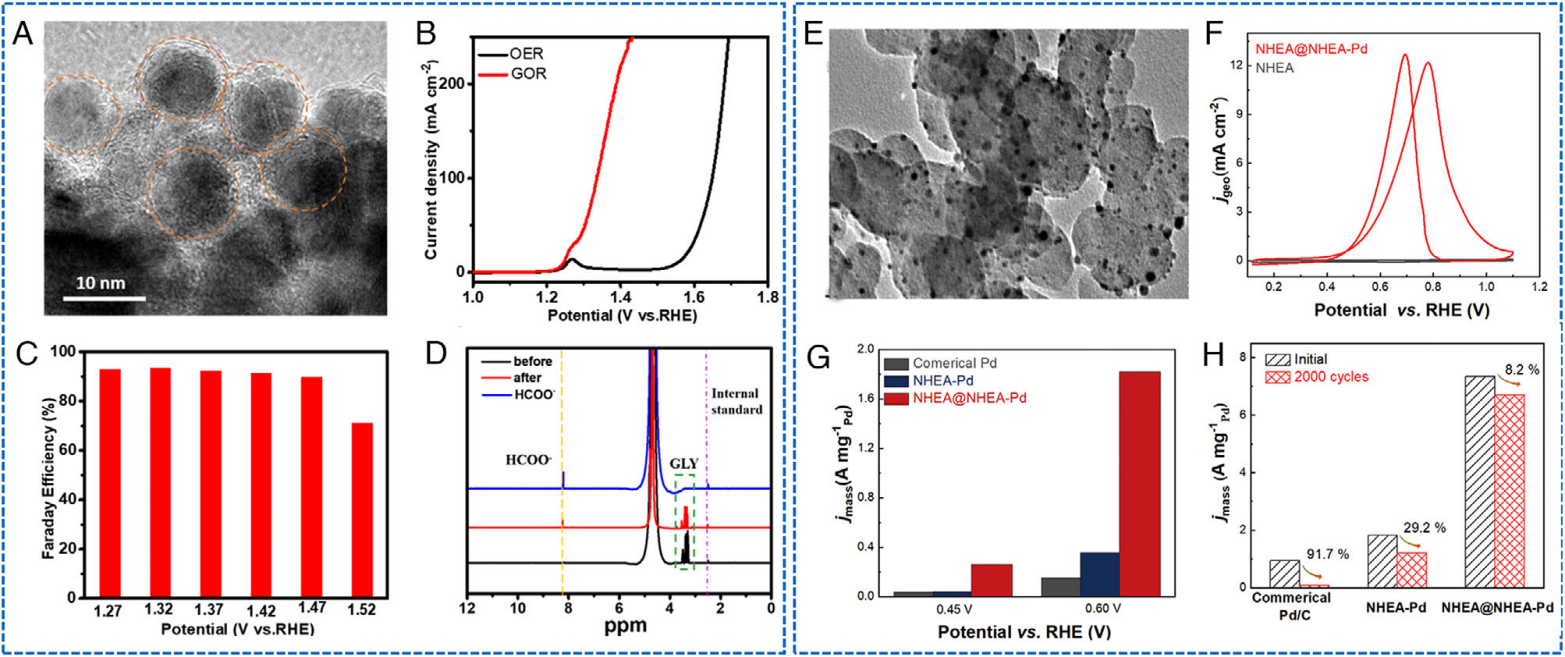
TEM images and electrochemical AOR performances of HEAs nanoparticles. (A) HRTEM image of the CoNiCuMnMo NPs. (B) LSV curves of CoNiCuMnMo‐NPs/CC in 1 m KOH with and without 0.1 m glycerol addition. (C) FEs for formate production at varied potentials. (D) ^1^H NMR measurements of GOR of glycerol (green) to formate (yellow). (A–D) Reproduced with permission.^[^ ^80^ ^]^ Copyright 2022, American Chemical Society. (E) TEM image of NHEA@NHEA‐Pd. (F) Geometric activities of NHEA@NHEA‐Pd in N_2_‐saturated aqueous solution containing 1.0 m KOH and 1.0 m ethanol. (G) *j_mass_* values at 0.45 and 0.60 V in the forward scan. (H) Comparison of the stability test. (E–H) Reproduced with permission.^[^ ^81^ ^]^ Copyright 2022, Wiley‐VCH.

The AOR process involves multiple steps, for which the abundant metal active sites of HEAs could be useful. Fan and coworkers synthesized a self‐supported electrode with uniform HEAs NPs (about 10 nm) on a carbon cloth (Figure 11A).^[^ ^80^ ^]^ Electrochemical studies confirmed the good performance of CoNiCuMnMo in the electrocatalytic glycerol oxidation reaction (GOR) at a low potential (1.25 V vs RHE to reach 10 mA cm^−2^). Moreover, it exhibited a remarkable selectivity of exceeding 90% over a wide potential range of 1.27–1.47 V versus RHE to produce formate (Figure 11B–D). The surface atomic configuration of CoNiCuMnMo was investigated using a Monte Carlo simulation based on machine learning. And it was pointed out that the catalytic activity center is the Mo site. Wu and coworkers synthesized PtCrTaVFeAl NPs with a diameter of about 10 nm by taking advantage of the strong reducing ability of hydrogen anions in hydrogen‐cooled plasma, which can simultaneously reduce Pt and other metal precursors.^[^ ^48^ ^]^ Pt_28_Cr_20_Ta_20_V_11_Fe_11_Al_10_/C presented a maximum MOR current density of 2.82 mA ${\mathrm{cm}}_{\mathrm{Pt}}^{-2}$ at an operating voltage of 0.85 V.

The fine control of the surface of HEAs is challenging. In response to this challenge, Zeng and coworkers reported a surface‐modified HEA catalyst with a non‐precious metal HEA core (FeCoNiSn, denoted as NHEA) and a Pd alloy as the shell to form NHEA@NHEA‐Pd (Figure 11E).^[^ ^81^ ^]^ When applied to EOR, NHEA@NHEA‐Pd demonstrated a remarkable catalytic activity of 7.34 A ${\mathrm{mg}}_{\mathrm{Pd}}^{-1}$, exceptional stability with a retention rate of 91.8% after 2000 cycles, and improved tolerance towards CO (Figure 11F–H). This design emphasizes the importance of selectively exposing Pd‐based active sites for EOR. Moreover, it is necessary to coordinate high‐entropy and control morphology to improve the activity and stability of HEA catalysts.

The contribution of HEAs nanoparticles to electrocatalytic reduction reactions and oxidation reactions is demonstrated from the following three aspects. (i) Size effect. Reducing the size of the nanoparticles can increase the exposed active sites and surface area of the catalyst. (ii) Regulation of structure and electronic environment. The selective exposure of specific active sites and the improvement of the multi‐element coordinated electronic environment can achieve appropriate adsorption of the key intermediates of the reaction. (iii) The synergistic effect and sluggish diffusion effect. The synergistic effect can maximize the physical and chemical characteristics of each component in the alloy, thus to increase the electrocatalytic activity. The sluggish diffusion effect can effectively prevent the leaching of elements, greatly improving the stability of the catalyst.

In summary, the application of HEAs NPs in the field of electrocatalysis has been extensively studied. The material composition, crystal structure, preparation method, and performances in various electrocatalytic reactions are summarized in Table 1. Although HEAs NPs have made significant progress in electrocatalysis, further research is still needed to develop and design multi‐dimensional HEAs and to optimize the surfaces of the catalysts for high electrocatalytic performance.

**TABLE 1:** The summary of structure, synthetic methods, and electrocatalytic performance of HEAs nanoparticles.

					Overpotential from CV/LSV curve				
Morphology	Materials	Structure	Synthetic method	Catalytic reaction	Overpotential @ scan rate	Current density	Tafel slope	Electrolytes	Ref.
Nanoparticles	PtNiFeCoCu (3.4 ± 0.6 nm)	FCC	Low‐temperature oil phase strategy	HER	11 mV@20 mV s^−1^	10 mA cm^−2^	30 mV dec^−1^	1.0 m KOH	[52]
	IrPdPtRhRu (5.5 ± 1.2 nm)	FCC	One‐pot polyol process	HER	33 mV@5 mV s^−1^ 17 mV@5 mV s^−1^	10 mA ${\mathrm{cm}}_{\mathrm{geo}}^{-2}$ 10 mA ${\mathrm{cm}}_{\mathrm{geo}}^{-2}$	‐ ‐	0.5 m H_2_SO_4_ 1.0 m KOH	[54]
	FeCoPdIrPt (2 nm)	FCC	Fast‐moving bed pyrolysis	HER	42 mV@5 mV s^−1^	10 mA cm^−2^	82 mV dec^−1^	1.0 m KOH	[42]
	CoNiCuMgZn (40 nm)	FCC	Polyol method	HER	158 mV@1 mV s^−1^	10 mA cm^−2^	36.1 mV dec^−1^	1.0 m KOH	[56]
	IrPdPtRhRu (5 nm)	FCC	Plasma ionic liquid reduction	HER	60 mV@5 mV s^−1^	10 mA cm^−2^	42 mV dec^−1^	1.0 m KOH	[82]
	PtAuPdRhRu (≈3 nm)	FCC	Ultrasonication‐assisted wet chemistry method	HER	−0.19 V@5 mV s^−1^	30 mA cm^−2^	62 mV dec^−1^	1.0 m KOH	[45]
	FeCoNiCuPtIr (≈10 nm)	FCC	Rapid laser impact method	HER	21 mV@5 mV s^−1^	10 mA cm^−2^	54.5 mV dec^−1^	1.0 m KOH	[83]
	IrPdPtRhRu (1.32 ± 0.41 nm)	FCC	Continuous‐flow reactor with a liquid‐phase reduction method	HER	6 mV@5 mV s^−1^	10 mA cm^−2^	‐	1 m HClO_4_	[84]
	NiCoFePtRh (1.68 nm)	FCC	Chemical co‐reduction method	HER	27 mV	10 mA cm^−2^	30.1 mV dec^−1^	0.5 m H_2_SO_4_	[55]
	TiNbTaCrMo (13 nm)	FCC	Confinement‐assisted arc and plasma shock	HER	0.97 V versus RHR@10 mV s^−1^	50 mA cm^−2^	96.33 mV dec^−1^	Natural seawater	[85]
	CoFeNiPtTa (≈1.8 nm)	FCC	Shear‐assisted liquid metal surface reduction	HER	10.6 mV@5 mV s^−1^	10 mA cm^−2^	37 mV dec^−1^	0.5 m H_2_SO_4_	[86]
	FeCoNiCuPtIr (≈10 nm)	FCC	Rapid laser impact method	OER	255 mV@5 mV s^−1^	10 mA cm^−2^	61.7 mV dec^−1^	1.0 m KOH	[83]
	FeCoNiMo (8 ± 0.3 nm)	FCC	hydrothermal method	OER	250 mV@1 mV s^−1^	10 mA cm^−2^	48.02 mV dec^−1^	1.0 m KOH	[74]
	FeCoNiIrRu	FCC	The electrospinning + chemical vapor deposition	OER	241 mV@5 mV s^−1^	10 mA cm^−2^	153 mV dec^−1^	0.5 m H_2_SO_4_	[47]
	CoNiCuMnAl	FCC	Straightforward precipitation route + pyrolysis	OER	215 mV@5 mV s^−1^	10 mA cm^−2^	35.6 mV dec^−1^	1.0 m KOH	[75]
	CoFeNiPtTa (≈1.8 nm)	FCC	Shear‐assisted liquid metal surface reduction	OER	290 mV@20 mV s^−1^	10 mA cm^−2^	35 mV dec^−1^	1.0 m KOH	[86]
	FeCoNiMnCu (40 nm)	FCC	Cathodic plasma electrolysis deposition	OER	280 mV@10 mV s^−1^	10 mA cm^−2^	59 mV dec^−1^	1.0 m KOH	[87]
	Fe_0.5_CoNiCuZn_0.8_	FCC	One‐step electrolytic reduction	OER	340 mV@5 mV s^−1^	10 mA cm^−2^	48 mV dec^−1^	1.0 m KOH	[88]
	FeCoNiCuIr (16–32 nm)	FCC	One‐step heat‐up method	OER	360 mV@5 mV s^−1^	10 mA cm^−2^	70.1 mV dec^−1^	1.0 m KOH	[89]

					E_1/2_ from CV/LSV curve				
	Materials	Structure	Synthetic method	Catalytic reaction	E_1/2_ @ scan rate	Current density	Tafel slope	Electrolytes	Ref.
	FeCoNiCuPd (10 nm)	FCC	Wet chemical method	ORR	0.90 V versus RHE@10 mV/s	≈5.7 mA cm^−2^	55 mV dec^−1^	0.1 m KOH	[65]
	PtPdFeCoNi (12 ± 4 nm)	FCC	High‐temperature injection	ORR	0.920 V versus RHE@20 mV s^−1^	1.80 A ${\mathrm{cm}}_{\mathrm{Pt}}^{-2}$	‐	0.1 m HClO_4_	[90]
	FeCoNiMnCr	FCC	Soaking + pyrolysis	ORR	≈800 mV versus RHE@5 mV s^−1^	*J_peak_* ≈ 4 mA cm^−2^	60 and 122 mV dec^−1^	0.1 m KOH	[91]
	PdCuPtNiCo (10.6 ± 0.2 nm)	FCC	Via seed‐mediated co‐reduction	ORR	0.83 V versus RHE@10 mV s^−1^	≈5.7 mA cm^−2^	‐	0.1 m KOH	[61]

					**Potential from chronoamperometric response**				
	Materials	Structure	Synthetic method	Catalytic reaction	Potential @ scan rate	Current density	Faradic efficiency	Electrolytes	Ref.
	AuAgPtPdCu (16 ± 10 nm)	FCC	Melting and cryogrinding	CO_2_RR	−0.9 V versus Ag/AgCl	−13.81 mA cm^−2^	100%	CO_2_‐saturated K_2_SO_4_	[62]

					**Potential from CV curve**				
	Materials	Structure	Synthetic method	Catalytic reaction	Potential @ scan rate	Current density	Selectivity	Electrolytes	Ref.
	PtNiFeCoCu (≈3.4 nm)	FCC	One‐pot oil phase synthesis method	MOR	0.82 V versus RHE@20 mV s^−1^	114.93 mA cm^−2^	‐	1 m KOH + 1 m CH_3_OH	[52]
	CoNiCuMnMo (10 nm)	FCC	Hydrothermal + pyrolysis reduction	GOR	1.25 V versus RHE@2 mV s^−1^	10 mA cm^−2^	92% (FE_formate_)	1.0 m KOH + 0.1 m C_3_H_8_O_3_	[80]
	PtCtTaVFeAl (10 nm)	FCC	Hydrogen cold plasma‐enabled synthesis	MOR	0.85 V versus RHE@50 mV s^−1^	2.82 mA ${\mathrm{cm}}_{\mathrm{Pt}}^{-2}$	‐	0.1 m HClO_4_ + 0.5 m CH_3_OH	[48]
	FeCoNiSn @ FeCoNiSn‐Pd (25 nm)	FCC	Carbon thermal shock	EOR	0.69 V versus RHE@20 mV s^−1^	12.05 mA cm^−2^	‐	1.0 m KOH + 1.0 m C_2_H_5_OH	[81]
	PtFeCoNiCu (2.37 nm)	FCC	Impregnation reduction process	MOR	0.68 V versus SCE@50 mV s^−1^	3.29 mA ${\mathrm{cm}}_{\mathrm{Pt}}^{-2}$	‐	0.1 m HClO_4_ + 1.0 m CH_3_OH	[92]

					Potential from LSV curve				
	Materials	Structure	Synthetic method	Catalytic reaction	Potential @ scan rate	NH_3_ yield	NH_3_ Selectivity	Electrolytes	Ref.
	RuFeCoNiCu (16 nm)	HCP	Solvothermal	NRR	0.05 V versus RHE	57.1 μg h^–1^ ${\mathrm{mg}}_{\mathrm{cat}}^{-1}$	38.5%	0.1 m KOH	[63]

“‐” represents not mentioned in the references.

## 4 SYNTHESIS AND APPLICATIONS OF 1D HEAS NANOWIRES FOR ELECTROCATALYSIS

The agglomeration and dissolution of metal nanoparticle catalysts in the reaction process will result in a fast deterioration in catalytic performance.^[^ ^93^ ^]^ It is worth noting that HEAs have garnered significant attention in the realm of catalysis by virtue of their adjustable composition and high stability in corrosive environments.^[^ ^94^, ^95^ ^]^ Furthermore, accurate control of the material structure at a level of nanoscale is a potent approach for adjusting the properties of the catalyst. 1D nanomaterials are a series of high‐performance electrocatalysts because they expose numerous active sites, a large specific surface area, and impressive resistance to solubility.^[^ ^96^ ^]^ By harnessing the structural advantages of 1D NWs alongside the multi‐component characteristics of HEAs, it is possible to not only regulate reaction kinetics but also to enhance selectivity and stability. Therefore, the precise manipulation of the composition, morphology, and size of 1D HEAs becomes intriguing in electrocatalysis.

### 4.1 The synthetic methods of HEAs nanowires

**FIGURE 12:**
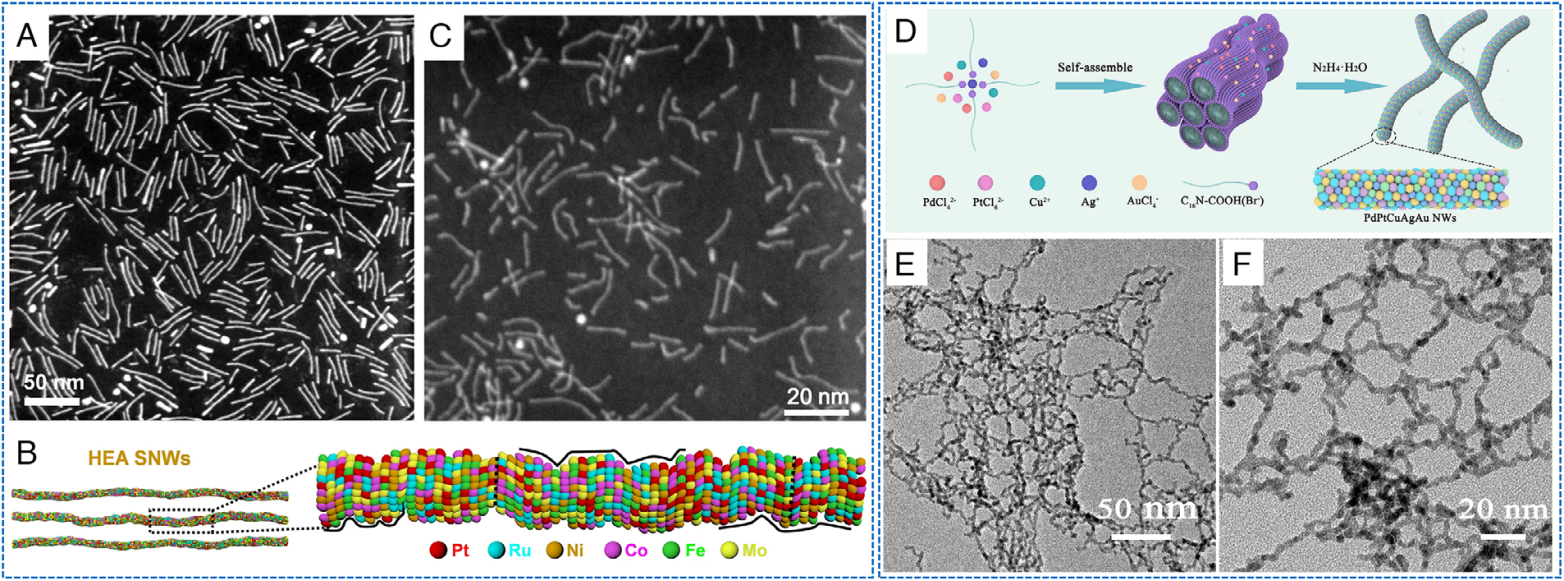
Synthesis and TEM images of HEAs nanowires. (A) The HAADF‐STEM image of NiCoFeMo sub‐nanowires. (B) 3D models and enlarged atomic model of NiCoFeMo NWs. (A, B) Reproduced with permission.^[^ ^94^ ^]^ Copyright 2021, Nature Publishing Group. (C) HAADF‐STEM image of PtRuRhCoNi NWs. (C) Reproduced with permission.^[^ ^97^ ^]^ Copyright 2022, Elsevier. (D) Schematic explanation for the formation process of PdPtCuAgAu NWs. (E, F) Low‐magnification of PdPtCuAgAu NWs. (D–F) Reproduced with permission.^[^ ^98^ ^]^ Copyright 2022, Elsevier.

The synthesis of HEAs nanowires is mainly based on the wet chemical method. The key point in the synthesis of HEAs NWs instead of NPs is the addition of a structure‐directing agent during the synthesis. Zhan et al. used acetylacetone metal salts, hexacarbonyl molybdenum (Mo(CO)_6_) as metal precursors, oleylamine as solvent, stearyl trimethylammonium bromide (STAB) as structure‐guiding agent, and glucose as reducing agent to synthesize PtRuNiCoFeMo sub‐nanowires (the synthesized HEA NWs with a mean diameter of 1.8  ±  0.3 nm, Figure 12A).^[^ ^94^ ^]^ The material had a high density of surface atomic steps and facet boundaries (Figure 12B). Similarly, Li et al. designed a novel ultra‐thin PtRuRhCoNi NWs (average diameter of about 1.6 nm) by a solvothermal method (Figure 12C).^[^ ^97^ ^]^ The synthesis of PtRuRhCoNi NWs used acetylacetone metal salts as metal precursors, Mo(CO)_6_ as a reducing agent, oleylamine as a solvent, and surfactant cetyltrimethylammonium bromide (CTAB) as structure‐guiding agent. The formation mechanism of PtRuRhCoNi NWs was explored by studying the morphology and composition changes in samples at different reaction times. As the reaction time increased, the nanowires gradually became longer. The morphology and atomic ratio of PtRuRhCoNi NWs remained unaltered after a 2‐h reaction, which proves the complete formation of NWs. Interestingly, when CTAB was absent during the synthesis process, the product was small NPs rather than NWs. While, without adding Mo(CO)_6_, the generated NPs were large, irregular, and aggregated. The observed phenomena indicated that the morphological evolution process of PtRuRhCoNi NWs is guided by CTAB as a structure template for guiding NWs growth, while Mo(CO)_6_ acts as a reducing agent to regulate the reaction rate. Fan et al. presented a facile one‐pot solution‐phase method for synthesizing PdPtCuAgAu NWs networks using carboxyl‐functionalized surfactants as soft templates (Figure 12D).^[^ ^98^ ^]^ The PdPtCuAgAu NWs possessed an average diameter of ≈3.5 nm and a length in the range of several hundred nanometers (Figure 12E,F). In specific, by employing C_16_N‐COOH (Br^−^) molecule as a template, metal chloride as a metal precursor, and N_2_H_4_·H_2_O as a reducing agent, the synthesis of PdPtCuAgAu NWs was achieved through a one‐pot liquid‐phase method. The formation process involved the initial self‐assembly of C_16_N‐COOH (Br^−^) molecules into rod‐like micelles, followed by their interaction with the metal precursor via electrostatic forces to form an inorganic–organic hybrid structure. After the addition of N_2_H_4_·H_2_O, the metal precursor underwent rapid reduction to alloy in the micelle structure owing to the nano‐constraint effect. During the material synthesis process, the reduction time only took a few minutes at a fixed reaction temperature of 95°C. In summary, it can be found that the synthesis of HEAs NWs by wet chemical method requires both the addition of a structure‐directing agent and acceleration of the reduction rate.

Based on the reported synthetic methods of HEAs, it can be found that solvothermal method is currently the main method for synthesizing multi‐dimensional HEAs. The nanowires, the nanosheets/nanoplates, the convex cubic HEAs, the hollow sphere HEAs, and the mesoporous sphere HEAs can all be synthesized by solvothermal method. The similarity lies in the addition of structure‐directing agents in the synthesis of nanowires, convex cubic HEAs, hollow sphere HEAs, and mesoporous sphere HEAs. The formation mechanism shows that the material tends to be particles in the absence of a structure‐directing agent. The formation of nanosheets/nanoplates utilizes different binding properties of metals with different reducing agents. For instance, when Mo(CO)_6_ is used as a reducing agent, CO generated in situ can effectively prevent the growth of Pd (111) along the [111] direction, thus enabling the formation of a nanosheet.

### 4.2 The application of HEAs nanowires for electrocatalytic reduction reaction

**FIGURE 13:**
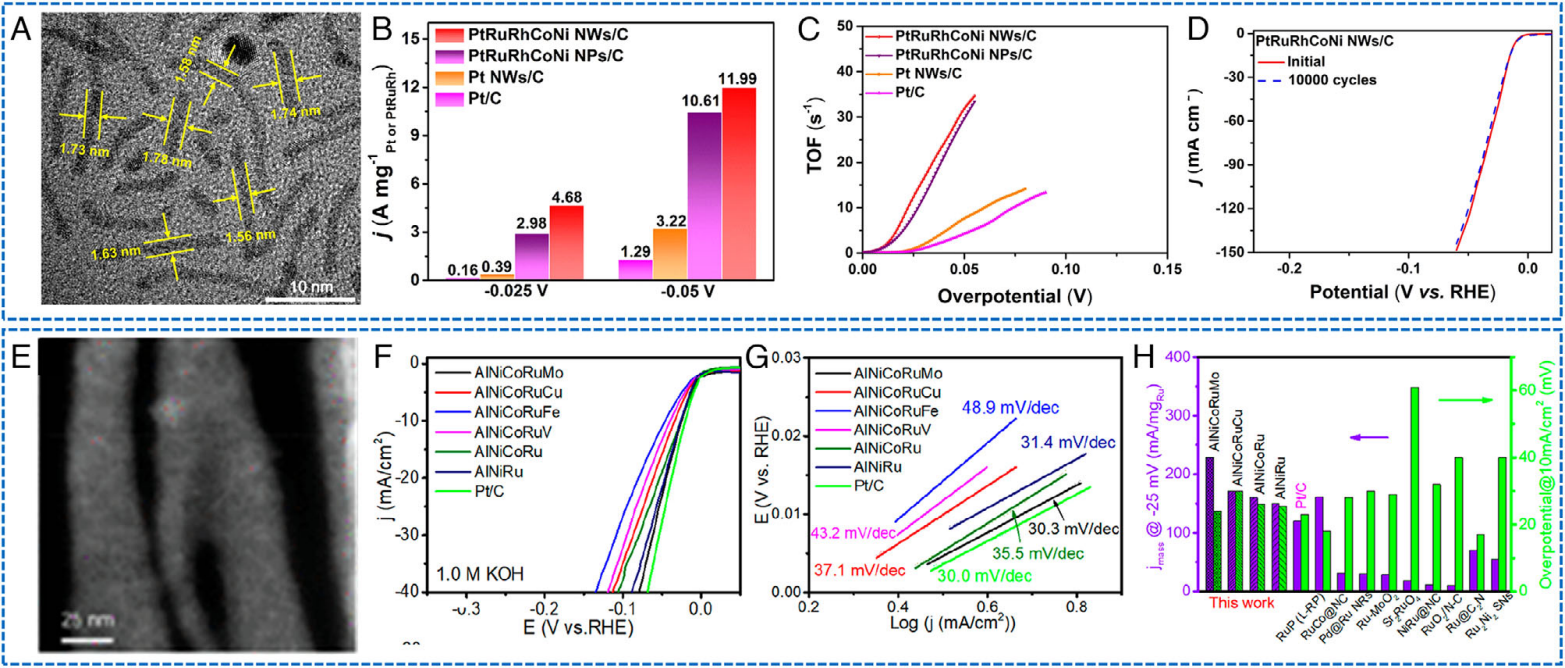
TEM images and electrochemical HER performances of HEAs nanowires. (A) The TEM image of PtRuRhCoNi NWs. (B) Comparisons of HER mass specific activities at different potentials in 0.5 m H_2_SO_4_ solution. (C) TOF. (D) 10,000 CV cycles test of PtRuRhCoNi NWs/C. (A–D) Reproduced with permission.^[^ ^97^ ^]^ Copyright 2022, Elsevier. (E) SEM images of the AlNiCoRuMo NWs. (F) HER LSV. (G) The corresponding Tafel curves, and (H) comparison of Ru mass activity and overpotential with literature data. (E–H) Reproduced with permission.^[^ ^99^ ^]^ Copyright 2020, American Chemical Society (TOF: turnover frequency).

HEAs NWs have been used in HER applications. Li et al. designed a novel ultra‐thin PtRuRhCoNi NWs (average diameter of about 1.6 nm) by a solvothermal method (Figure 13A).^[^ ^97^ ^]^ In this work, PtRuRhCoNi NWs exhibited excellent electrocatalytic performance. For HER, PtRuRhCoNi NWs/C achieved a superior mass activity (11.99 A ${\mathrm{mg}}_{\mathrm{PtRuRh}}^{-1}$ in 0.5 m H_2_SO_4_ and 8.07 A ${\mathrm{mg}}_{\mathrm{PtRuRh}}^{-1}$ in an alkaline medium at −0.05 V vs RHE), TOF (31.9 s^−1^ in 0.5 m H_2_SO_4_ and 26.7 s^−1^ in 1 m KOH at −0.05 V vs RHE) and excellent stability (200 h in 0.5 m H_2_SO_4_ and 1 m KOH, respectively) (Figure 13B–D). Jin et al. introduced a general dealloying procedure for the synthesis of a series of pre‐designed robust HEA NWs, including Al–Ni–Co–Ru–X, where X = Mo, Cu, V, Fe, as multifunctional electrocatalysts (Figure 13E).^[^ ^99^ ^]^ Specifically, the etched Al–Ni–Co–Ru–Mo NWs exhibited a remarkable electrocatalytic activity for HER. The catalyst required a small overpotential of −24.5 mV to achieve 10 mA cm^−2^, and the Tafel slope was 30.3 mV dec^−1^ (Figure 13F–H). During the continuous HER process at −0.025 V, the five‐membered AlNiCoRuMo exhibited stable catalytic performance, with an activity retention rate of about 95.2% after the 100‐h test. Compared with NPs, these mechanically and chemically robust HEAs NWs not only significantly reduce the content of precious metals but also effectively improve the flexibility of their electronic structure, thereby exhibiting a wide range of catalytic abilities.

### 4.3 The application of HEAs nanowires for electrocatalytic oxidation reaction

**FIGURE 14:**
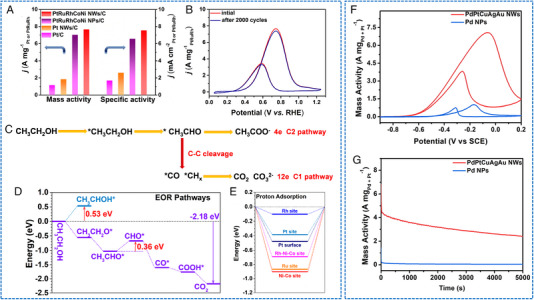
Electrochemical AOR performances of HEAs nanowires. (A) Mass specific activities. (B) CV curves before and after 2000 cycles of PtRuRhCoNi NWs/C in 1 m KOH + 1 m C_2_H_5_OH. (C) Scheme of active EOR pathways. (D) The energy change of EOR. (E) The binding energy comparisons of the proton. (A–E) Reproduced with permission.^[^ ^97^ ^]^ Copyright 2022, Elsevier. (F) CV curves and (G) *i*–*t* chronoamperometry curves of PdPtCuAgAu NWs and Pd NPs collected in 1.0 m KOH and 1.0 m ethylene glycol. (F, G) Reproduced with permission.^[^ ^98^ ^]^ Copyright 2022, Elsevier.

HEAs NWs have also been used in AOR applications. Li et al. synthesized ultra‐thin PtRuRhCoNi NWs as multifunctional electrocatalysts.^[^ ^97^ ^]^ This catalyst showed excellent EOR, MOR activity, and long‐term durability, which may be ascribed to the fact that HEA optimizes the electronic structure through a robust self‐complementary effect. For AOR, the EOR activity of PtRuRhCoNi NWs/C in alkaline solution was 7.68 A ${\mathrm{mg}}_{\mathrm{PtRuRh}}^{-1}$ (9.50 A ${\mathrm{mg}}_{\mathrm{Pt}}^{-1}$) and 78% C_1_ selectivity, and the MOR activity was 6.65 A ${\mathrm{mg}}_{\mathrm{PtRuRh}}^{-1}$ (8.20 A ${\mathrm{mg}}_{\mathrm{Pt}}^{-1}$) (Figure 14A,B). Moreover, PtRuRhCoNi NWs/C also exhibited significant EOR activity in acidic environments. Due to the multiple dehydrogenation and oxidation of ethanol, the reaction path of EOR becomes intricate. Through in situ FTIR spectroscopy analysis of PtRuRhCoNi NWs/C and commercial Pt/C during EOR, revealing that PtRuRhCoNi NWs/C exhibited accelerated reaction kinetics at a low potential. These phenomena indicated that the C_1_ pathway facilitates the cleavage of the C─C bond, leading to CO_2_ formation on PtRuRhCoNi NWs/C (Figure 14C). In addition, theoretical calculations demonstrated that the good electrochemical activity of HEA benefits from the self‐complementary effect of robust orbital coupling, thereby maximizing the electrocatalytic reaction and the preferential binding of key intermediates (Figure 14D,E). Furthermore, Fan et al. reported a one‐pot liquid‐phase method for preparing high‐entropy PdPtCuAgAu nanowire networks utilizing carboxyl‐functionalized surfactants as soft templates.^[^ ^98^ ^]^ Benefiting from the advantages of synergistic composition (such as high entropy effect, sluggish diffusion effect, and lattice distortion effect) and structure (anisotropy and fine nanowires), the PdPtCuAgAu alloy electrocatalyst exhibited the remarkable improvement of electrocatalytic performance for EOR. This includes exceptional activity with superior mass activity (7.7 A ${\mathrm{mg}}_{\mathrm{Pd}+\mathrm{Pt}}^{-1}$), excellent stability/durability, resistance to poisoning effect, as well as favorable electrocatalytic kinetics. Moreover, the PdPtCuAgAu NWs showed excellent electrocatalytic performance in ethylene glycol oxidation (Figure 14F,G).

The outstanding performance of HEAs NWs for electrocatalytic reactions can be attributed to the fine nanowire structure and the flexibility of their electronic structure. Nanowires have advantages related to their anisotropy, unique structure, and surface properties. Nanowires also have fewer lattice boundaries, fewer surface defects, and facilitated electron and mass transfer, resulting in better electrocatalytic activity than other morphologies. For instance, the Pt‐based HEAs NWs have lower vulnerability to dissolution, Ostwald ripening, and aggregation than the Pt‐based HEAs NPs, thus having improved stability for the oxidation reactions.

## 5 SYNTHESIS AND APPLICATIONS OF 2D HEAS NANOSHEETS/NANOPLATES FOR ELECTROCATALYSIS

Shaping materials into 2D morphology can greatly enhance the performance on account of the high surface area and specific crystal plane. Graphite can be easily shaped into 2D morphology due to their weak interlayer Vander Waals forces. However, the synthesis of 2D nanosheets or nanoplates of alloys poses a huge challenge.^[^ ^100^ ^]^ Herein, we will discuss the synthetic methodology of 2D HEAs and their applications in electrocatalysis.

### 5.1 The synthetic methods of HEAs nanosheets/nanoplates

**FIGURE 15:**
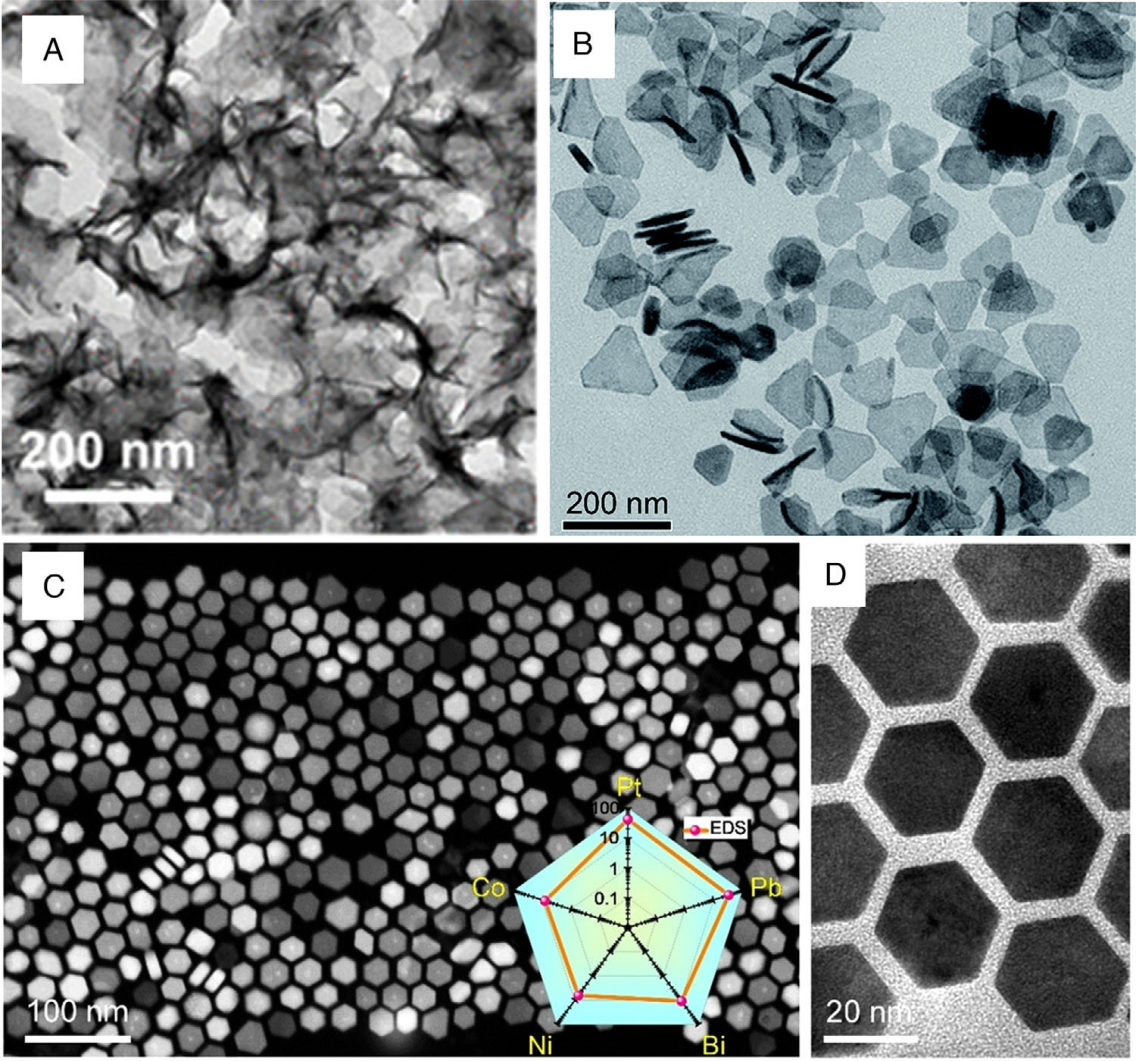
The TEM images of HEAs nanosheets/nanoplates. (A) The TEM image of PdMoGaInNi nanosheets. Reproduced with permission.^[^ ^101^ ^]^ Copyright 2022, American Chemical Society. (B) TEM of PtAgBiCo nanoplates. Reproduced with permission.^[^ ^102^ ^]^ Copyright 2017, The Royal Society of Chemistry. (C) HAADF‐STEM image. (D) TEM image of PtBiPbNiCo HPs. (C, D) Reproduced with permission.^[^ ^103^ ^]^ Copyright 2022, Wiley‐VCH.

Wet chemical methods have generally been applied to synthesizing HEAs nanosheets/nanoplates. Fu et al. synthesized wrinkled PdMoGaInNi nanosheets with a thickness of approximately 1.6 nm via the solvothermal method (Figure 15A).^[^ ^101^ ^]^ The metal precursors were blended with oleylamine, and the excessive Mo(CO)_6_ was used as a reducing agent, while the in‐situ generated CO was used as a capping agent. It has been reported that the strong adsorption of CO on the surface of Pd (111) prevented its growth along the [111] direction, which is the reason for the formation of nanosheet morphology. This is the first example of HEAs with a 2D morphology. Wang et al. synthesized multi‐metal triangular PtAgBiCo nanoplates by a two‐step solvothermal method. This work has shown that the introduction of Co can change the crystallinity of PtAgBiCo, resulting in the formation of highly efficient triangular nanoplates with a form yield exceeding 90% (Figure 15B).^[^ ^102^ ^]^ Zhan et al. synthesized medium/high entropy composite core/shell PtBiPbNiCo hexagonal nanoplates (HEA HPs) by a solvothermal method.^[^ ^103^ ^]^ Specifically, acetylacetone metal salts and basic carbonates were utilized as metal precursors, while L‐ascorbic acid served as the reducing agent. Oleylamine and 1‐octadecene were employed as solvents. Structural analysis showed that the average diameter of these uniform HPs was 26.2 ± 3.3 nm, and the average thickness was 8.4 ± 1.5 nm. The HEA‐HP comprised a PtBiPb medium entropy core and a PtBiNiCo high entropy shell (Figure 15C,D).

### 5.2 The application of HEAs nanosheets/nanoplates for electrocatalytic reduction reactions

#### 5.2.1 HER

**FIGURE 16:**
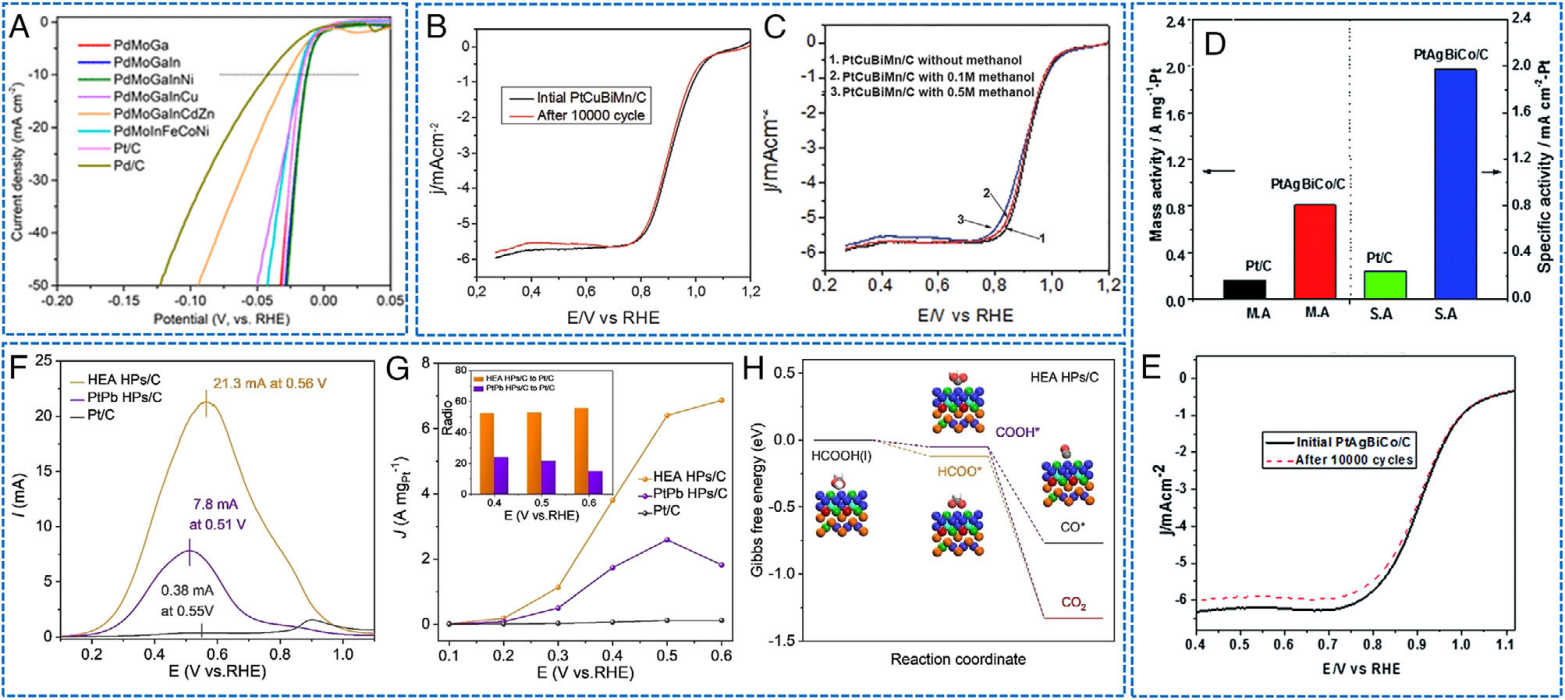
Electrocatalytic reduction and oxidation reaction performances of HEAs nanosheets/nanoplates. (A) HER LSV of PdMoGaInNi. Reproduced with permission.^[^ ^101^ ^]^ Copyright 2022, American Chemical Society. (B) ORR LSV for PtCuBiMn/C before and after 10,000 potential cycles in O_2_‐saturated 0.1 m HClO_4_ solution. (C) ORR polarization curves for PtCuBiMn/C in O_2_‐saturated 0.1 m HClO_4_ that contained 0, 0.1, and 0.5 m methanol. (B, C) Reproduced with permission.^[^ ^104^ ^]^ Copyright 2016, Wiley‐VCH. (D) Comparison of mass activities and specific activities of Pt and PtAgBiCo/C catalysts at 0.9 V. (E) ORR polarization curves for PtAgBiCo/C before and after 10,000 potential cycles in 0.1 m HClO_4_. (D, E) Reproduced with permission.^[^ ^102^ ^]^ Copyright 2017, The Royal Society of Chemistry. (F) CV curves of different catalysts in 0.5 m H_2_SO_4_ + 0.5 m HCOOH. (G) Potential‐dependent current density of different catalysts for FAOR (the inset is the current densities ratio relative to Pt/C). (H) The Gibbs free energy diagram of HEA HPs/C for FAOR. (F–H) Reproduced with permission.^[^ ^103^ ^]^ Copyright 2022, Wiley‐VCH.

In view of the fact that adjusting the hydrogen binding energy (HBE) of a material is one of the effective strategies for optimizing HER electrocatalysts, Fu et al. presented the computer‐facilitated screening of PdMoGaInNi.^[^ ^101^ ^]^ As an exploratory example of 2D HEAs for HER, to achieve a combination of predicted Pt‐free and optimal HBE, PdMoGaInNi nanosheets with the best HBE were synthesized via a solvothermal method. The PdMoGaInNi nanosheets exhibited a superior HER activity with a low overpotential of 13 mV at 10 mA cm^−2^, a higher exchange current density (10^−1.79^ A cm^−2^) in 0.5 m H_2_SO_4_, outperforming commercial Pd/C and Pt/C catalysts (Figure 16A). The PdMoGaInNi nanosheets showed exceptional long‐term durability, lasting at least 200 h at a current density of 100 mA cm^−2^ in a proton exchange membrane water electrolyzer.

#### 5.2.2 ORR

The ORR performance of HEAs nanosheets/nanoplates can be enhanced by introducing transition metals. For example, a recent study by Wang et al. demonstrated the synthesis of tetrametallic alloy nanosheets, exhibiting a uniform thickness ranging from 3 to 4 nm for ORR.^[^ ^104^ ^]^ In order to enhance the catalytic activity of the ternary PtCuBi alloy, the introduction of metal Mn into its porous structure is employed due to its ability to increase band vacancies. Compared with both the ternary PtCuBi alloy and commercial Pt/C catalyst, porous tetrametallic PtCuBiMn nanosheets exhibited excellent catalytic activity (0.69 A ${\mathrm{mg}}_{\mathrm{Pt}}^{-1}$, 0.90 V) and long‐term durability for ORR, with only a marginal 3.8% loss in mass activity after 10,000 cycles at 0.9 V. Additionally, it had a good resistance to methanol crossover effect (Figure 16B,C). Meanwhile, Wang et al. synthesized multi‐metal triangular nanoplates (PtAgBiCo and PtAgBi), which employed the principles of crystal symmetry, oxidation etching, and seed ratio to adjust their activity, stability, methanol tolerance, and the utilization of Pt for ORR in direct methanol fuel cells.^[^ ^102^ ^]^ Electrochemical tests showed that PtAgBiCo nanoplates have excellent electrocatalytic activity and stability for ORR (Figure 16D,E). Its specific activity (1.95 mA cm^−2^) and mass activity (0.81 A ${\mathrm{mg}}_{\mathrm{Pt}}^{-1}$ at 0.90 V) of the PtAgBiCo nanoplates are significantly higher than those of commercial Pt/C catalysts, exhibiting an eightfold increase in specific activity and a fivefold increase in mass activity. Moreover, compared with the commercial Pt/C catalyst, the four‐metal PtAgBiCo nanoplates exhibited a more positive *E*_1/2_ for ORR and an excellent methanol tolerance limit, confirming the feasibility of PtAgBiCo nanoplates as a cathode catalyst for direct methanol fuel cells.

### 5.3 The application of HEAs nanosheets/nanoplates for the electrocatalytic oxidation reaction

HEAs nanosheets/nanoplates have also been verified in the FAOR application. A unique class of PtBiPbNiCo HPs, which has a new nanostructure with an ordered middle entropy core and atomic layered high entropy shell, was synthesized by Zhan et al.^[^ ^103^ ^]^ The characterization outcomes showed that the distinctive HEA HPs consisted of an intermetallic PtBiPb core and a PtBiNiCo shell. Electrochemical tests showed the specific activity and mass activity of HEA HPs/C for FAOR of up to 27.2 mA cm^−2^ and 7.1 A ${\mathrm{mg}}_{\mathrm{Pt}}^{-1}$ (Figure 16F,G). It also had excellent FAOR activity under the operating conditions of a membrane electrode assembly (MEA) in a direct formic acid fuel cell. The MEA power density of HEA HPs/C could achieve 321.2 mW cm^−2^, and the power density attenuation rate reached 55.1%, both of surpassing that of commercial Pd/C. In‐situ characterization and DFT calculations showed that the high tolerance of HEA HPs towards CO* can effectively suppress the dehydration pathway of formic acid molecules to enhance FAOR (Figure 16H). In addition, the HEA HPs also showed commendable activity in other anodic oxidation reactions, such as MOR and EOR.

The exposed active sites, large specific surface area, and the tunable coordinated electronic structure of 2D HEAs nanosheets/nanoplates give rise to significant advantages in the field of electrocatalysis. The composition, structure, synthesis methods, and applications in various electrocatalytic reactions of HEAs nanowires and nanosheets/nanosheets are summarized in Table 2.

**TABLE 2:** The summary of structure, synthetic methods, and electrocatalytic performance of HEAs 1D nanowires and 2D nanosheet/nanoplate.

					Overpotential from CV/LSV curve				
Morphology	Materials	Structure	Synthetic method	Catalytic reaction	Overpotential @ scan rate	Current density	Tafel slope	Electrolytes	Ref.
Nanowires	PtRuRhCoNi	FCC	Solvothermal	HER	−13 mV@5 mV s^−1^	10 mA cm^−2^	23.8 mV dec^−1^	0.5 m H_2_SO_4_	[97]
	AlNiCoRuMo	FCC	Dealloying	HER	−24.5 mV@5 mV s^−1^	10 mA cm^−2^	30.3 mV dec^−1^	1 m KOH	[99]
	AlNiCoRuMo	FCC	Dealloying	OER	≈250 mV @5 mV s^−1^	10 mA cm^−2^	54.5 mV dec^−1^	1 m KOH	[99]

					*E*_1/2_ from CV/LSV curve				
	**Materials**	**Structure**	**Synthetic method**	**Catalytic reaction**	***E*_1/2_ @ scan rate**	**Current density**	**Tafel slope**	**Electrolytes**	**Ref**.
	AlNiCoRuMo	FCC	Dealloying	ORR	0.875 V@5 mV s^−1^	3 mA cm^−2^	‐	0.1 m KOH	[99]

					**Potential from CV curve**				
	Materials	Structure	Synthetic method	Catalytic reaction	Potential @ scan rate	Current density	Selectivity	Electrolytes	Ref.
	PtRuRhCoNi	FCC	Solvothermal	EOR	0.75 V@50 mV s^−1^	≈7.9 mA ${\mathrm{cm}}_{\mathrm{Pt}\mathrm{or}\mathrm{PtRuRh}}^{-2}$	‐	1 m KOH + 1 m C_2_H_5_OH	[97]
	PdPtCuAgAu	FCC	One‐pot solution‐phase	EOR GOR MOR	−0.29 V_SCE_@50 mV s^−1^ −0.08 V_SCE_@50 mV s^−1^ −0.23 V_SCE_@50 mV s^−1^	≈7.5 A ${\mathrm{mg}}_{\mathrm{Pd}+\mathrm{Pt}}^{-1}$ ≈7.3 A ${\mathrm{mg}}_{\mathrm{Pd}+\mathrm{Pt}}^{-1}$ ≈2.1 A ${\mathrm{mg}}_{\mathrm{Pd}+\mathrm{Pt}}^{-1}$	‐	1 m KOH + 1 m C_2_H_5_OH 1 m KOH + 1 m C_2_H_6_O_2_ 1 m KOH + 1 m C_2_H_5_OH	[98]

					***E*_1/2_ from CV/LSV curve**				
Nanosheet/nanoplate	**Materials**	**Structure**	**Synthetic method**	**Catalytic reaction**	***E*_1/2_ @ scan rate**	**Current density**	**Tafel slope**	**Electrolytes**	**Ref**.
	PtAgBiCo	FCC	Solvothermal	ORR	0.9 V_RHE_@10 mV s^−1^	3 mA cm^−2^	‐	0.1 m HClO_4_	[102]
	PtCuBiMn	‐	Solvothermal	ORR	0.9 V_RHE_@10 mV s^−1^	≈3 mA cm^−2^	67 mV dec^−1^	0.1 m HClO_4_	[104]

					**Potential from CV/LSV curve**				
	**Materials**	**Structure**	**Synthetic method**	**Catalytic reaction**	**Potential @ scan rate**	**Current density**	**Selectivity**	**Electrolytes**	**Ref**.
	PtBiPbNiCo	FCC	Wet‐chemical	FAOR MOR EOR	0.56 V@50 mV s^−1^ 0.85 V@50 mV s^−1^ 0.85 V@50 mV s^−1^	27.2 mA cm^−2^ 1.75 A ${\mathrm{mg}}_{\mathrm{Pt}}^{-1}$ 1.4 A ${\mathrm{mg}}_{\mathrm{Pt}}^{-1}$	‐	0.5 m H_2_SO_4_ + 0.5 m HCOOH 0.5 m H_2_SO_4_ + 0.5 m CH_3_OH 0.5 m H_2_SO_4_ + 0.5 m CH_3_CH_2_OH	[103]

“‐” represents not mentioned in the references.

## 6 SYNTHESIS AND APPLICATIONS OF 3D HEAS NANOPOROUS AND OTHER NANOSTRUCTURES FOR ELECTROCATALYSIS

Nanoporous materials have exhibited unparalleled physical and chemical properties as compared with other nanostructures and bulk materials due to their large number of surface metal sites, interconnected porous networks, as well as nano‐sized crystal walls, making them promising candidates for gas absorption, separation and heterogeneous catalysis.^[^ ^105^ ^]^ 3D HEA nanoporous materials therefore have shown huge potential in a wide range of electrocatalytic reactions, including HER, OER, CO_2_RR, and so on.

### 6.1 The synthetic methods of nanoporous HEAs (dealloying method)

**FIGURE 17:**
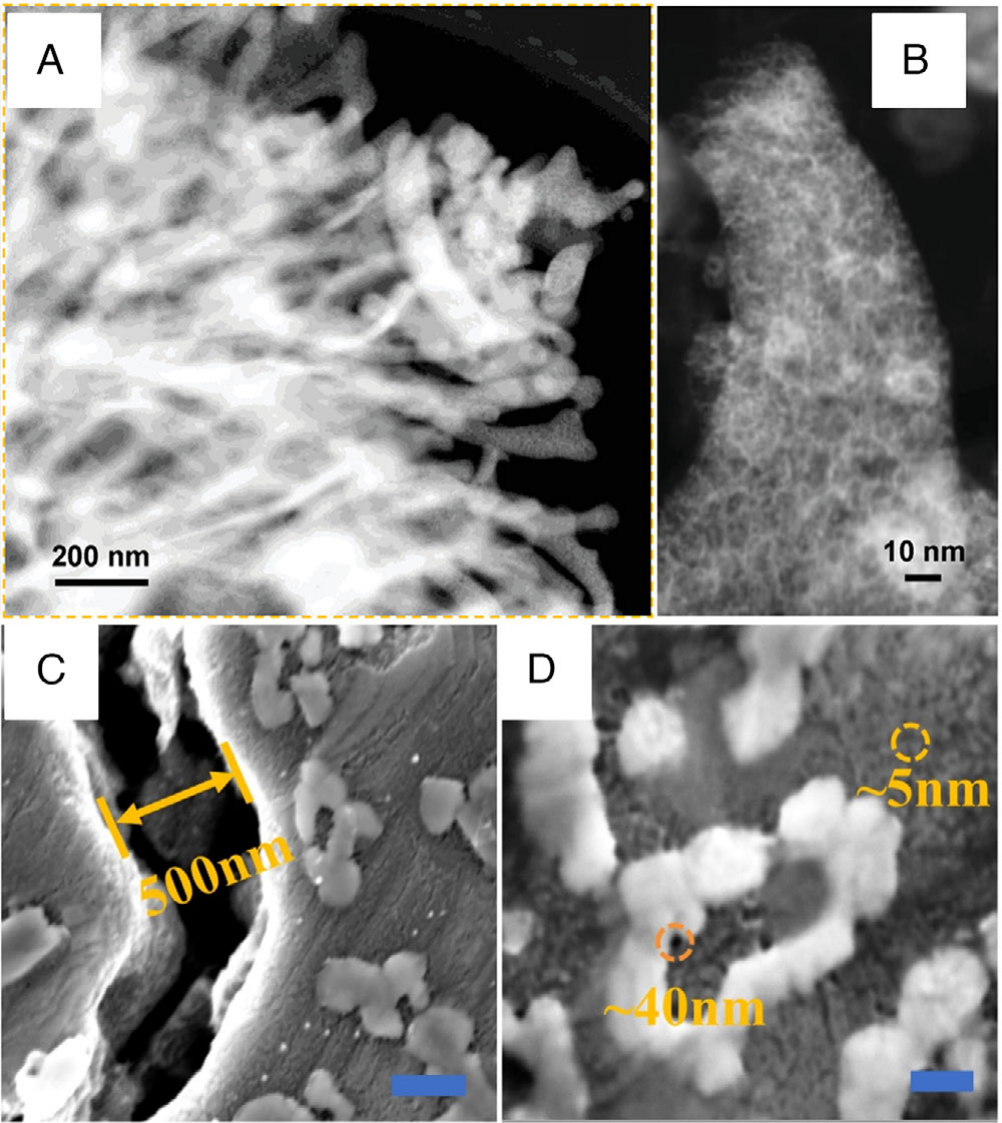
The TEM images of nanoporous HEAs. (A, B) STEM images of the dealloyed AlNiCoIrMo np‐HEA at various magnifications. (A, B) Reproduced with permission.^[^ ^53^ ^]^ Copyright 2019, Wiley‐VCH. (C, D) SEM images of the NiCoFeMoMn np‐HEA. Scale bars: (C) 300 nm, (D) 100 nm. (C, D) Reproduced with permission.^[^ ^107^ ^]^ Copyright 2022, Elsevier.

Nanoporous HEAs (np‐HEAs) were usually obtained by chemically etching or dealloying of the bulk HEAs.^[^ ^106^ ^]^ The key to this dealloying process is to remove part of the original material to generate porosity. For example, HEAs with metal Al were first synthesized, and then Al was etched by acid or alkali to obtain a specific nanoporous structure, such as the nanoporous AlNiCoIrMo HEAs.^[^ ^53^ ^]^ The AlNiCoIrMo np‐HEA has an ultra‐thin nano ligament size of ≈2 nm (Figure 17A,B). Furthermore, Liu et al. reported an electrochemical dealloying technique for synthesizing nanoporous HEAs.^[^ ^107^ ^]^ To be specific, NiCoFeMoMn HEA strip with a thickness of 25 µm and width of approximately 2 mm was first synthesized through arc melting and single‐roll melt spinning. The porous structure of NiCoFeMoMn HEA was created by electrochemical dealloying in a 1 m (NH_4_)_2_SO_4_ solution to remove Mn. After 6 h of dealloying treatment, surface cracks with widths ranging from 300 to 500 nm were observed on the NiCoFeMoMn np‐HEA. The NiCoFeMoMn np‐HEA showed a layered nanopore framework, featuring small surface nanopores of about 5 nm and large nanopores of about 40 nm (Figure 17C,D).

### 6.2 The application of nanoporous HEAs for electrocatalytic reduction reactions

#### 6.2.1 HER

**FIGURE 18:**
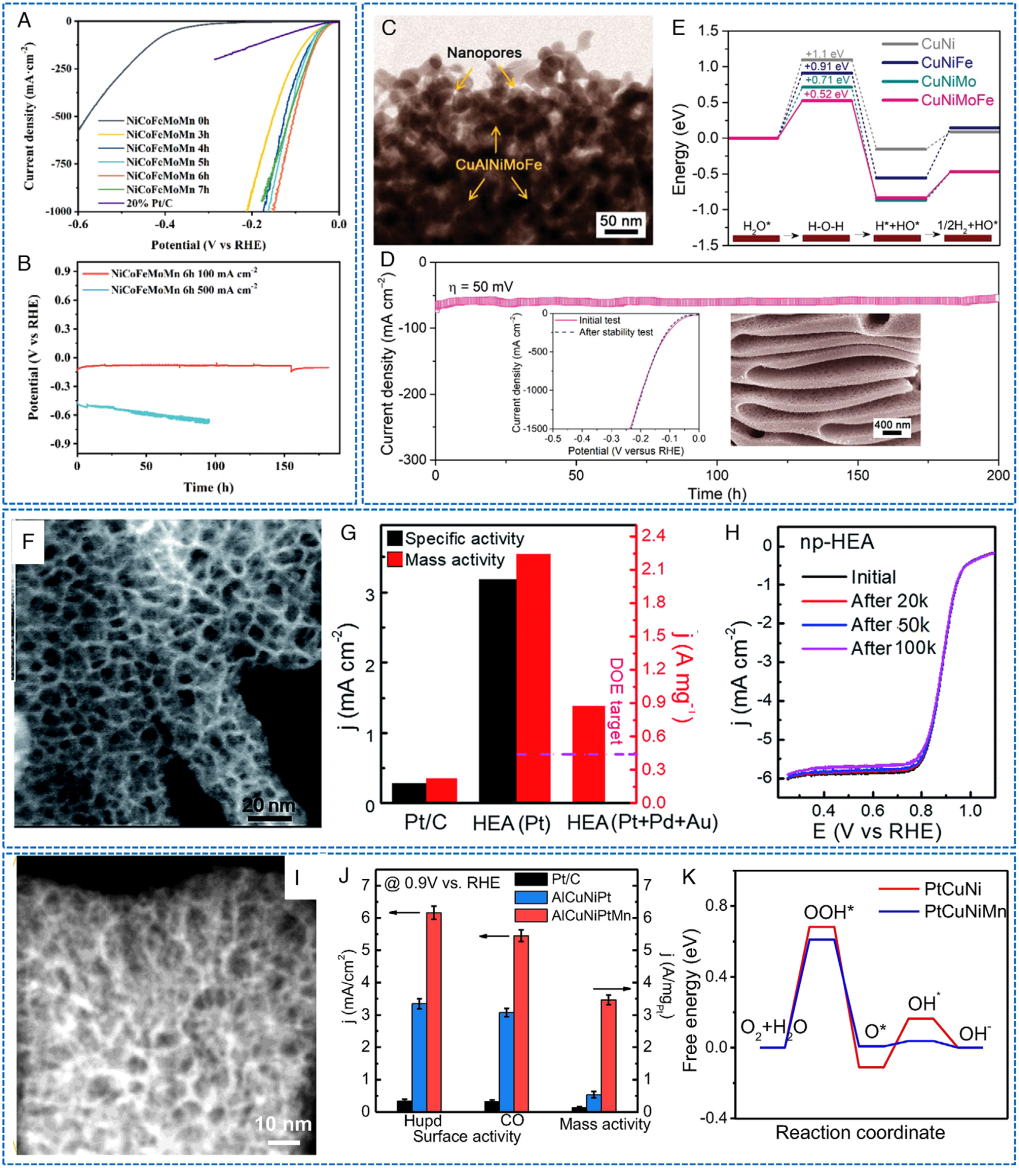
TEM images and electrocatalytic reduction reaction performances of nanoporous HEAs. (A) HER polarization curves of various catalysts in 1 m KOH. (B) The stability curves of the nanoporous NiCoFeMoMn at 100 and 500 mA cm^−2^. (A, B) Reproduced with permission.^[^ ^107^ ^]^ Copyright 2022, Elsevier. (C) STEM image of nanoporous CuAlNiMoFe with uniform small nanopores. (D) Long‐term stability test of nanoporous CuAlNiMoFe. (E) Theoretical calculation of HER on surface high‐entropy CuNiMoFe alloy. (C–E) Reproduced with permission.^[^ ^108^ ^]^ Copyright 2020, Wiley‐VCH. (F) Dark‐field STEM image of senary AlNiCuPtPdAu np‐HEA. (G) Electrochemical active surface area and mass activities at 0.9 V and (H) durability test in O_2_‐saturated 0.1 m HClO_4_. (F–H) Reproduced with permission.^[^ ^109^ ^]^ Copyright 2019, The Royal Society of Chemistry. (I) STEM images of the AlCuNiPtMn np‐HEA. (J) Pt mass and surface specific activities at 0.9 V. (K) Free energy profiles of the ORR steps. (I–K) Reproduced with permission.^[^ ^110^ ^]^ Copyright 2020, Elsevier.

Due to the high specific surface area of their porous structure, which maximizes the exposure of the active sites, nanoporous HEAs composed of non‐precious metals are widely used for HER. For example, Liu et al. synthesized a nanoporous NiCoFeMoMn material serving as an electrocatalyst.^[^ ^107^ ^]^ The porous structure of NiCoFeMoMn can expand the electrochemically active region and expose a greater number of electrochemically active sites for electrocatalytic HER in alkaline electrolytes. The HER of NiCoFeMoMn np‐HEA has shown a very low overpotential of 150 mV at 1000 mA cm^−2^, a Tafel slope of 29 mV dec^−1^, and good stability, while saving material costs relative to Pt (Figure 18A,B). DFT calculations revealed that the ultra‐high HER activity of the catalyst stems from the synergistic interplay between the segregation region generated by the spinodal decomposition that enhances hydrogen adsorption and the non‐segregation region that enhances H_2_O adsorption. Electrocatalytic HER provides the potential for using Pt‐free electrocatalysts in large‐scale electrochemical production of pure H_2_ fuels in alkaline and neutral solutions. However, most advanced non‐precious transition metal‐based electrocatalytic materials work at a high overpotential. Yao et al. reported on the development of a monolithic nanoporous high‐entropy multicomponent CuAlNiMoFe electrode, which demonstrates remarkable efficiency as an electrocatalyst for HER in both alkaline and neutral electrolytes.^[^ ^108^ ^]^ The hierarchical nanoporous CuAlNiMoFe were composed of uniformly interpenetrating nanopores and interconnected metal ligaments, with a characteristic length as short as about 10 nm (Figure 18C). This distinctive bimodal porous structure promoted the transfer of electrons along the interconnected Cu ligament and provided accessibility to the electroactive sites on the surface of high‐entropy CuNiMoFe due to large lamellar channels and small nanopores. The monolithic nanoporous CuAlNiMoFe electrode has shown a low Tafel slope, a high electrocatalytic activity, and excellent durability in both alkaline and neutral electrolytes (Figure 18D). In different electrolytes containing 1 m KOH and 1 m phosphate buffer solution (PBS) with a value of 7, high current densities of approximately1840 and 100 mA cm^−2^ could be achieved at overpotentials of around 240 and 183 mV, respectively. It was found that the HEA surface composed of Cu, Ni, Mo, and Fe atoms acts as a bifunctional site for promoting the dissociation of water and facilitating the adsorption/desorption of H* (Figure 18E).

#### 6.2.2 ORR

One way to adjust the activity of Pt for ORR is to alloy Pt with other suitable metal elements. The recent focus of research has been on reducing the amount of Pt while maintaining the ORR activity. Qiu et al. developed a versatile and scalable strategy for preparing ultra‐thin np‐HEAs with precisely controlled composition via integrating scalable alloy melting, rapid cooling, and dealloying.^[^ ^109^ ^]^ In specific, senary AlNiCuPtPdAu and senary all‐non‐precious metal AlNiCuMoCoFe np‐HEA with ligament sizes ranging from 2 to 3 nm were synthesized, and the composition was accurately controlled through dealloying the designed precursor alloy (Figure 18F). The AlNiCuPtPdAu np‐HEA with a small amount of Pt loading exhibited a tenfold increase in mass activity (2.24 A mg^−1^) than that of Pt/C for ORR in electrocatalysis, while maintaining 92.5% of its original activity after 100,000 electrochemical cycles (Figure 18G,H). For nanoscale HEAs, the ensemble effect, ligand effect and surface strain can be widely adjusted via manipulating the type and composition of elements. For instance, Li et al. found an AlCuNiPtMn np‐HEA catalyst with optimized surface strain and ORR electronic properties by studying the composition effect.^[^ ^110^ ^]^ Specifically, this work demonstrated a top‐down dealloying synthesis technique, in which five incompatible metals were incorporated into a nanoscale solid phase (Figure 18I). By pre‐determining Al, Cu, Ni, Pt, and alternately selecting the fifth metal (Pd, V, Co, or Mn, etc.), a class of np‐HEAs with Pt content of about 20–30 at% was obtained. In particular, AlCuNiPtMn np‐HEA exhibited an ultra‐high ORR activity and a record‐low Pt content. Electrochemical tests showed that the AlCuNiPtMn np‐HEA catalyst exhibits a maximum *E*_1/2_ of about 0.945 V in an acidic medium, and has excellent electrochemical stability (Figure 18J,K).

### 6.3 The application of nanoporous HEAs for electrocatalytic oxidation reaction

**FIGURE 19:**
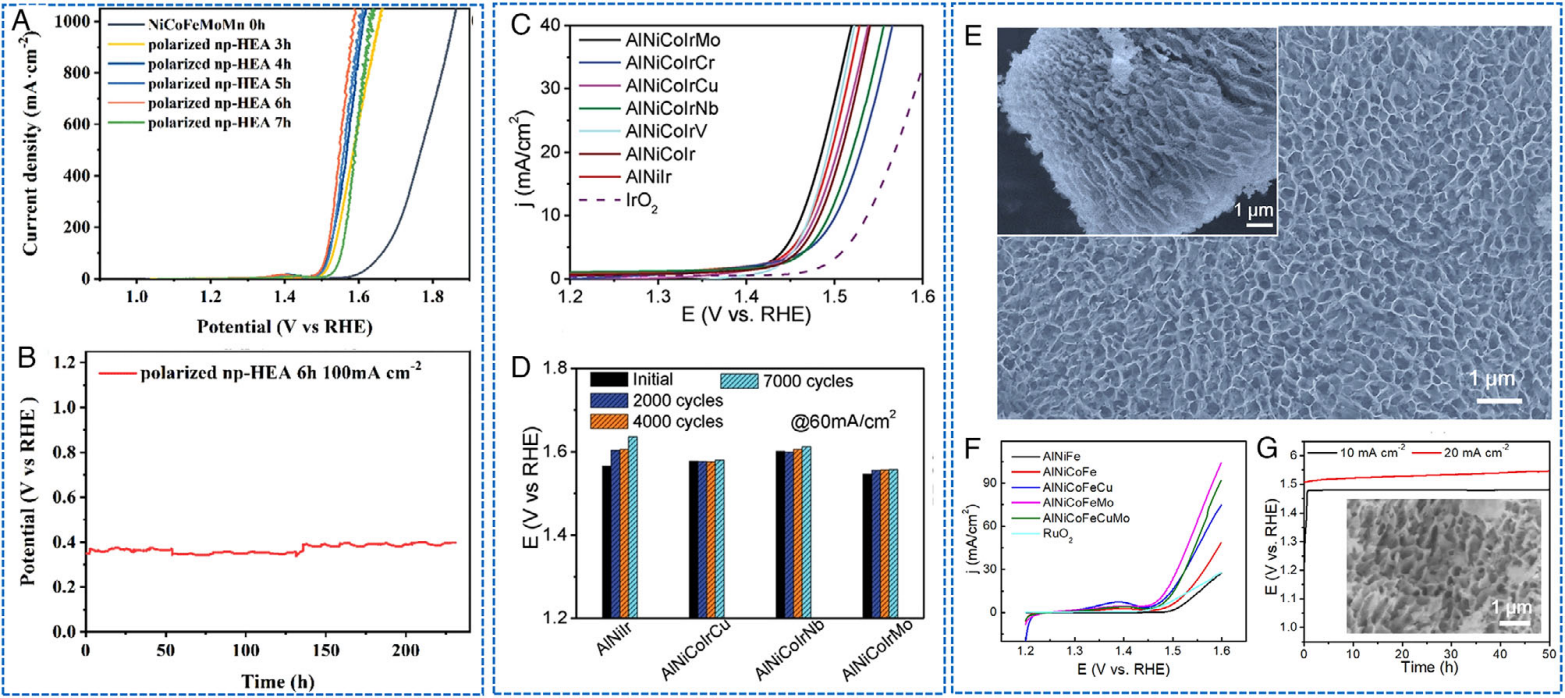
TEM images and electrochemical OER performances of nanoporous HEAs. (A) OER polarization curves for polarized np‐HEA in 1 m KOH solution. (B) The time‐current curves of the polarized np‐HEA 6 h at a current density of 100 mA cm^−2^ without iR. (A, B) Reproduced with permission.^[^ ^107^ ^]^ Copyright 2022, Elsevier. (C) OER LSV of AlNiIr, AlNiCoIr, AlNiCoIrX (X: Mo, Cr, Cu, Nb, V), and IrO_2_. (D) Potential changes of these samples to reach 60 mA cm^−2^ in 0.5 m H_2_SO_4_. (C, D) Reproduced with permission.^[^ ^53^ ^]^ Copyright 2019, Wiley‐VCH. (E) Plane‐view and section‐view SEM (inset in (E)) of np‐AlNiCoFeMo. (F) OER LSV curves of the np‐HEAs, np‐AlNiCoFe, np‐AlNiFe, and RuO_2_ in 1.0 m KOH. (G) The potential change with time, and the inset is the SEM image after 50 h test of np‐AlNiCoFeMo. (E–G) Reproduced with permission.^[^ ^111^ ^]^ Copyright 2019, American Chemical Society.

Nanoporous HEAs have also been applied in electrocatalytic OER. The nanoporous NiCoFeMoMn reported by Liu et al. has shown an exceptional OER activity with only 350 mV overpotential at 1000 mA cm^−2^ (Figure 19A,B).^[^ ^107^ ^]^ The alkaline electrolyzer, utilizing this electrocatalyst as both the anode and cathode, demonstrated remarkable stability with a cell voltage of 1.47 V to achieve a stable current density of 10 mA cm^−2^, and ran continuously for over 375 h. In addition, Qiu et al. developed a nanoporous AlNiCoIrMo HEA for acidic OER (Figure 19C,D).^[^ ^53^ ^]^ The AlNiCoIrMo np‐HEA catalysts had the advantages of low Ir loading (≈20 at%) and high activity (the overpotentials were 233 and 255 mV at 10 or 20 mA cm^−2^ in acidic media), and were very promising for activity customization/maximization. Qiu et al. designed a multi‐component Al‐based precursor alloy and dealloyed in an alkaline solution, a multi‐component np‐AlNiCoFeMo was obtained (Figure 19E).^[^ ^111^ ^]^ In this work, different np‐HEAs were synthesized by well‐mixed non‐precious metals, and it was observed that the composition of the alloy significantly influences the enhancement of OER activity. The np‐AlNiCoFeMo showed the highest OER activity in alkaline electrolytes (240 mV at 10 mA cm^−2^) (Figure 19F,G).

### 6.4 Synthesis and applications of other 3D morphologies of HEAs for electrocatalysis

In addition to the well‐defined HEAs nanoporous structures discussed above, other 3D structures of HEAs have also been synthesized for electrocatalysis. Herein, we discuss the synthetic methods and electrocatalytic applications of those 3D structures with some typical examples in the following sections.

#### 6.4.1 The synthesis and application of convex cube‐shaped HEAs for HER, OER, and ORR

**FIGURE 20:**
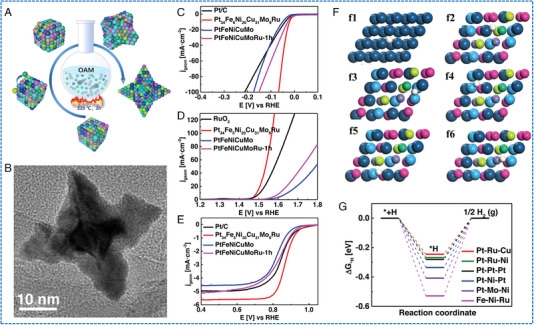
Synthesis and electrochemical reaction performances of convex cube‐shaped HEAs. (A) Schematic of synthesis CNT‐supported convex cube‐shaped Pt_34_Fe_5_Ni_20_Cu_31_Mo_9_Ru HEA. (B) TEM image of Pt_34_Fe_5_Ni_20_Cu_31_Mo_9_Ru. (C) HER LSV. (D) OER LSV. (E) ORR polarization curves of Pt_34_Fe_5_Ni_20_Cu_31_Mo_9_Ru, PtFeNiCuMo, and PtFeNiCuMoRu‐1 h. (F) DFT atomic configurations at Pt‐(111) and Pt_34_Fe_5_Ni_20_Cu_31_Mo_9_Ru HEA surface with adsorbed *H at different hollow sites. Pt‐(111): (f1) Pt‐Pt‐Pt. HEA: (f2) Pt‐Ru‐Cu, (f3) Pt‐Ru‐Ni, (f4) Pt‐Ni‐Pt, (f5) Pt‐Mo‐Ni, (f6) Fe‐Ni‐Ru (Navy: Pt, blue: Ni, yellow: Mo, green: Ru, pink: Cu, purple: Fe, white: hydrogen). (G) ΔG_*H_ profiles on different catalytic sites at Pt_34_Fe_5_Ni_20_Cu_31_Mo_9_Ru HEA surface in comparison with Pt‐(111). Reproduced with permission.^[^ ^112^ ^]^ Copyright 2022, Wiley‐VCH.

The synthesis of shape‐controlled HEA catalysts, particularly those with high refractive index surfaces, is challenging. Chen et al. reported a unique convex cubic Pt_34_Fe_5_Ni_20_Cu_31_Mo_9_Ru catalyst by a solvothermal method (Figure 20A).^[^ ^112^ ^]^ Acetylacetone metal salts were used as metal precursors, Mo(CO)_6_ was used as a reducing agent, carbon nanotubes were as a carrier, CTAC was used as a structure‐directing agent, and oleylamine was used as a solvent. The key point is the addition of the Ru element, which is crucial in determining the formation of a convex cubic morphology. The cube‐shaped nanocrystals would be obtained when Ru was not added. After adding Ru, the pyramid was selectively grown on each facet of the cube‐shaped nanocrystals, resulting in the formation of a convex cube morphology (Figure 20B).

The catalytic activity of nano‐metal catalysts is significantly affected by specific surface facets, strains, and the coordination environment of surface atoms.^[^ ^113^ ^]^ The catalytic activity of high‐index facets is typically higher than that of low‐index facets due to their lower coordination surface atoms, making them more active in breaking and forming chemical bonds. The current state of affairs indicates that the majority of reported HEA typically consists of spherical nanoparticles wrapped by low‐index facets.^[^ ^44^ ^]^ The synthesis of nanocrystals featuring high‐index crystal planes and the investigation of the correlation between catalytic performance and the morphology and/or structure of nano‐HEAs need to be explored. Chen et al. reported a unique convex cubic Pt_34_Fe_5_Ni_20_Cu_31_Mo_9_Ru catalyst by a solvothermal method.^[^ ^112^ ^]^ The Pt_34_Fe_5_Ni_20_Cu_31_Mo_9_Ru catalyst exhibited outstanding electrocatalytic performance for HER, OER, and ORR. For HER, in 1 m KOH, the overpotential was only 20 mV at 10 mA cm^−2^, and the Tafel slope was 27 mV dec^−1^, the Pt_34_Fe_5_Ni_20_Cu_31_Mo_9_Ru showed excellent electrochemical stability and mass activity up to 11.4 A ${\mathrm{mg}}_{\mathrm{Pt}}^{-1}$ (Figure 20C). For OER, in 1 m KOH, the overpotential was 259 mV at 10 mA cm^−2^ and the Tafel slope was 39 mV dec^−1^, both of which were better than RuO_2_. Pt_34_Fe_5_Ni_20_Cu_31_Mo_9_Ru had a high OER FE of 95% and excellent stability (Figure 20D). For ORR, in 0.1 m HClO_4_, the *E*_1/2_ was 0.87 V, and the Tafel slope was 69 mV dec^−1^ (Figure 20E). The excellent ORR performance of Pt_34_Fe_5_Ni_20_Cu_31_Mo_9_Ru catalyst may be attributed to the synergistic and strain effect between Pt and other metals, thereby optimizing the interaction with the adsorption intermediate (O*/OOH*). DFT calculations demonstrated that Ru doping, convex cube shape, and multi‐metal components play essential roles in achieving high catalytic activity for Pt_34_Fe_5_Ni_20_Cu_31_Mo_9_Ru (Figure 20F,G). This work revealed the growth kinetics of Pt_34_Fe_5_Ni_20_Cu_31_Mo_9_Ru nanocrystals and emphasized the significance of shape‐controlled synthesis in achieving multifunctional catalysis for HEAs.

#### 6.4.2 The synthesis and application of nano‐hollow spherical HEAs for ORR and FAOR

**FIGURE 21:**
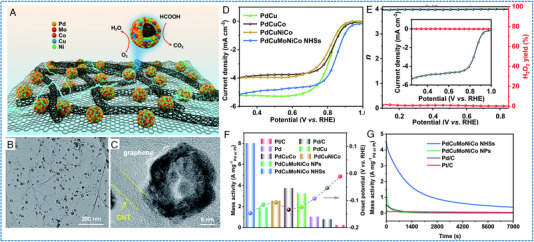
Synthesis and electrochemical ORR and FAOR performances of nano‐hollow spherical HEAs. (A) Schematic illustration of PdCuMoNiCo NHSs/RGO_3_‐CNT for ORR and FAOR. (B) TEM image and (C) HR‐TEM image of PdCuMoNiCo NHSs/RGO_3_‐CNT. (D) ORR LSV curves in O_2_‐saturated 0.1 m HClO_4_. (E) “n” and H_2_O_2_ yields of PdCuMoNiCo NHSs/RGO_3_‐CNT (inset: corresponding disk and ring current of PdCuMoNiCo NHSs/RGO_3_‐CNT, and the Pt ring electrode was maintained at 1.4 V vs RHE). (F) Comparison of the *E_onset_* and mass activities in FAOR. (G) Current–time curves recorded at 0.5 V versus RHE in FAOR. Reproduced with permission.^[^ ^115^ ^]^ Copyright 2022, The Royal Society of Chemistry.

The hollow structure is beneficial to the catalytic reaction owing to its various unique properties, such as shortened reactant path length, improved utilization of metal atoms, and completely exposed active sites.^[^ ^114^ ^]^ However, due to the harsh manufacturing conditions and intricate composition of HEAs, the synthesis of hollow HEAs is still a significant challenge. Zuo et al. expanded upon their previously reported synthesis of PdCu nanoboxes by introducing other metals (Ni, Co, and Mo) with distinct electronic structures and electronegativity to form a stable HEAs system.^[^ ^115^ ^]^ Specifically, with the help of a removable ligand (glutamic acid salt), PdCuMoNiCo hollow spheres were synthesized by solvothermal method on the carbon hybrid of reduced graphene oxide and carbon nanotubes (PdCuMoNiCo NHSs/RGO_3_‐CNT) (Figure 21A–C). The experimental results revealed that the as‐synthesized PdCuMoNiCo NHSs/RGO_3_‐CNT is a preeminent electrocatalytic performance, characterized by high activity and stability towards acidic ORR and FAOR. For ORR, it exhibited an *E*_1/2_ of 0.86 V, a mass activity reached 0.882 A ${\mathrm{mg}}_{\mathrm{Pd}}^{-1}$, which was 1.40 times higher compared to commercial Pt/C (0.630 A ${\mathrm{mg}}_{\mathrm{Pt}}^{-1}$). This electrocatalyst also showed good durability in 0.1 m HClO_4_ (Figure 21D,E). The experimental results revealed that in the absence of Pd, the ORR activity of CuMoNiCo on RGO_3_‐CNT was negligible, indicating that Pd serves as the primary active center for acid ORR, while other elements help to regulate the adsorption energy of intermediates and facilitate their conversion. It was found that the introduction of heteroatoms necessarily causes changes in the intrinsic strain of Pd, and excessive strain changes will adversely affect the catalytic performance. Consequently, the hollow structure enhanced the electrochemical‐specific surface area, and reduced the influence of strain changes caused by heteroatoms, thereby regulating the performance of the catalyst. As a result, PdCuMoNiCo NHSs/RGO_3_‐CNT reached a peak current density of 70.96 mA cm^−2^ and showed a more negative *E_onset_* (−0.147 V), indicating that PdCuMoNiCo NHSs/RGO_3_‐CNT had a faster kinetics of FAOR. PdCuMoNiCo‐NHS/RGO_3_‐CNT also had a high mass activity value of 8.030 A ${\mathrm{mg}}_{\mathrm{Pd}}^{-1}$ (Figure 21F,G).

#### 6.4.3 The synthesis and application of HEAs aerogels for CO_2_RR

**FIGURE 22:**
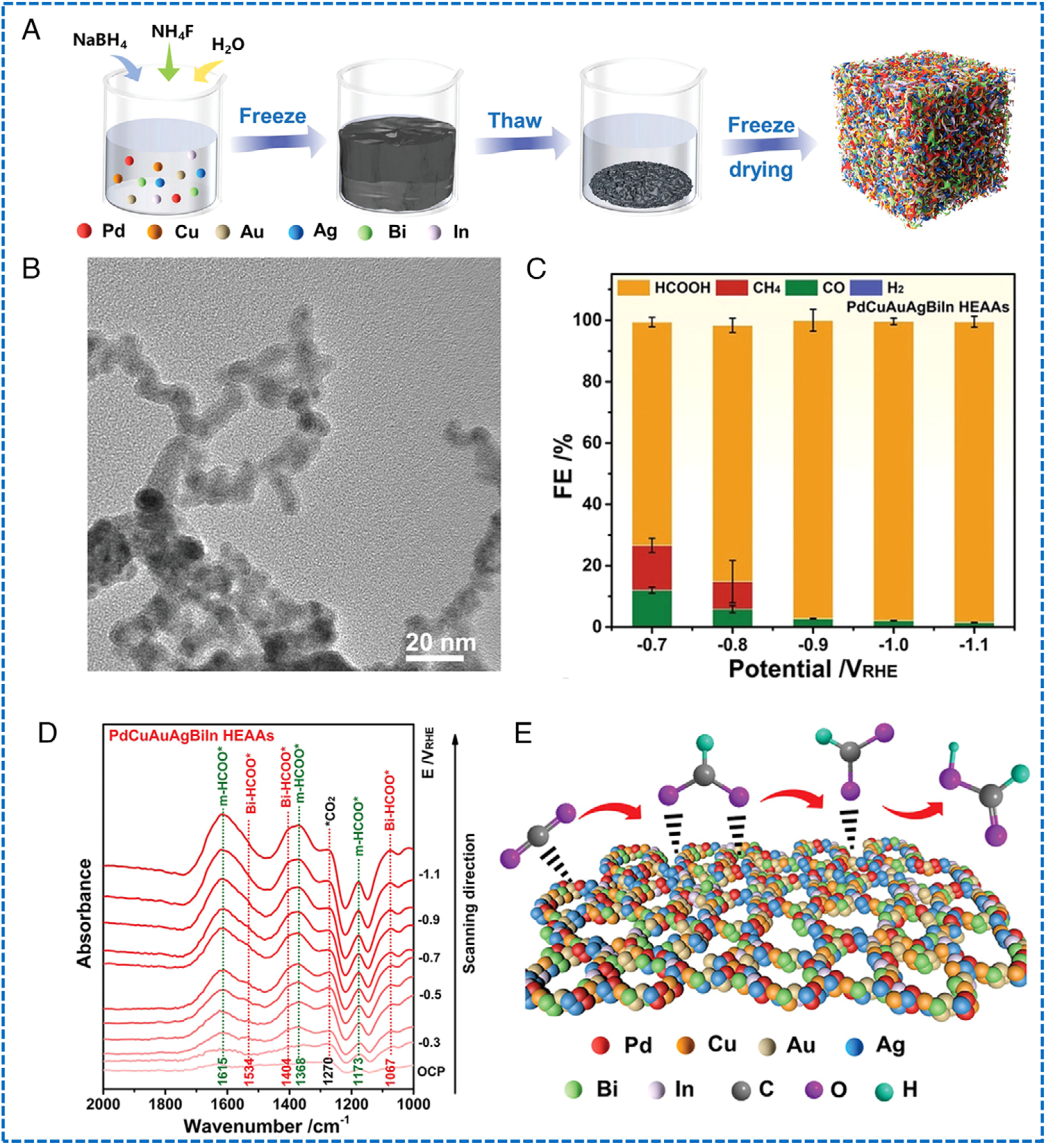
Synthesis and electrochemical CO_2_RR performances of HEAs aerogels. (A) Schematic illustration of the preparation of PdCuAuAgBiIn HEAAs. (B) TEM image of PdCuAuAgBiIn HEAAs. (C) Reduction potential dependent FEs measured of PdCuAuAgBiIn HEAAs. (D) In‐situ ATR‐IRAS obtained during chronopotentiometry in a potential window −0.3 V_RHE_ to −1.2 V_RHE_ under CO_2_RR. (E) Schematic for boosted HCOOH generation. Reproduced with permission.^[^ ^118^ ^]^ Copyright 2022, Wiley‐VCH (FE: Faradaic efficiency).

A unique 3D architecture is advantageous in terms of enhancing the exposure of additional active sites. Metal aerogel is a 3D self‐supported solid network.^[^ ^116^ ^]^ The high porosity and large specific surface area can facilitate the provision of sufficient catalytic active sites and mass transfer channels, thereby enhancing both mass transfer and charge transfer in the electrocatalytic process.^[^ ^117^ ^]^ The combination of HEAs and aerogels in the form of high‐entropy alloy aerogels (HEAAs) presents a promising platform for catalytic reactions, capitalizing on the unique advantages offered by both materials. Li et al. reported a series of HEAAs synthesized by a freeze‐thaw method (using co‐reduction of metals) as highly active and durable electrocatalysts for CO_2_RR (Figure 22A,B).^[^ ^118^ ^]^ In particular, PdCuAuAgBiIn HEAAs achieved almost 100% C_1_ product with FE in the range of −0.7 to −1.1 V versus RHE. The maximum FE of formic acid (FE_HCOOH_) was 98.1% at −1.1 V versus RHE, which was higher than that of PdCuAuAgBiIn HEA particles and Pd metal aerogels (Figure 22C). Especially, the current density and FE_HCOOH_ reached respectively 200 mA cm^−2^ and 87% in the flow cell, respectively. This work has shown that the robust interaction between various metals and surface unsaturated sites in PdCuAuAgBiIn HEAAs effectively regulates the electronic structure of different metals, optimizes the adsorption and desorption strength of HCOO* intermediates on the catalyst surface, and hence increases the production of HCOOH (Figure 22D,E).

#### 6.4.4 The synthesis and application of mesoporous HEAs nanospheres for HER

**FIGURE 23:**
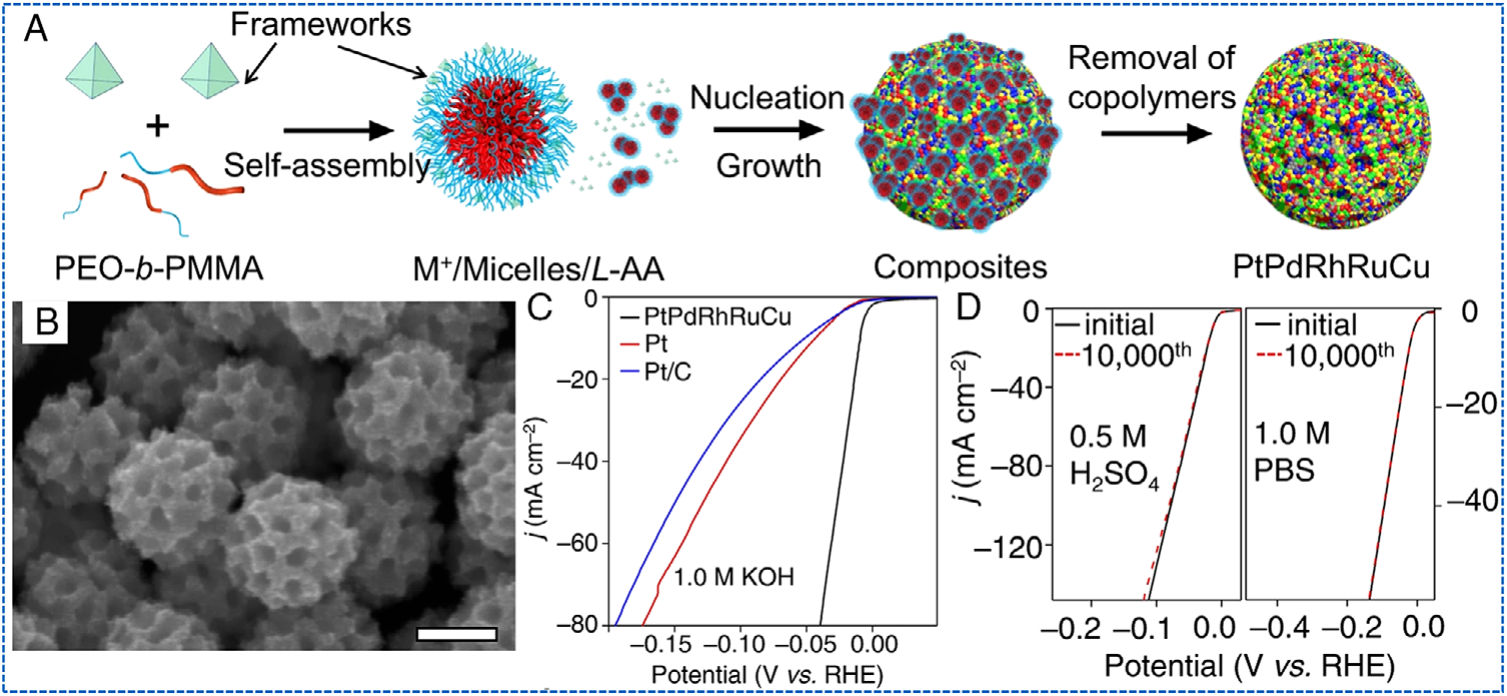
Synthesis and electrochemical HER performances of mesoporous HEAs nanospheres. (A) Schematic illustration of PtPdRhRuCu MMNs. (B) SEM (scale bar: 100 nm) of PtPdRhRuCu MMNs. (C) HER LSV curves in 1.0 m KOH after manual iR correction. (D) CV stability tests of PtPdRhRuCu MMNs in 0.5 m H_2_SO_4_ and 1.0 m PBS solutions. Reproduced with permission.^[^ ^119^ ^]^ Copyright 2023, Nature Publishing Group.

Mesoporous materials with regular and ordered pore structures have received much attention in electrocatalysis, especially the mesoporous structures of HEAs. Recently, Kang et al. synthesized core–shell HEAs mesoporous nanospheres (PtPdRhRuCu MMNs) with a tunable composition and exposed porous structure through a one‐pot wet‐chemical reduction method (Figure 23A,B).^[^ ^119^ ^]^ The diblock copolymer was utilized as the soft template in the synthesis process, and the formation process of PtPdRhRuCu MMNs mesoporous structure was revealed. PtPdRhRuCu MMNs demonstrated exceptional HER activity (overpotential was 10/13/28 mV at 10 mA cm^−2^) under alkaline, acidic, and neutral conditions (Figure 23C,D). The accelerated kinetics of HER were derived from the synergistic interaction among various components and the mesoporous structure with excellent mass/electron transport properties.

The reason for the excellent electrocatalytic activity of HEAs porous structure is that the presence of porosity increases the reaction area and the pore structure optimizes the electron/proton transport path. The 3D morphology will lead to the maximization of active area of the reaction and the stable existence of the active site, thus to enhance the activity and stability. Based on the above two points, the fine structure construction and electronic environment regulation of the catalysts are the keys for achieving excellent electrocatalytic performance.

The material composition, crystal structure, synthesis method, and performance in various electrocatalytic reactions of nanoporous and 3D morphologies of HEAs are summarized in Table 3.

**TABLE 3:** The summary of structure, synthetic methods, and electrocatalytic performance of HEAs with nanoporous and other 3D morphologies.

					Overpotential from CV/LSV curve				
Morphology	Materials	Structure	Synthetic method	Catalytic reaction	Overpotential @ scan rate	Current density	Tafel slope	Electrolytes	Ref.
Nanoporous	AlNiCoIrMo	‐	Dealloying	HER	−18.5 mV@5 mV s^−1^	10 mA cm^−2^	33 mV dec^−1^	0.5 m H_2_SO_4_	[53]
	NiCoFeMoMn	FCC	One‐step dealloying	HER	150 mV@1 mV s^−1^	1000 mA cm^−2^	29 mV dec^−1^	1.0 m KOH	[107]
	PtPdRhIrNi	FCC	Eutectic‐directed self‐templating strategy	HER	−22 mV@5 mV s^−1^ −55 mV@5 mV s^−1^	10 mA cm^−2^	21.6 mV dec^−1^ 44.8 mV dec^−1^	0.5 m H_2_SO_4_ 1.0 m KOH	[120]
	AlAgAuCoCuFeIrMoNiPdPtRhRuTi	FCC	Arc melting	HER	32 mV @ 5 mV s^−1^	10 mA cm^−2^	30.1 mV dec^−1^	0.5 m H_2_SO_4_	[121]
	AlNiCoIrMo	‐	Dealloying	OER	233 mV @ 5 mV s^−1^	10 mA cm^−2^	55.2 mV dec^−1^	0.5 m H_2_SO_4_	[53]
	NiCoFeMoMn	FCC	One‐step dealloying	OER	350 mV@1 mV s^−1^	1000 mA cm^−2^	37 mV dec^−1^	1.0 m KOH	[107]
	AlNiCoFeMo	FCC	Dealloying	OER	240 mV@5 mV s^−1^	10 mA cm^−2^	46 mV dec^−1^	1.0 m KOH	[111]
	AlAgAuCoCuFeIrMoNiPdPtRhRuTi	FCC	Arc melting	OER	258 mV @ 5 mV s^−1^	10 mA cm^−2^	84.2 mV dec^−1^	0.5 m H_2_SO_4_	[121]
	CoCrFeNiMo	FCC	Microwave sintering	OER	220 mV @ 2 mV s^−1^	10 mA cm^−2^	59.0 mV dec^−1^	1.0 m KOH	[122]
	AlCrCuFeNi	FCC	Vacuum induction melting + gas atomization + acidic etching	OER	270 mV@5 mV s^−1^	10 mA cm^−2^	77.5 mV dec^−1^	1.0 m KOH	[123]

					***E*_1/2_ from CV/LSV curve**				
	**Materials**	**Structure**	**Synthetic method**	**Catalytic reaction**	***E*_1/2_ @ scan rate**	**Current density**	**Tafel slope**	**Electrolytes**	**Ref**.
	AlNiCuMoCoFe	FCC	Melting‐fast cooling‐dealloying	ORR	0.9 V versus RHE @5 mV s^−1^	3.2 mA cm^−2^	‐	0.1 m HClO_4_	[109]
	AlCuNiPtMn	FCC	Dealloying	ORR	0.945 V versus RHE @5 mVs^−1^	≈4 mA cm^−2^	‐	0.1 m HClO_4_	[110]
	PtRuCuOsIr	FCC	Dealloying	ORR	0.9 V versus RHE @10 mV s^−1^	3 mA cm^−2^	‐	0.1 m HClO_4_	[124]

					**Potential from CV curve**				
	**Materials**	**Structure**	**Synthetic method**	**Catalytic reaction**	**Potential @ scan rate**	**Current density**	**Selectivity**	**Electrolytes**	**Ref**.
	PtRuCuOsIr	FCC	Dealloying	MOR	0.61 V versus SCE@50 mV s^−1^	3 mA cm^−2^	‐	0.5 m H_2_SO_4_ + 0.5 m CH_3_OH	[124]

					**Potential from CV/LSV curve**				
	**Materials**	**Structure**	**Synthetic method**	**Catalytic reaction**	**Overpotential @ scan rate**	**Current density**	**Tafel slope**	**Electrolytes**	**Ref**.
Convex cube‐shaped	PtFeNiCuMoRu	FCC	Solvothermal	HER OER ORR	20 mV@5 mV s^−1^ 259 mV@5 mV s^−1^ 0.87 V versus RHE@10 mV s^−1^ (*E*_1/2_)	10 mA cm^−2^ 10 mA cm^−2^ ≈2.9 mA cm^−2^	27 mV dec^−1^ 39 mV dec^−1^ 69 mV dec^−1^	1 m KOH 1 m KOH 0.1 m HClO_4_	[112]

					**Potential from CV curve**				
	**Materials**	**Structure**	**Synthetic method**	**Catalytic reaction**	**Potential @ scan rate**	**Current density**	**Tafel slope /Selectivity**	**Electrolytes**	**Ref**.
Nano‐hollow spherical	PdCuMoNiCo	FCC	Solvothermal	ORR FAOR	0.86 V_RHE_@5 mV s^−1^ (E_1/2_) 0.58 V @50 mV s^−1^	≈3 mA cm^−2^ 70.96 mA cm^−2^	‐	0.1 m HClO_4_ 0.1 m HClO_4_ + 0.5 m HCOOH	[115]

					**Potential from chronoamperometric response**				
	**Materials**	**Structure**	**Synthetic method**	**Catalytic reaction**	**Potential @ scan rate**	**Current density**	**Selectivity**	**Electrolytes**	**Ref**.
HEAs aerogels	PdCuAuAgBiIn	FCC	freeze‐thaw	CO_2_ RR	−0.8 V_RHE_	−150 mA cm^−2^	91.3%	0.1 m KHCO_3_	[118]

					**Overpotential from CV/LSV curve**				
	**Materials**	**Structure**	**Synthetic method**	**Catalytic reaction**	**Overpotential @ scan rate**	**Current density**	**Tafel slope**	**Electrolytes**	**Ref**.
Mesoporousnanospheres	PtPdRhRuCu	FCC	wet‐chemical	HER	10 mV 13 mV 28 mV	10 mA cm^−2^	87 mV dec^−1^ ‐ ‐	1 m KOH 0.5 m H_2_SO_4_ 1 m PBS	[119]

“‐” represents not mentioned in the references.

## 7 SUMMARY AND OUTLOOK

HEAs, by virtue of their abundant active sites, robust synergistic effect of polymetallic components, and high entropy stabilization effect, have shown exceptional electrochemical properties as electrocatalysts. The advancement of synthetic methods is of practical significance in the exploration of advanced HEAs and their electrocatalytic applications. Regarding to synthetic methodology of HEAs, there is a shift from harsh synthesis conditions to simple and mild synthesis conditions in recent years. Particularly, the recently reported wet chemical synthesis method, which uses reducing agents and structure‐directing agents to construct well‐defined HEAs nanostructures, is a major advance in regulating the morphology and structure of HEAs.

**SCHEME 1:**
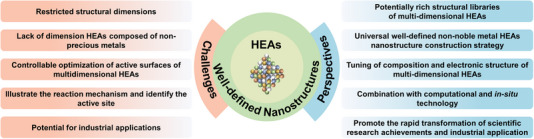
Schematic diagram of challenges and prospects for well‐defined HEAs nanostructured materials in electrocatalysis.

The well‐defined HEAs nanostructures reported so far include regular and uniform nanoparticles, nanowires, nanosheets/nanoplates, core–shell structures, nanoporous and mesoporous nanospheres. For well‐defined HEAs nanostructured electrocatalysts, challenges such as precise regulation of morphology and structure, development of universal synthetic methodologies, and elucidation of reaction mechanisms have seriously hindered the applications of efficient and stable HEAs catalysts. In particular, the catalytic mechanism of HEA catalysts needs to be deeply studied, which will help to accurately identify the active metal center and thus to guide the design of the catalyst. Based on these challenges, we provide some future directions for well‐defined HEAs nanostructure electrocatalysts (Scheme 1).

 

1. Expanding the structural dimensions of HEAs. The electrochemical properties of electrocatalysts are largely affected by their nanostructures. Well‐defined nanostructures bring up large specific surface area and exposed active sites, which effectively enhance the catalytic activity. The currently reported HEAs nanostructures include 0D nanoparticles, 1D nanowires, 2D nanosheets/nanoplates, 3D nanopores, core–shell and mesoporous spheres. In order to expand the structural dimensions of HEAs, different dimensional materials can be combined, such as the combination of 1D and 2D, 1D and 3D, 2D and 3D, so as to enrich the library of structural dimensions of HEAs and widen their applications in the realm of electrocatalysis. Specifically, well‐defined HEAs nanostructures are mainly synthesized by the wet chemical reduction method, and specific morphologies are constructed by selecting different surfactants as structure‐directing agents or different soft template agents. With the purpose of further enriching the structural dimensions of HEAs, a variety of structure‐directing agents or soft templates can be screened during the synthesis process, or different elements can be introduced to construct epitaxial growth on specific crystal planes of HEAs, such as the work on the convex cube Pt_34_Fe_5_Ni_20_Cu_31_Mo_9_Ru HEAs.^[^ ^112^ ^]^
2. Developing multi‐dimensional HEAs with non‐precious metals. Among all metal elements, the Δ*H_mix_* of the atomic combination of Pt with any other element is below the safety upper limit, indicating that Pt more likely forms an alloy with other elements without undergoing phase separation.^[^ ^125^ ^]^ To ensure the successful synthesis of HEAs, most of the papers choose to combine Pt group elements with other components. This inevitably requires the use of high content of precious metals. It is a new challenge to construct a general synthesis route to multi‐dimensional HEAs catalysts composed of non‐noble metals with controllable composition, size, uniformity, and high yield under mild conditions.
3. Optimizing the active surfaces of multidimensional HEAs. The crystal exposure surface and electronic structure of HEAs catalysts, including *d*‐band center and charge transfer, play a pivotal role in electrocatalysis. On the one hand, the exposed surfaces with different crystal structures have different energy barriers for catalytic reactions, and the adjustment of exposed surface sites of catalysts will affect the binding energy of intermediates. On the other hand, through precise adjustment of composition elements and their corresponding concentrations in the alloy, it is possible to finely tune the *d*‐band center of the alloy, thereby regulating the binding energy of key intermediates and ultimately improving the catalytic activity. In addition, the presence of metal components with different work functions in the alloy, facilitates charge transfer between atoms, resulting in significant charge redistribution on the alloy surface, promoting the generation of active sites. Therefore, it is an up‐and‐coming strategy to develop HEAs with specific active surfaces for electrocatalysis.
4. Identifying the real active sites of multi‐dimensional HEAs. HEAs involves multiple components, multiple effects and random distribution of atoms, so the highly complex structure makes their catalytic mechanism unclear. Advanced microstructure characterization techniques, theoretical calculation, in‐situ spectroscopy, and machine learning algorithms can help deepen the understanding of surfactant sites and explore the catalytic mechanism of HEAs catalysts. Particularly, in‐situ techniques can be used to characterize the multi‐dimensional HEAs under real reaction conditions, so that we can have a close look at the reaction and degradation process of the materials.^[^ ^126^ ^]^ And, the electronic states, reaction intermediates and reaction paths of HEAs can be modeled by theoretical calculation. Machine learning algorithms can quickly predict the electron density of crystalline materials and more accurately simulate the surface properties of materials. It opens a new insight for the construction of multi‐dimensional HEAs catalysts with specific active sites.^[^ ^127^ ^]^
5. Promoting the industrial development of multi‐dimensional HEAs. The catalysts of industrial water electrolysis are still mainly composed of precious metals. Compared with traditional alloy catalysts, well‐defined HEAs catalysts have the characteristics of low overpotential, high thermal stability, and fast kinetics. The corrosion resistance and sluggish diffusion effect of HEAs can enhance their stability under industrial conditions. By virtue of the synergistic effect, non‐noble metal‐based HEAs with excellent performances will provide opportunities in industrial hydrogen production. Moreover, owing to its excellent mechanical stability, HEAs can also be used as a support. By anchoring active elements and constructing robust structures favorable to the electrocatalytic reactions on the surface of HEAs, the stability of the catalyst can be improved.^[^ ^128^ ^]^ However, based on the current research, the cooperation of theoretical study and practical application needs to be further strengthened to promote the rapid transformation of scientific research achievements to industrial applications.

In summary, this review summarizes the progresses of well‐defined HEAs nanostructures used in electrocatalysis in terms of the synthetic methodology and structure‐property‐performance relationship in some typical electrocatalytic reactions. In addition, the challenges, and future opportunities for designing nanostructured HEAs electrocatalysts are provided. We hope that this review will provide researchers with an overview of the development of HEAs with different types of well‐defined nanostructures towards electrocatalysis.

## CONFLICT OF INTEREST STATEMENT

The authors declare no conflicts of interest.

## References

[1] a) G. Centi , E. A. Quadrelli , S. Perathoner , Energy Environ. Sci. 2013, 6, 1711;

[2] Y. Chen , T. Cheng , W. A. Goddard, III , J. Am. Chem. Soc. 2020, 142, 8625. 32320243 10.1021/jacs.9b13218

[3] L. Li , P. Wang , Q. Shao , X. Huang , Adv. Mater. 2021, 33, 2004243. 10.1002/adma.202004243 33749035

[4] Y. Li , H. Wang , C. Priest , S. Li , P. Xu , G. Wu , Adv. Mater. 2021, 33, 2000381. 10.1002/adma.202000381 32671924

[5] a) X. Fan , C. Liu , M. Wu , B. Gao , L. Zheng , Y. Zhang , H. Zhang , Q. Gao , X. Cao , Y. Tang , Appl. Catal., B. 2022, 318, 121867;

[6] a) M. E. Kreider , G. T. K. K. Gunasooriya , Y. Liu , J. A. Zamora Zeledón , E. Valle , C. Zhou , J. H. Montoya , A. Gallo , R. Sinclair , J. K. Nørskov , M. B. Stevens , T. F. Jaramillo , ACS Catal. 2022, 12, 10826; 10.1021/acsnano.2c00420 35377139

[7] D. R. Paudel , U. N. Pan , R. B. Ghising , P. P. Dhakal , V. A. Dinh , H. Wang , N. H. Kim , J. H. Lee , Nano Energy 2022, 102, 107712.

[8] a) J. Feng , K. Zhou , C. Liu , Q. Hu , H. Fang , H. Yang , C. He , ACS Appl. Mater. Interfaces 2022, 14, 38717; 35983881 10.1021/acsami.2c08969

[9] a) S. Bian , Q. Liu , X. Zhang , C. Ma , Y. Zhang , Z. Cheng , Y. Kang , W. Lu , P. K. Chu , X. F. Yu , J. Wang , Small 2022, 18, 2203284; 10.1002/smll.202203284 35971184

[10] a) J. N. Hausmann , P. V. Menezes , G. Vijaykumar , K. Laun , T. Diemant , I. Zebger , T. Jacob , M. Driess , P. W. Menezes , Adv. Energy Mater. 2022, 12, 2202098;

[11] S. Shi , B. Wang , Y. Wang , Y. Yang , Z. Zhang , Y. Xu , Y. Suo , Fuel 2022, 330, 125516.

[12] a) X. Mo , X. Gao , A. V. Gillado , H. Y. Chen , Y. Chen , Z. Guo , H. L. Wu , E. C. M. Tse , ACS Nano 2022, 16, 12202; 35959924 10.1021/acsnano.2c02865

[13] a) Q. Wang , Y. Lei , Y. Wang , Y. Liu , C. Song , J. Zeng , Y. Song , X. Duan , D. Wang , Y. Li , Energy Environ. Sci. 2020, 13, 1593;

[14] W. Zhang , P. K. Liaw , Y. Zhang , Sci. China Mater. 2018, 61, 2.

[15] a) T. Wang , A. Chutia , D. J. L. Brett , P. R. Shearing , G. He , G. Chai , I. P. Parkin , Energy Environ. Sci. 2021, 14, 2639;

[16] a) P. Xie , Y. Yao , Z. Huang , Z. Liu , J. Zhang , T. Li , G. Wang , R. Shahbazian‐Yassar , L. Hu , C. Wang , Nat. Commun. 2019, 10, 4011; 31488814 10.1038/s41467-019-11848-9 PMC6728353

[17] a) Y. Luo , S. Hao , S. Cai , T. J. Slade , Z. Z. Luo , V. P. Dravid , C. Wolverton , Q. Yan , M. G. Kanatzidis , J. Am. Chem. Soc. 2020, 142, 15187; 32786784 10.1021/jacs.0c07803

[18] Z. Pu , I. S. Amiinu , R. Cheng , P. Wang , C. Zhang , S. Mu , W. Zhao , F. Su , G. Zhang , S. Liao , S. Sun , Nano‐Micro Lett. 2020, 12, 21. 10.1007/s40820-019-0349-y PMC7770676 34138058

[19] a) K. Zhang , X. Xia , S. Deng , D. Xie , Y. Lu , Y. Wang , J. Wu , X. Wang , J. Tu , J. Energy Chem. 2019, 37, 13;

[20] a) S. Deng , S. Shen , Y. Zhong , K. Zhang , J. Wu , X. Wang , X. Xia , J. Tu , J. Energy Chem. 2017, 26, 1203;

[21] a) Y. Qiao , M. Peng , J. Lan , K. Jiang , D. Chen , Y. Tan , J. Mater. Chem. A 2023, 11, 495;

[22] J.‐T. Ren , L. Chen , H.‐Y. Wang , Z.‐Y. Yuan , Chem. Soc. Rev. 2023, 52, 8319. 37920962 10.1039/d3cs00557g

[23] J. W. Yeh , S. K. Chen , S. J. Lin , J. Y. Gan , T. S. Chin , T. T. Shun , C. H. Tsau , S. Y. Chang , Adv. Eng. Mater. 2004, 6, 299.

[24] B. Cantor , I. T. H. Chang , P. Knight , A. J. B. Vincent , Mater. Sci. Eng., A 2004, 375–377, 213.

[25] Y. Zhang , T. T. Zuo , Z. Tang , M. C. Gao , K. A. Dahmen , P. K. Liaw , Z. P. Lu , Prog. Mater Sci. 2014, 61, 1.

[26] E. P. George , D. Raabe , R. O. Ritchie , Nat. Rev. Mater. 2019, 4, 515.

[27] X. Huang , G. Yang , S. Li , H. Wang , Y. Cao , F. Peng , H. Yu , J. Energy Chem. 2022, 68, 721.

[28] J.‐W. Yeh , Ann. Chim. 2006, 31, 633.

[29] X. Wang , W. Guo , Y. Fu , J. Mater. Chem. A 2021, 9, 663.

[30] J. K. Pedersen , T. A. A. Batchelor , A. Bagger , J. Rossmeisl , ACS Catal. 2020, 10, 2169.

[31] J.‐W. Yeh , Jom 2013, 65, 1759.

[32] J.‐W. Yeh , S.‐Y. Chang , Y.‐D. Hong , S.‐K. Chen , S.‐J. Lin , Mater. Chem. Phys. 2007, 103, 41.

[33] a) Q. Shao , P. Wang , X. Huang , Adv. Funct. Mater. 2019, 29, 1806419;

[34] H. Li , J. Lai , Z. Li , L. Wang , Adv. Funct. Mater. 2021, 31, 2106715.

[35] Y. Yao , Q. Dong , A. Brozena , J. Luo , J. Miao , M. Chi , C. Wang , I. G. Kevrekidis , Z. J. Ren , J. Greeley , G. Wang , A. Anapolsky , L. Hu , Science 2022, 376, eabn3103. 35389801 10.1126/science.abn3103

[36] K. Y. Tsai , M. H. Tsai , J. W. Yeh , Acta Mater. 2013, 61, 4887.

[37] D. B. Miracle , O. N. Senkov , Acta Mater. 2017, 122, 448.

[38] a) K. Li , W. Chen , Mater. Today Energy 2021, 20, 100638;

[39] N. Kumar , C. S. Tiwary , K. Biswas , J. Mater. Sci. 2018, 53, 13411.

[40] Q. Wu , Z. Wang , F. He , L. Wang , J. Luo , J. Li , J. Wang , Metall. Mater. Trans. A 2018, 49, 4986.

[41] Y. Yao , Z. Huang , P. Xie , S. D. Lacey , R. J. Jacob , H. Xie , F. Chen , A. Nie , T. Pu , M. Rehwoldt , D. Yu , M. R. Zachariah , C. Wang , R. Shahbazian‐Yassar , J. Li , L. Hu , Science 2018, 359, 1489. 29599236 10.1126/science.aan5412

[42] S. Gao , S. Hao , Z. Huang , Y. Yuan , S. Han , L. Lei , X. Zhang , R. Shahbazian‐Yassar , J. Lu , Nat. Commun. 2020, 11, 2016. 32332743 10.1038/s41467-020-15934-1 PMC7181682

[43] M. Bondesgaard , N. L. N. Broge , A. Mamakhel , M. Bremholm , B. B. Iversen , Adv. Funct. Mater. 2019, 29, 1905933.

[44] D. Wu , K. Kusada , T. Yamamoto , T. Toriyama , S. Matsumura , S. Kawaguchi , Y. Kubota , H. Kitagawa , J. Am. Chem. Soc. 2020, 142, 13833. 32786816 10.1021/jacs.0c04807

[45] M. Liu , Z. Zhang , F. Okejiri , S. Yang , S. Zhou , S. Dai , Adv. Mater. Interfaces 2019, 6, 1900015.

[46] F. Okejiri , Z. Yang , H. Chen , C.‐L. Do‐Thanh , T. Wang , S. Yang , S. Dai , Nano Res. 2021, 15, 4792.

[47] H. Zhu , Z. Zhu , J. Hao , S. Sun , S. Lu , C. Wang , P. Ma , W. Dong , M. Du , Chem. Eng. J. 2022, 431, 133251.

[48] D. Wu , L. Yao , M. Ricci , J. Li , R. Xie , Z. Peng , Chem. Mater. 2021, 34, 266.

[49] J. Zhu , L. Hu , P. Zhao , L. Y. S. Lee , K. Y. Wong , Chem. Rev. 2020, 120, 851. 31657904 10.1021/acs.chemrev.9b00248

[50] D. Strmcnik , P. P. Lopes , B. Genorio , V. R. Stamenkovic , N. M. Markovic , Nano Energy 2016, 29, 29.

[51] Z. W. Seh , J. Kibsgaard , C. F. Dickens , I. Chorkendorff , J. K. Norskov , T. F. Jaramillo , Science 2017, 355, eaad4998. 28082532 10.1126/science.aad4998

[52] H. Li , Y. Han , H. Zhao , W. Qi , D. Zhang , Y. Yu , W. Cai , S. Li , J. Lai , B. Huang , L. Wang , Nat. Commun. 2020, 11, 5437. 33116124 10.1038/s41467-020-19277-9 PMC7595151

[53] Z. Jin , J. Lv , H. Jia , W. Liu , H. Li , Z. Chen , X. Lin , G. Xie , X. Liu , S. Sun , H. J. Qiu , Small 2019, 15, 1904180. 10.1002/smll.201904180 31596058

[54] D. Wu , K. Kusada , T. Yamamoto , T. Toriyama , S. Matsumura , I. Gueye , O. Seo , J. Kim , S. Hiroi , O. Sakata , S. Kawaguchi , Y. Kubota , H. Kitagawa , Chem. Sci. 2020, 11, 12731. 34094468 10.1039/d0sc02351e PMC8163215

[55] G. Feng , F. Ning , J. Song , H. Shang , K. Zhang , Z. Ding , P. Gao , W. Chu , D. Xia , J. Am. Chem. Soc. 2021, 143, 17117. 34554733 10.1021/jacs.1c07643

[56] D. Feng , Y. Dong , P. Nie , L. Zhang , Z.‐A. Qiao , Chem. Eng. J. 2022, 430, 132883.

[57] J. T. L. Gamler , K. Shin , H. M. Ashberry , Y. Chen , S. L. A. Bueno , Y. Tang , G. Henkelman , S. E. Skrabalak , Nanoscale 2020, 12, 2532. 31932821 10.1039/c9nr09759g

[58] a) L. Zhong , S. Li , ACS Catal. 2020, 10, 4313;

[59] D. Tian , S. R. Denny , K. Li , H. Wang , S. Kattel , J. G. Chen , Chem. Soc. Rev. 2021, 50, 12338. 34580693 10.1039/d1cs00590a

[60] a) Y. Zhu , J. Peng , X. Zhu , L. Bu , Q. Shao , C. W. Pao , Z. Hu , Y. Li , J. Wu , X. Huang , Nano Lett. 2021, 21, 6625; 34319751 10.1021/acs.nanolett.1c02064

[61] Y. Chen , X. Zhan , S. L. A. Bueno , I. H. Shafei , H. M. Ashberry , K. Chatterjee , L. Xu , Y. Tang , S. E. Skrabalak , Nanoscale Horiz. 2021, 6, 231. 33480921 10.1039/d0nh00656d

[62] S. Nellaiappan , N. K. Katiyar , R. Kumar , A. Parui , K. D. Malviya , K. G. Pradeep , A. K. Singh , S. Sharma , C. S. Tiwary , K. Biswas , ACS Catal. 2020, 10, 3658.

[63] D. Zhang , H. Zhao , X. Wu , Y. Deng , Z. Wang , Y. Han , H. Li , Y. Shi , X. Chen , S. Li , J. Lai , B. Huang , L. Wang , Adv. Funct. Mater. 2021, 31, 2006939.

[64] a) M. Gouiza , J. Naliboff , Nat. Commun. 2021, 12, 4653; 34341352 10.1038/s41467-021-24945-5 PMC8329282

[65] G. Zhu , Y. Jiang , H. Yang , H. Wang , Y. Fang , L. Wang , M. Xie , P. Qiu , W. Luo , Adv. Mater. 2022, 34, 2110128. 10.1002/adma.202110128 35146816

[66] a) Y. Y. Birdja , E. Pérez‐Gallent , M. C. Figueiredo , A. J. Göttle , F. Calle‐Vallejo , M. T. M. Koper , Nat. Energy 2019, 4, 732;

[67] F. Pan , Y. Yang , Energy Environ. Sci. 2020, 13, 2275.

[68] J. Graciani , K. Mudiyanselage , F. Xu , A. E. Baber , J. Evans , S. D. Senanayake , D. J. Stacchiola , P. Liu , J. Hrbek , J. F. Sanz , J. A. Rodriguez , Science 2014, 345, 546. 25082699 10.1126/science.1253057

[69] Y. Luo , G.‐F. Chen , L. Ding , X. Chen , L.‐X. Ding , H. Wang , Joule 2019, 3, 279.

[70] a) J. Lim , C. A. Fernández , S. W. Lee , M. C. Hatzell , ACS Energy Lett. 2021, 6, 3676;

[71] J. Chen , H. Cheng , L.‐X. Ding , H. Wang , Mater. Chem. Front. 2021, 5, 5954.

[72] Y.‐f. Yu , W. Zhang , F.‐l. Sun , Q.‐j. Fang , J.‐k. Pan , W.‐x. Chen , G.‐l. Zhuang , Mol. Catal. 2022, 519, 112141.

[73] S. Xu , M. Wang , G. Saranya , N. Chen , L. Zhang , Y. He , L. Wu , Y. Gong , Z. Yao , G. Wang , Z. Wang , S. Zhao , H. Tang , M. Chen , H. Gou , Appl. Catal., B. 2020, 268, 118385.

[74] Y. Mei , Y. Feng , C. Zhang , Y. Zhang , Q. Qi , J. Hu , ACS Catal. 2022, 12, 10808.

[75] S. Wang , W. Huo , F. Fang , Z. Xie , J. K. Shang , J. Jiang , Chem. Eng. J. 2022, 429, 132410.

[76] J. Li , S. Z. Jilani , H. Lin , X. Liu , K. Wei , Y. Jia , P. Zhang , M. Chi , Y. J. Tong , Z. Xi , S. Sun , Angew. Chem., Int. Ed. 2019, 58, 11527. 10.1002/anie.201906137 31206996

[77] a) Y. Wang , M. Zheng , Y. Li , C. Ye , J. Chen , J. Ye , Q. Zhang , J. Li , Z. Zhou , X. Z. Fu , J. Wang , S. G. Sun , D. Wang , Angew. Chem., Int. Ed. 2022, 61, e202115735; 10.1002/anie.202115735 35001467

[78] Q. Tan , C. Shu , J. Abbott , Q. Zhao , L. Liu , T. Qu , Y. Chen , H. Zhu , Y. Liu , G. Wu , ACS Catal. 2019, 9, 6362.

[79] a) Y. Zhang , Y. Shi , R. Chen , L. Tao , C. Xie , D. Liu , D. Yan , S. Wang , J. Mater. Chem. A 2018, 6, 23028;

[80] L. Fan , Y. Ji , G. Wang , J. Chen , K. Chen , X. Liu , Z. Wen , J. Am. Chem. Soc. 2022, 144, 7224. 35404594 10.1021/jacs.1c13740

[81] K. Zeng , J. Zhang , W. Gao , L. Wu , H. Liu , J. Gao , Z. Li , J. Zhou , T. Li , Z. Liang , B. Xu , Y. Yao , Adv. Funct. Mater. 2022, 32, 2204643.

[82] G. Lee , N.‐A. Nguyen , V.‐T. Nguyen , L. L. Larina , E. Chuluunbat , E. Park , J. Kim , H.‐S. Choi , M. Keidar , J. Solid State Chem. 2022, 314, 123388.

[83] Y. Lu , K. Huang , X. Cao , L. Zhang , T. Wang , D. Peng , B. Zhang , Z. Liu , J. Wu , Y. Zhang , C. Chen , Y. Huang , Adv. Funct. Mater. 2022, 32, 2110645.

[84] H. Minamihara , K. Kusada , D. Wu , T. Yamamoto , T. Toriyama , S. Matsumura , L. S. R. Kumara , K. Ohara , O. Sakata , S. Kawaguchi , Y. Kubota , H. Kitagawa , J. Am. Chem. Soc. 2022, 144, 11525. 35749353 10.1021/jacs.2c02755

[85] X. Wang , Q. Peng , X. Zhang , X. Lv , X. Wang , Y. Fu , J. Colloid Interface Sci. 2022, 607, 1580. 34587532 10.1016/j.jcis.2021.08.201

[86] H. Chen , C. Guan , H. Feng , ACS Appl. Nano Mater. 2022, 5, 9810.

[87] K. Huang , D. Peng , Z. Yao , J. Xia , B. Zhang , H. Liu , Z. Chen , F. Wu , J. Wu , Y. Huang , Chem. Eng. J. 2021, 425, 131533.

[88] J. Huang , P. Wang , P. Li , H. Yin , D. Wang , J. Mater. Sci. Technol. 2021, 93, 110.

[89] C. Cai , Z. Xin , X. Zhang , J. Cui , H. Lv , W. Ren , C. Gao , B. Cai , Catalysts 2022, 12, 1050.

[90] Y. Yu , F. Xia , C. Wang , J. Wu , X. Fu , D. Ma , B. Lin , J. Wang , Q. Yue , Y. Kang , Nano Res. 2022, 15, 7868.

[91] R. Nandan , G. Raj , K. K. Nanda , ACS Appl. Mater. Interfaces 2022, 14, 16108. 35357120 10.1021/acsami.1c21336

[92] D. Wang , Z. Chen , Y.‐C. Huang , W. Li , J. Wang , Z. Lu , K. Gu , T. Wang , Y. Wu , C. Chen , Y. Zhang , X. Huang , L. Tao , C.‐L. Dong , J. Chen , C. V. Singh , S. Wang , Sci. China Mater. 2021, 64, 2454.

[93] S. L. Zhang , X. F. Lu , Z. P. Wu , D. Luan , X. W. D. Lou , Angew. Chem., Int. Ed. 2021, 60, 19068. 10.1002/anie.202106547 34137497

[94] C. Zhan , Y. Xu , L. Bu , H. Zhu , Y. Feng , T. Yang , Y. Zhang , Z. Yang , B. Huang , Q. Shao , X. Huang , Nat. Commun. 2021, 12, 6261. 34716289 10.1038/s41467-021-26425-2 PMC8556242

[95] S. Li , J. Wang , X. Lin , G. Xie , Y. Huang , X. Liu , H. J. Qiu , Adv. Funct. Mater. 2021, 31, 2007129.

[96] a) W. Zhang , Y. Yang , B. Huang , F. Lv , K. Wang , N. Li , M. Luo , Y. Chao , Y. Li , Y. Sun , Z. Xu , Y. Qin , W. Yang , J. Zhou , Y. Du , D. Su , S. Guo , Adv. Mater. 2019, 31, 1805833; 10.1002/adma.201805833 30803065

[97] H. Li , M. Sun , Y. Pan , J. Xiong , H. Du , Y. Yu , S. Feng , Z. Li , J. Lai , B. Huang , L. Wang , Appl. Catal., B. 2022, 312, 121431.

[98] D. Fan , K. Guo , Y. Zhang , Q. Hao , M. Han , D. Xu , J. Colloid Interface Sci. 2022, 625, 1012. 35803135 10.1016/j.jcis.2022.06.105

[99] Z. Jin , J. Lyu , Y.‐L. Zhao , H. Li , X. Lin , G. Xie , X. Liu , J.‐J. Kai , H.‐J. Qiu , ACS Mater. Lett. 2020, 2, 1698.

[100] a) F. Saleem , B. Xu , B. Ni , H. Liu , F. Nosheen , H. Li , X. Wang , Adv. Mater. 2015, 27, 2013; 25677842 10.1002/adma.201405319

[101] X. Fu , J. Zhang , S. Zhan , F. Xia , C. Wang , D. Ma , Q. Yue , J. Wu , Y. Kang , ACS Catal. 2022, 12, 11955.

[102] A. Mahmood , N. Xie , M. A. Ud Din , F. Saleem , H. Lin , X. Wang , Chem. Sci. 2017, 8, 4292. 28626567 10.1039/c7sc00318h PMC5468992

[103] C. Zhan , L. Bu , H. Sun , X. Huang , Z. Zhu , T. Yang , H. Ma , L. Li , Y. Wang , H. Geng , W. Wang , H. Zhu , C. W. Pao , Q. Shao , Z. Yang , W. Liu , Z. Xie , X. Huang , Angew. Chem., Int. Ed. 2023, 62, e202213783. 10.1002/anie.202213783 36400747

[104] M. A. Ud Din , F. Saleem , B. Ni , Y. Yong , X. Wang , Adv. Mater. 2017, 29, 1604994. 10.1002/adma.201604994 27943480

[105] G. Ferey , Chem. Soc. Rev. 2008, 37, 191. 18197340 10.1039/b618320b

[106] a) B. C. Tappan , S. A. Steiner, 3rd, E. P. Luther , Angew. Chem., Int. Ed. 2010, 49, 4544; 10.1002/anie.200902994 20514651

[107] H. Liu , H. Qin , J. Kang , L. Ma , G. Chen , Q. Huang , Z. Zhang , E. Liu , H. Lu , J. Li , N. Zhao , Chem. Eng. J. 2022, 435, 134898.

[108] R. Q. Yao , Y. T. Zhou , H. Shi , W. B. Wan , Q. H. Zhang , L. Gu , Y. F. Zhu , Z. Wen , X. Y. Lang , Q. Jiang , Adv. Funct. Mater. 2021, 31, 2009613.

[109] H.‐J. Qiu , G. Fang , Y. Wen , P. Liu , G. Xie , X. Liu , S. Sun , J. Mater. Chem. A 2019, 7, 6499.

[110] S. Li , X. Tang , H. Jia , H. Li , G. Xie , X. Liu , X. Lin , H.‐J. Qiu , J. Catal. 2020, 383, 164.

[111] H.‐J. Qiu , G. Fang , J. Gao , Y. Wen , J. Lv , H. Li , G. Xie , X. Liu , S. Sun , ACS Mater. Lett. 2019, 1, 526.

[112] Z. Chen , J. Wen , C. Wang , X. Kang , Small 2022, 18, 2204255. 10.1002/smll.202204255 36161488

[113] a) Z. Mei , Y. Li , M. Fan , L. Zhao , J. Zhao , Chem. Eng. J. 2015, 259, 293;

[114] X. Wang , Q. Dong , H. Qiao , Z. Huang , M. T. Saray , G. Zhong , Z. Lin , M. Cui , A. Brozena , M. Hong , Q. Xia , J. Gao , G. Chen , R. Shahbazian‐Yassar , D. Wang , L. Hu , Adv. Mater. 2020, 32, 2002853. 10.1002/adma.202002853 33020998

[115] X. Zuo , R. Yan , L. Zhao , Y. Long , L. Shi , Q. Cheng , D. Liu , C. Hu , J. Mater. Chem. A 2022, 10, 14857.

[116] a) R. Du , J. O. Joswig , R. Hubner , L. Zhou , W. Wei , Y. Hu , A. Eychmuller , Angew. Chem., Int. Ed. 2020, 59, 8293; 10.1002/anie.201916484 PMC7317422 32187791

[117] a) Y. Li , C. K. Peng , H. Hu , S. Y. Chen , J. H. Choi , Y. G. Lin , J. M. Lee , Nat. Commun. 2022, 13, 1143; 35241652 10.1038/s41467-022-28805-8 PMC8894469

[118] H. Li , H. Huang , Y. Chen , F. Lai , H. Fu , L. Zhang , N. Zhang , S. Bai , T. Liu , Adv. Mater. 2023, 35, 2209242. 10.1002/adma.202209242 36373568

[119] Y. Kang , O. Cretu , J. Kikkawa , K. Kimoto , H. Nara , A. S. Nugraha , H. Kawamoto , M. Eguchi , T. Liao , Z. Sun , T. Asahi , Y. Yamauchi , Nat. Commun. 2023, 14, 4182. 37443103 10.1038/s41467-023-39157-2 PMC10344865

[120] Y. Wang , B. Yu , M. He , Z. Zhai , K. Yin , F. Kong , Z. Zhang , Nano Res. 2022, 15, 4820.

[121] Z. X. Cai , H. Goou , Y. Ito , T. Tokunaga , M. Miyauchi , H. Abe , T. Fujita , Chem. Sci. 2021, 12, 11306. 34667541 10.1039/d1sc01981c PMC8447928

[122] J. Tang , J. L. Xu , Z. G. Ye , X. B. Li , J. M. Luo , J. Mater. Sci. Technol. 2021, 79, 171.

[123] L.‐H. Liu , N. Li , M. Han , J.‐R. Han , H.‐Y. Liang , Rare Met. 2022, 41, 125.

[124] X. Chen , C. Si , Y. Gao , J. Frenzel , J. Sun , G. Eggeler , Z. Zhang , J. Power Sources 2015, 273, 324.

[125] Y. Wang , W. Luo , S. Gong , L. Luo , Y. Li , Y. Zhao , Z. Li , Adv. Mater. 2023, 35, 2302499. 10.1002/adma.202302499 37155729

[126] H. Sun , X. Xu , H. Kim , Z. Shao , W. Jung , InfoMat 2024, 6, e12494.

[127] Y. Wang , M. J. Robson , A. Manzotti , F. Ciucci , Joule 2023, 7, 848.

[128] R. Zhou , X. Han , Q. Chen , L. Peng , X. Qiu , P. Wang , C. Guo , J. Wang , Z. Wang , J. Hao , J. Mater. Chem. A 2024, 12, 5719.

